# Supplementary material for: Environmental temperatures shape thermal physiology as well as diversification and genome-wide substitution rates in lizards
Source: Nat Commun. 2019 Sep 9;10:4077. doi: 10.1038/s41467-019-11943-x (PMC6733905; doi:10.1038/s41467-019-11943-x)
Supplement: Supplementary file 1 — Supplementary Information [file 41467_2019_11943_MOESM1_ESM.pdf]

Supplementary Information

Environmental temperatures shape thermal physiology as well as diversification and genome-wide substitution rates in lizards

Garcia-Porta, Irisarri, et al.

Supplementary methods

Phylogenomic data set assembly

This study is mainly based on two new phylogenomic data sets (RNAseq and AHE), for which we especially selected lacertid species to represent the major clades of Lacertini that so far have evaded reliable phylogenetic reconstruction. As these two data sets contained different selections of taxa, they were first analyzed separately. To create a taxonomically more comprehensive tree for downstream analyses, both phylogenomic datasets were combined with five gene fragments commonly used in lacertid phylogenetics and available for a large number of species. The amphisbaenian, *Blanus cinereus,* known to represent the sister group of Lacertidae [1], was used as an outgroup in all phylogenetic analyses.

The Anchored Hybrid Enrichment (AHE) method [2] was used to target 394 anonymous nuclear loci (of which 324 loci were retained for analysis). To generate the AHE data set, genomic DNA was extracted from fresh or ethanol-preserved tissue samples with a Qiagen DNeasy® Tissue Kit, DNA concentration measured with a Qubit® fluorometer, and samples with > 12 µg/ml selected for enrichment and sequencing in the Center for Anchored Phylogenomics at Florida State University (www.anchoredphylogeny.com), following previously outlined methods [2,3]. Briefly, genomic DNA was sonicated to a fragment size of 200–800 bp and libraries prepared and indexed following the protocol of Meyer and Kircher [4] with minor modifications [2], using a Beckman-Coulter Biomek FXp liquid-handling robot. Following library preparation, libraries were pooled in equal quantities in batches of 16 libraries. Equal quantities of indexed samples were pooled and enrichments were performed with probes designed for anchored loci from amniotes [3,5,6], specifically using the Squamate-optimized kit [6]. Sequencing was carried out on two lanes of an Illumina HiSeq2500 sequencer with PE150 sequencing (~33 samples per lane, 2 lanes, 98 gigabases total).

After quality filtering using the high chastity setting in Illumina's Casava software, the reads were de-multiplexed using the 8 bp indexes, with zero mismatches tolerated. Paired reads were then merged using a Bayesian approach [7]. This approach trims adapters and corrects sequencing errors in overlapping regions. Reads were assembled using a quasi-*de novo* approach [8] but with *Anolis* and *Salvator* probe region sequences serving as references for the assembly [6]. In order to avoid the effects of possible contamination and/or misindexing, assembled contigs derived from fewer than 100 reads were removed prior to downstream analyses. Contigs passing this filter were then assessed for orthology using pairwise distances [8]. Orthologous sequences were aligned using MAFFT (v. 7.023b) [9] then trimmed/ masked, with MINGOODSITES=14, MINPROPSAME=0.5, and MISSINGALLOWED=35 [8]. Alignments were manually inspected in Geneious R9 (Biomatters Ltd.) [10] in order to identify misaligned regions, which were masked prior to phylogenetic analyses.

New transcriptomic (RNAseq) data were generated from from 100 mg of tissue of each lizard, consisting of combined or separate skin, muscle, or liver samples preserved in RNAlater and frozen at -80°C. RNA extraction was carried out using standard trizol protocols. Sequencing was

carried out with a High Output 150 cycle kit. Illumina reads were quality-trimmed and filtered using Trimmomatic v. 0.32 [11] with default settings and later filtered for rRNA sequences with SortMeRNA [12]. Paired and unpaired filtered reads were used for *de novo* transcriptome assembly using Trinity v. 2.1.0 [13] following published protocols [14]. Candidate coding regions within transcript sequences from the final assembly were identified and translated using Transdecoder 2.1.0 [14].

We used a previously compiled alignment of 4593 + 1506 + 1195 nuclear genes [15] as a reference for ortholog selection, hereafter reference set. Coding sequences of the new transcriptomes identified by Transdecoder were aligned to the reference set using the software Forty-Two (D. Baurain; https://bitbucket.org/dbaurain/42/). Sequence decontamination and paralog resolution followed the previously established protocol [15]. Briefly, contaminant sequences from non-vertebrate sources (e.g. Apicomplexa or Nematodes) were detected by BLAST searches and removed. For each species and locus, redundant and/or divergent sequences were eliminated by comparing every sequence against all other sequences in the alignment using BLAST. Sequences with average bit scores at least 10% lower than the best average bit score and with ≥95% sequence overlap to other redundant sequences were eliminated. Paralogs were identified and resolved using a custom script [15].

Final alignments contained only lacertid sequences as well as *Blanus cinereus* used as outgroup. We selected a total of 6,556 loci containing at least 15 lacertids. The corresponding nucleotide sequences for the retained amino acid alignments were recovered using the program leel (Denis Baurain, unpublished), and aligned according to the amino acid alignments. All subsequent analyses were based on nucleotide alignments. A final quality filtering was performed by comparing every branch length of single gene trees to the corresponding branch in the concatenated ML tree (inferred under GTR+Γ with RAxML v.8 [16]) in order to identify and remove problematic sequences not captured in previous decontamination steps [15]. A total of 2,352 individual sequences were removed in the following way: 961 sequences displaying terminal branches on gene trees 9 times longer than the concatenated ML tree and 1,391 sequences displaying a terminal branch 5 times longer. Finally, 252 gene alignments were discarded based on a too low coefficient of correlation between gene branch length and concatenation branch length (i.e. below the mean -1.96 SD of the observed R, in practice 0.65). The final dataset contained a total of 6,269 genes for 24 lacertids (22 species; two species had two individuals each) and one outgroup, each containing at least 15 lacertid sequences.

DNA sequences from previous studies for the nuclear proto-oncogene mos gene (*c-mos*) and the mitochondrial genes for 12S and 16S ribosomal RNA (12S, 16S), Cytochrome b (cob), and NADH-dehydrogenase subunit 4 (nd4) were retrieved manually from Genbank based on the most recent publications, to ensure that for taxa that underwent taxonomic revision the sequences included in the supermatrix were correctly assigned.

Phylogenetic analyses

For each of the resulting AHE and RNASeq datasets as well as the combined dataset including five additional genes (12S, 16S, cob, nd4, c-mos) (hereafter AHE, RNASeq, and combined, respectively) we performed ML phylogenetic inference analyses using IQTREE v. 1.5.4 [17]. We selected best-fitting partitioning scheme and substitution models by AIC assuming edge-proportional rates and using a relaxed hierarchical clustering approach, as implemented in IQTREE ("-spp -m MFP+MERGE –rcluster 10" options). The -spp option specifies branch lengths as linked, evolutionary rates as unlinked. Node support was estimated in IQTREE with 1,000 replicates of ultrafast bootstrapping [18].

Because of the large size of the combined matrix (247 species and 6,598 loci) and high

proportion of missing data (47%), best-fit partition finding across the full parameter space proved computationally infeasible even on high-performance clusters. We then applied two alternative approaches, (i) heuristic search restricting the set of candidate models to GTR, HYK or JC, with or without discrete Γ distribution to model among site rate heterogeneity in order to represent the parameter space by the simplest and the most complex models available ("-m TESTMERGE –mset JC, HKY, GTR –mrate E,G –rcluster 1"), and (ii) using the best-fit partitions previously applied for the separate analyses of the RNAseq and AHE datasets, and the newly estimated best-fit partitions for the legacy gene data set. In final analyses, we allowed each partition to have either JC, HKY or GTR models, with or without gamma parameter ("-m TEST -mset JC, HKY, GTR -mrate E,G"). Based on empirical evaluation of the suggested partition settings and resulting trees we chose the second alternative for our final analysis.

Conflicting genealogical histories can confound phylogenetic inferences of concatenated multi-locus matrices potentially resulting in incorrect trees with high support [19] and this can be exacerbated in phylogenomic-level analyses [20]. Therefore, we estimated species trees from the two phylogenomic data sets under the multi-species coalescence model as implemented in ASTRAL II [21]. ASTRAL was fed with locus trees estimated under the best-fitting model as estimated in IQTREE with 1,000 ultrafast bootstrap replicates. Branch support was assessed by multilocus bootstrapping using the gene and site re-sampling strategy, as implemented in ASTRAL II [22].

Phylogenetic placement of fossils and molecular dating

The fossil record of Lacertidae mostly consists of fragmentary cranial bones and isolated postcranial elements, often making reliable taxonomic or systematic assignments difficult. We therefore included only the following lacertid fossils in our morphological data set, some of which we also microCT scanned:

1) *Dracaenosaurus croizeti* [23] (scored for 27 cranial characters), based on an excellently preserved skull partly embedded in the matrix from the upper Oligocene Cournon, Department of Puy-de Dome, France (MNHN.F.COR7, microCT scanned, see also [24]).

2) *Succinilacerta succinea* from Eocene Baltic amber [25], type specimen (MGPU 12664; scored for 11 scalation characters), an almost complete lizard amber inclusion from the Sambia quarry, Kaliningrad, Russia.

3) *Succinilacerta succinea* from Gdansk-stogi, Poland (G.G.1; scored for 12 scalation characters; see also [26]) an almost complete amber inclusion with head, throat, forelimbs, hindlimbs, part of the tail and some claws.

4) Lacertidae indet. (*?"Succinilacerta"*) Deutsches Bernsteinmuseum collection no. 1119 (scored for 15 scalation characters) a well preserved amber inclusion of a lacertid lizard head, the exact locality of which, other than Baltic amber, is unknown. All the amber specimens listed were microCT scanned.

5) *Janosikia ulmensis* [27] from the early Miocene of Ulm Germany (scored for 16 characters), consisting mostly of cranial elements.

6) *Plesiolacerta eratothemesi* [28] from the upper Oligocene of Herlingen, Germany, scored for 5 characters for dentary and maxilla.

7) The Paleogene *Plesiolacerta lydekkeri* [29] from France, scored for 19 characters for dentary, maxilla, frontal, and parietal.

8) "*Lacerta viridis group"* from the early Miocene of Dolnice near Cheb, Czech Republic, scored for 6 characters for the frontal (see also [30]).

9) *Pseudeumeces cardurcensis* [31] from the early Oligocene of France [32]. Scored for 17 characters for dentary, maxilla, and frontal.

Our morphological character matrix consisted of 89 characters for the nine fossil taxa listed

above and 82 extant taxa (from 36 genera). 49 characters were taken from the literature, sometimes modified, and 30 new characters were defined (see Supplementary Table 2 for details). With a few exceptions, we scored 3-5 specimens per species. Character selection from the literature was based on the criterion that ideally there should be not too much intraspecific variation in the respective states, which excluded some characters used in previous studies. Linear measurements on the 3D microCT scans were sometimes used to redefine some traditional characters more precisely so as to find quantitative thresholds that would allow for a distinction between different character states. The final matrix consisted of characters referring to the cranium (34 characters), mandible (13), postcranium (8), head scalation (11), body scalation (12), and soft tissue and behaviour (12). Specimens were digitized at the Museum für Naturkunde Berlin, using a Phoenix nanotom X-ray|s tube at 70-80KV and 110 to 150µA, generating 1000 projections with 750ms per scan. Parameters varied depending on specimen size and result in an effective voxel size of 6 to 12µm. The cone beam reconstruction was performed using the datos|x-reconstruction software (GE Sensing & Inspection Technologies GmbH poenix|x-ray datos|x 2.0) and data were visualized in Volume Graphics Studio Max 3.1 and Meshlab (meshlab.net). To define plesiomorphic character states we used the amphisbaenian *Blanus cinereus* and the teiid *Tupinambis teguixin* as outgroup taxa.

The combined molecular tree with wide taxon coverage (Supplementary Fig. S8) was pruned using R [33] and the R packages ′phytools′ [34] and ′ape′ [35] in order to fit the taxon selection of our morphological data set. We then used the software package TNT 1.5.3 [36] to run a phylogenetic analysis using parsimony, with the molecular topology used as a topological constraint for the extant taxa and with the fossils defined as "floaters". We applied two different search algorithms, i.e. the traditional tree search and the more advanced "new technology search". For the latter we used the options "ratchet" and "drift" and set the initial level to 75, which enables the algorithm to optimize parameters automatically. To ensure statistical robustness, tree search was repeated 100 times. Our analyses resulted in 100 and 18 most parsimonious trees, respectively (i.e. using traditional or new technology search; tree length: 1,167 steps; Consistency Index: 0.105; Retention Index: 0.484) with the "*Lacerta viridis* group" fossil and *Plesiolacerta eratothemesi* being placed all over the tree. We concluded that the only 5 and 6 scored characters available for these fossils, respectively, are likely to be insufficient to find a stable position, so these taxa were excluded from further analyses. A second search resulted in 70 (traditional) 11 (new technology) trees (tree length: 1,166 steps; Consistency and Retention Index same as above). The general topology of the consensus trees was identical and is presented in Supplementary Figure 9. Notable results are the polyphyly of the amber lacertids which were all placed within Eremiadini, the recovery of a clade consisting of *Dracaenosaurus/Pseudeumeces/Janosikia* as sister group to *Gallotia*, which confirms previous hypotheses [1,24], the unstable position of *Plesiolacerta lydekkeri* while still being placed only within Lacertinae, and the lack of a reliable placement of the "*Lacerta viridis* group" fossil within the genus *Lacerta,* thus questioning the previous assignment [30].

On the basis of the results from the total evidence analysis the following fossil calibration points were defined for molecular dating:

Lacertidae (Gallotiinae – Lacertinae): We used the mid Eocene age of 40.4 Ma as a minimum age for this split based on the oldest record of *Plesiolacerta lydekkeri*, which comes from Lissieu, France (MP 14 [28]). Even though *Plesiolacerta lydekkeri* could not be confidently placed in our phylogeny, it nevertheless clustered consistently within Lacertinae. As maximum age we used 150.0 Ma, which is based on the median age estimate for crown Lacertoidea as given in Jones et al. [37].

Lacertinae (Lacertini – Eremiadini): As minimum age we used 33.9 Ma, i.e. the end of the Eocene (Priabonian), which is based on several different lacertid fossils from Baltic Amber, especially the Göttingen (and type) specimen of *Succinilacerta succinea* from the Sambia Quarry, Kaliningrad, Russia. All fossil amber lacertids fell within Eremiadini in our phylogenetic analysis.

For a long time, the age of the Baltic amber deposits was disputed, but there is increasing support for a Late Eocene age (Alexander Schmidt, University of Göttingen, pers. comm.; [38]). As maximum age we used 61.6 Ma, which is based on the oldest-known lacertid fossils, "cf. *Plesiolacerta* sp." and "Lacertidae gen. indet." from Hainin, Mons Basin, Province de Hainaut, Belgium, which has been dated as Paleocene (Danian; 61.6 Ma [39]). Due to poor preservation we did not include these fossils in our phylogenetic analysis, but since they are indisputably lacertids we decided to use them as a potential maximum age for the splits within crown Lacertidae.

Gallotiinae (*Gallotia – Psammodromus*): We assigned the Oligocene (Rupelian) date of 28.1 Ma as minimum age to this clade, based on the fossil clade consisting of *Dracaenosaurus/Pseudeumeces/Janosikia*, which consistently fell as sister group to the extant genus *Gallotia* in our analysis. The oldest record for this clade dates from the Rupelian of the Phosphorites du Quercy, France (*Pseudeumeces* [32]). As maximum age we used 61.6 Ma, the age of the oldest-known fossil evidence of lacertids (see above).

Lacerta s. str. (*Lacerta agilis – Lacerta viridis*): The minimum age for this split (4.4. Ma) is based on the oldest-known reliable fossil of the *Lacerta agilis* lineage, which comes from the Pliocene of Ivanovce, Slovakia [40]. We decided not to use the fossil lacertids originally described as members of the *Lacerta viridis* group from the Lower Miocene of the Czech Republic for calibration [30], although they had been previously used for molecular dating [41], because of their uncertain position in our phylogenetic analysis. Also in the original description, the systematic placement of these fossils was not tested phylogenetically. As maximum age we used 61.6 Ma, the age of the oldest-known fossil evidence of lacertids (see above).

Acanthodactylus erythrurus – A. lineomaculatus: This is a modified calibration taken from [41], with the minimum age representing the oldest-known record of *A. erythrurus* from the Pleistocene of Spain [42], and the maximum age being 61.6 Ma, that of the oldest-known fossil evidence of lacertids (see above).

For the final dating analysis, we used a penalized likelihood approach as implemented in the software package TreePL 1.0 [43]. Using the ′prime′ command, which allows the program to find the optimal parameters for a given data set, we obtained the time tree as shown in Supplementary Figure 10 and used in downstream analyses. In this tree, especially the ages of the root and the splits of the major clades are older than in previous studies [41,44]. However, in the latter analyses the root was constrained to a younger age due to the lack of reliable lacertid or amphisbaenian fossils from the Mesozoic. In our present analysis we did not implement such a constraint, which likely resulted in older ages of especially the early splits. Roughly similar ages were found previously [45,46,47], whereas these authors did not focus on lacertid ingroup relationships and respective age estimates. With respect to the latter, a deep Paleogene split between Eremiadini and Lacertini has been proposed previously [44].

Experimental assessment of ecophysiological traits

Evaporative water loss and preferred body temperature, were assessed for a target sample size of at least 10 adult males per population, and sometimes several populations per species, with lower sample sizes for a few species only (Supplementary Table 3).

We measured instant evaporative water loss, IWL, housing lizards individually in plastic boxes with holes at top, bottom, and sides, to ensure airflow [48]. Each box was placed into a second box provided with some silica gel on the bottom. All individual boxes were placed in a large container with sufficient silica gel to maintain low air humidity values between 10-20%, and the container placed at a constant temperature of 20-22°C in the dark. Because water loss is dependent on body surface which decreases disproportionally with mass, we calculated lizard body surface [49] as ln[surface area] = 2.36 + (0.69 * ln[mass]). IWL was regressed against ln[surface area] and the residuals used as surface-corrected IWL. This formula is only an

approximation without correcting for different lizard body shapes (elongated vs. stout). Its use is however justified, considering that its main purpose was to correct for surface area in calculating IWL among lacertid species that vary over a large range of body sizes and masses (0.5-115 g), leaving body shape effects as a secondary, minor factor.

The IWL data set was carefully quality-checked following a series of steps and rules as follows: (1) Weights preceding and following verified instances of defecation or urination were excluded from calculations. (2) When negative values of IWL were found (i.e., a weight increase over time), this was interpreted as measurement error and the respective value was deleted. (3) For those weight measurements where defecation was noted during the experiment, we deleted the IWL values for this and the following time points. (4) For all used values of IWL, we identified IWL outlier values being 3 SD or more above the average, separately for each species. While some of these might correspond to actual episodes of high evaporative water loss, possibly due to greater stress at the beginning of the experiments, many of these cases probably correspond to unnoticed instances of defecation or urination. These values were therefore removed as well together with the subsequent value obtained from this specific lizard. Subsequently, we also removed all values from data points 1 and 2, to exclude the possibility that the measured water loss was superficial. That is, the lizards might have been included in the experiments while still carrying moisture on their body surface or between the scales; the evaporation of this water would then have led to high initial water loss in some individuals. (5) We also removed the columns corresponding to time points 11 and 12, given the large amounts of missing data for these time points. (6) Eventually, we searched one more time for outliers, highlighting values higher than 3 x SD per genus, and removed a few that were clearly different from other values for the same individual or population. These were usually values at least 3 x higher than the next highest value for the same specimen.

To estimate selected body temperature as a proxy for preferred body temperature (T_{pref}) in a photothermal gradient we used an experimental setup consisting typically of eight individual tracks (910 x 380 x 120 mm [50]). Before the experiments, lizards were kept for several hours at room temperature (20-25°C). An incandescent 100 W light bulb (full spectrum) was suspended 30 cm above one end to create a thermal gradient of approximately 20° to 55°C at ground level. Lizards were allowed unrestricted movement within their individual gradient. Lizard body temperature was measured over a period of up to 2.5 consecutive hours. The initial 20-30 minutes of the experiment were discarded as acclimation time. Body temperature was determined every minute by ultra-thin T-type thermocouples (OMEGA 5SCTT-T-40-72, diameter = 0.076 mm, Norwalk, Connecticut, USA) affixed with medical tape to the lizards' venter and connected to an 8-Channel USB Thermocouple Data Acquisition Module (OMEGA TC-08; resolution <0.1°C). For each individual, we removed data points representing clear inactivity periods or detached thermocouples, and subsequently determined the average of all body temperature measurements.

We consistently applied this method to be able to integrate previously obtained data [50] and keep methodological consistency. It must be emphasized that T_{pref} measures differ if taken by alternative approaches. For instance, some of the coauthors of this study have argued that using an infrared light bulb is the optimal way to create thermal gradients because it exempts the light spectrum, which may disrupt the lizards' photoperiod, and furthermore have used hourly cloacal temperature measurements rather than per-minute measurements over two hours as herein [48,51–54]. From those lacertid species where T_{pref} measurements were obtained using the two alternative methods (incandescent bulb / 2 h experiment with T_b measured once a minute vs. infrared bulb / measurements 12 h experiments with T_b measured hourly) we assessed that the incandescent bulb method consistently results in higher T_{pref} estimates. The differences were between 0.06–6.30°C, with an average difference of 0.941°C (Supplementary Table 12). The wide range of variation certainly also reflects differences in the behaviour of the lizards, and the highest difference of 6.3°C refers to a case of very low T_{pref} values in *Darevskia praticola* in the infrared bulb setup). We therefore included in our study only two lizard species with data from the infrared bulb

approach since no other data were available for the respective genera: *Ophisops elegans* and *Hellenolacerta graeca*. Based on our empirical comparisons (see above and Supplementary Table 12) we added 1°C ad-hoc to the values for these species to adjust their data to the remaining thermocouple-based data set. Furthermore, for five species where only single or small numbers of males were used in the experiments, we complemented the data with T_{pref} estimates of 14 females (*Anatololacerta oertzeni, Iberolacerta galani, Lacerta trilineata, Psammodromus algirus, Timon lepidus*; see Supplementary Table 13 for a summary of male-female differences in these species).

Based on exploratory analysis of the resulting data set we identified specimens with unrealistic T_{pref} data. As such we considered all individual median values below 32°C and above 38°C, especially when these were found for species with very large min-max and quartile ranges. For all individuals with median $T_{pref,}$ values between 32-38°C we examined the original temperature curves of the respective experiments, and discarded those that showed unrealistic curves. These indicated very little variation and thus basically no movement at low body temperature, suggesting extended periods of inactivity.

In order to obtain single variables per species for these physiological variables for comparative phylogenetic analyses we used median values per individual, and from these subsequently calculate medians per species. This approach simplifies the data by not considering the existence of variation among individuals and populations, which in itself is an important trait. We used medians to circumvent non-normality of values, within-individuals due to phases of different behaviour, as well as within-species due to inclusion of specimens from different populations. In-depth analysis of the physiological variation within or across species is beyond the scope of this paper, but could reveal important differences in physiological plasticity among lacertid taxa. Furthermore, acclimation in some lizard species has been identified as an important factor shaping their physiological preferences. Yet, constant differences between lizard species in physiological parameters have been thoroughly assessed, and the differences in both IWL and T_{pref} found in this study greatly exceed the amount of adaptation- and acclimation-related intraspecific variation known from lizards.

Compilation of bioclimatic data set and analysis of species richness patterns

The initial set of lacertid occurrence records was manually curated by a series of procedures. All records were mapped by species and, if available, compared with range maps of lacertid species from the International Union for Conservation of Nature (IUCN) at www.redlist.org. Records at distances of approximately >30 km from known range boundaries were only kept if plausible through our own observations and assessments of a species' biogeography, deriving from taxonomic revisions or specific publications, or otherwise verified. Records of European species were furthermore verified with the aid of the European Herpetological Atlas [55].

Main climatic zones were defined according to Köppen-Geiger categories [56], i.e., equatorial, arid, warm temperate, snow, polar.

We extracted 29 bioclimatic variables for temperature, precipitation and solar radiation, obtained from WorldClim [57,58] at 30 arc-second resolution, associated with all data points for each species using the package "raster" [59] and calculated medians per species: bio1 = annual mean temperature (°C); bio2 = mean diurnal range (mean of monthly (max temp - min temp)) (°C); bio3 = isothermality (bio2/bio7) (* 100); bio4 = temperature seasonality (standard deviation *100); bio5 = max temperature of warmest month (°C); bio6 = min temperature of coldest month (°C); bio7 = temperature annual range (bio5-bio6) (°C); bio8 = mean temperature of wettest quarter (°C); bio9 = mean temperature of driest quarter (°C); bio10 = mean temperature of warmest quarter (°C); bio11 = mean temperature of coldest quarter (°C); bio12 = annual precipitation (mm); bio13 = precipitation of wettest month (mm); bio14 = precipitation of driest month (mm); bio15 = precipitation seasonality (coefficient of variation); bio16 = precipitation of

wettest quarter (mm); bio17 = precipitation of driest quarter (mm); bio18 = precipitation of warmest quarter (mm); bio19 = precipitation of coldest quarter (mm); bio20 = annual mean radiation (W m-2); bio23 = radiation seasonality (C of V); bio24 = radiation of wettest quarter (W m-2); bio25 = radiation of driest quarter (W m-2); bio26 = radiation of warmest quarter (W m-2); bio27 = radiation of coldest quarter (W m-2); bioSR1 = mean precipitation of quarter of least solar radiation; bioSR2 = mean precipitation of quarter of most solar radiation; bioSR3 = mean temperature of quarter of least solar radiation; bioSR4 = mean temperature of quarter of most solar radiation. The latter four variables were newly computed in analogy to bio8, bio9, bio16 and bio17, but relying on solar radiation to define quarters.

In addition, we calculated two variables that based on an a-priori assessment of parameters important for activity of lacertid activity: (1) Yearly hours above 30°C at 1 cm above the ground, rock surface, and at full sun exposure from the Microclim data set [60] at a spatial resolution of ~18 x 18 km). This variable was chosen based on information on preferred activity temperatures and field body temperatures of lacertids which for almost all species average above 30°C. (2) Yearly hours above 4°C and with solar radiation >100W/m². This variable is based on the fact that heliothermic lizards might be able to reach activity temperatures from solar radiation even at low air temperatures; in lacertids, a specimen of *Zootoca vivipara* has been found active at a temperatures (2 cm above ground) of 4.2°C and maximum day temperatures of 8.2°C [61] and montane *Iberolacerta* species at high elevations maintain activity at air temperatures of 9.4–9.5°C and in exceptional cases of *I. bonnali,* at 3.2 °C [62]. 100 W/m² solar radiation corresponds roughly to cloudy sky in summer.

To determine the influence of bioclimatic variables in predicting the observed species richness patterns, we performed mixed Spatial Auto-Regressive (SAR) models using the software Spatial Analysis in Macroecology [63]. Richness values were summed by binning species occurrences from the GARD distributional data set [64] into a 200 km^2 grid and counting the number of species in each cell. We aggregated the 10 arc-second bioclimate data from Worldclim 2.0 to 200 km^2 by calculating the mean values of each variable at the coarser spatial scale. For the SAR model, we input an alpha parameter of 1 based on measured geographic distances between all sites, and estimated the Rho values for each model. The CAR models were iterated until only those response variables that contributed significantly to the model were included. Then, the remaining variables were backward eliminated, starting with variables with the smallest standardized β values, until the AICc of the reduced model exceeded the more complex model [65]. Results are shown in Supplementary Table 3.

Dynamics of climatic and physiological evolution

We first evaluated the phylogenetic signal of all climatic variables by means of Blomberg's K statistic [66] using the function phylosig, available in the R package phytools [34]. While a pattern of phylogenetic signal does not allow reliable inferences about evolutionary process [67], we still consider a strong phylogenetic dependence of key niche traits as indication of phylogenetic niche conservatism [68], in particular with high values of Blomberg's K close to or above 1, as with several bioclimatic variables herein (Supplementary Table 4).

In order to remove collinearity of bioclimatic variables in downstream analyses we used the caret package in R [69] to identify the nine least-correlated variables with a threshold of $r<0.65$. We then performed a phylogenetic principal component analysis (PPCA) on these nine least-correlated variables, plus the biologically informed variable yearly hours >30°C. The PPCA was performed in the package phytools [34] using a correlation matrix. See Supplementary Table 8 for factor loadings.

Subsequently, (1) we computed a disparity-through-time plot (DTT) [70] with our data, and performed 1,000 simulations by means of a Brownian motion model (BM). We then computed

the “morphological disparity index” (MDI) statistic, which in this study it is used as a proxy of climatic disparity, defined as the area between the DTT for our empirical data and the median of the simulations. Negative MDI values indicate lower subclade disparity than expected under BM and are a common property of adaptively radiating groups [70–72]. We also calculated the p-value associated with our empirical MDI [72]. All calculations described were performed using the function dtt in the package Geiger [73]. In addition to this we (2) also calculated the absolute value of standardized independent contrasts (sic) [74] for our climatic dataset and regressed it against the ages of the nodes associated to each sic [75]. A significant positive relationship between node age and sic value is consistent with rates of climatic evolution slowing down through time, whereas a significant negative relationship between node age and sic value is consistent with an increase in the rates of climatic evolution [72,76].

In addition to this we ran a number of regression models to explore the relationship between the climatic variable, monthly hours > 30 ºC, a-priori considered as biologically relevant, and the physiological variables (T_{pref} , IWL). Preliminary analyses showed that some of the variables could potentially have non-linear relationships between them. We addressed this by fitting to each combination of variables linear and second order polynomial regression models. These were subsequently compared using their Akaike Information Criterion (AIC) values and we selected the second order polynomial regression models if these showed AIC values three or more points below the AIC of the linear models. These models were built in the R package phylolm [77]. Error terms were defined by four alternative models of evolution: a Brownian motion model (BM), an Ornstein-Uhlenbeck (OU) model with an ancestral state estimated at the root (OUfixedRoot), an OU model with the ancestral state at the root having the stationary distribution (OUrandomRoot) and a Pagel's lambda model (lambda). Model fitting was based on maximum likelihood, and we selected the OUfixedRoot model based on the output ranking of each model by the AIC value.

In our analysis of latitudinal distribution of species richness in Lacertidae, our best mixed model had an r^2 of 0.444 and an AICc of 13859.06. This model was characterized by 12 significant predictor variables, of which the following five were most influential (measured from absolute values of standardized β coefficient: a positive relation with yearly hours >4°C and >100W/m^2; a negative relation with solar radiation seasonality; a negative relation with mean solar radiation of coldest quarter; a positive relation with mean solar radiation of warmest quarter; and a negative relation with isothermality (bio3).

For the Lacertini included, our best mixed model for species richness had an r^2 of 0.349and an AICc of 10775.905. This model was characterized by 10 significant predictor variables, and mostly driven by: negative relations with hours >4°C and with the minimum temperature of the coldest week, and a positive relation with isothermality.

For the Eremiadini, our best mixed model for species richness had an r^2 of 0.531 an AICc of 12947.11. This model was characterized by 15 significant predictor variables, and mostly driven by a negative relation with annual mean temperature and a positive relation with annual mean solar radiation; and in addition, a negative relation with mean solar radiation of coldest quarter, and positive relations with mean temperature of warmest and driest quarters.

We explored the dynamics of diversification in lacertids using two complementary approaches. Firstly, 15 alternative models of diversification were fitted using the package RPANDA [78]. In these models, in six of them speciation is set to covary with time with linear and exponential functions, setting extinction to 0, to a constant value or covarying linearly with time in each case [79]. In another six models speciation is set to covary with the estimates of paleotemperature across the Cenozoic inferred from delta O^{18} measurements [80–82] (data available in the package RPANDA). Again in these models speciation is set to covary with temperature with linear and exponential functions, setting extinction to 0, to a constant value or setting it to linear covariate

with temperature [82]. Finally we also fitted three additional models in which speciation was set constant through time, with no extinction, constant extinction, or extinction covarying linearly with time [79]. We fitted these 15 models to the subfamily Lacertinae (Lacertini+Eremiadini, excluding Gallotinae), and to the clades Eremiadini and Lacertini. In all cases the most supported models implied a strong decrease in speciation rates; however we found differences between tribes regarding the covariation of these decreases in speciation rates with the estimates of paleotemperature across the Cenozoic. In Eremiadini, the most supported model imply a strong covariation with temperature, with constant extinction through time, while in Lacertini the most supported model specified an inverse covariation between speciation and extinction rates with time, with low support of models that specified covariation between speciation/extinction rates with temperature. Given these opposite trends in both clades, the diversification dynamics in Lacertinae were equally well explained by two alternative models, both high supported: one implying covariation with temperature and the other implying covariation with time. The results are shown in Supplementary Figure 11B and Supplementary Table 8-S9.

Secondly, we also estimated the dynamics of diversification by means of an alternative approach: the Bayesian Analysis of Macroevolutionary Mixtures (BAMM) [83]. Through a Markov Chain Monte Carlo, it estimates shifts in the rates of speciation and extinction among the branches of a phylogenetic tree and through time. Two runs of 30,000,000 generations each were conducted, sampling estimates every 10,000 generations. Both runs converged to similar posterior estimates and were pooled together after excluding the 10% of generation of each one as burn in. Data were then imported in the R package BAMMtools v2.1.6 for subsequent analyses. Consistent with RPANDA, BAMM detects a strong decline of diversification rates through time in the subfamily Lacertinae. This declining tendency is interrupted by a notable increase around the 30 Ma (Oligocene), coincident with the onset of diversification of Lacertini (Figure 3).

Analyses of drivers of molecular substitution rates

As a proxy for molecular substitution rates we inferred root-to-tip paths, i.e., the distance from root to tip [84], for the (non-ultrametric) 262-taxon tree from the combined analysis of RNAseq, AHE and five additional genes. Our main goal was to test if the molecular substitution rate for which root-to-tip path was used as a proxy, is predicted by the physiological key traits and bioclimatic environment of lacertid species. To exclude that the encountered patterns being influenced by generation, time (another main driver of substitution rates), we assembled maximum body sizes (snout-vent length, SVL) for all lacertid species in our analysis. Though SVL is an imperfect proxy for generation time (a variable unknown for most lacertids), it is long known that the two variables are highly correlated across animals [85] and body size-molecular rate correlations are typically mainly explained by generation time effects, besides metabolic rate effects [86]. In lacertids it is obvious that generation time will be lower in the giant lacertids such as *Timon* (SVL up to 260 mm) or *Gallotia* (SVL up to 500 mm) than in other, mostly small species such as many *Mesalina*, *Psammodromus* or *Algyroides* (max. SVL < 60 mm). For the analysis herein we extracted body size of lacertid species from [92] and adjusted the data set with original data for a few recently described species. We then performed phylogenetic multiple regressions to test whether body size, or bioclimatic or ecophysiological variables predicted root-to-tip distance (using evolutionary models already described in the previous section). SVL was log-transformed in all analyses due to its high level of skewness.

To test whether the encountered pattern of temperature-driven root-to-tip paths in the 262-taxon tree was driven by substitution rates of single genes we analyzed a subset of the phylotranscriptomic data set with sequences of the outgroup (*Blanus*) represented to ensure correct rooting. Because of missing data for varying taxa in the single-gene alignments, performing phylogenetic regressions on each of these 5,878 protein-coding genes was

computationally too complex and we therefore chose uncorrected regressions as approximation. We applied a custom script to infer gene trees from each alignment, calculate root-to-tip paths, and regress for each gene the root-to-tip paths to our hours >30°C variable. Results were tabulated and slope, coefficient and statistical significance of the correlation examined. For the seven genes that deviated from the general pattern and had significantly negative slopes, we tested for non-neutrality with Codon-based Z test of selection of overall averages in MEGA 7 [87].

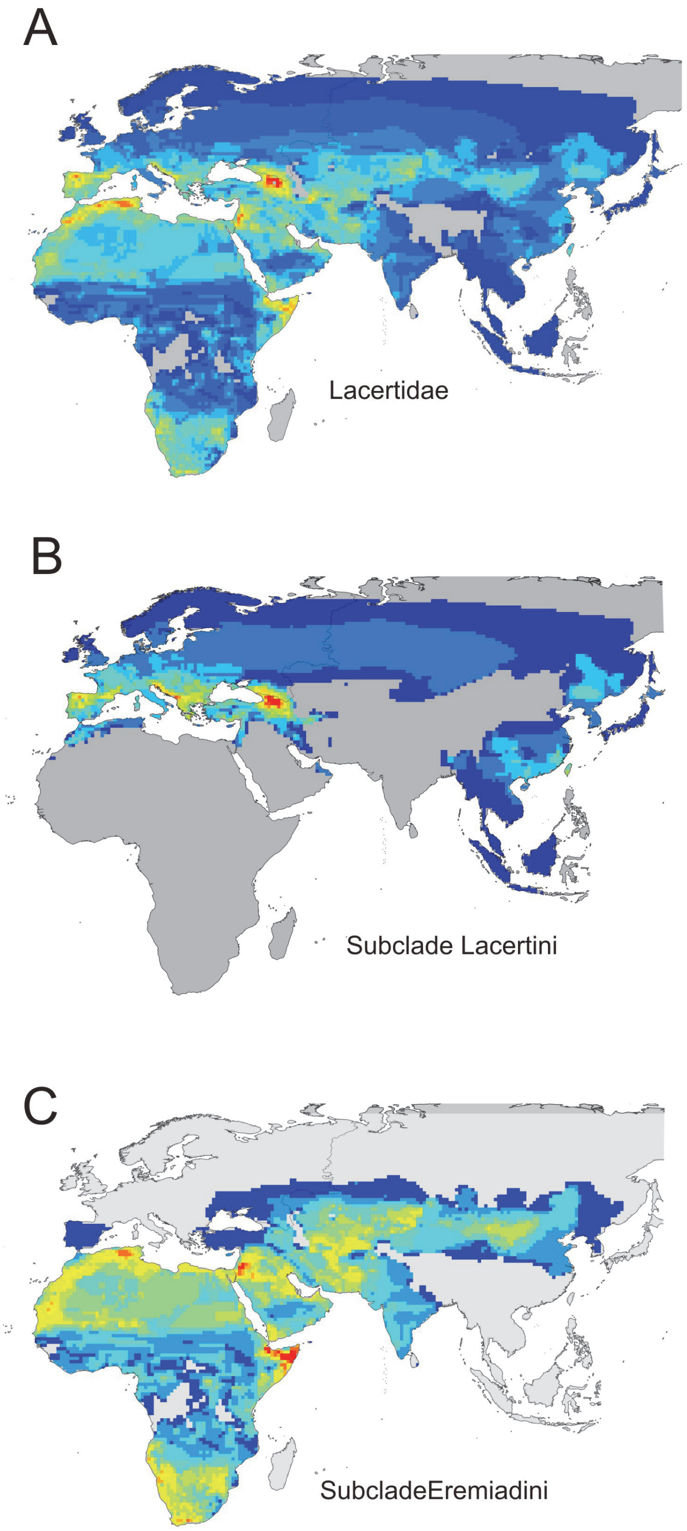

Supplementary Figure 1. Species richness of species of (A) the Lacertidae, (B) the Lacertini, and (C) the Eremiadini based on GARD distribution maps [64]. Warmer colours represent higher species richness.

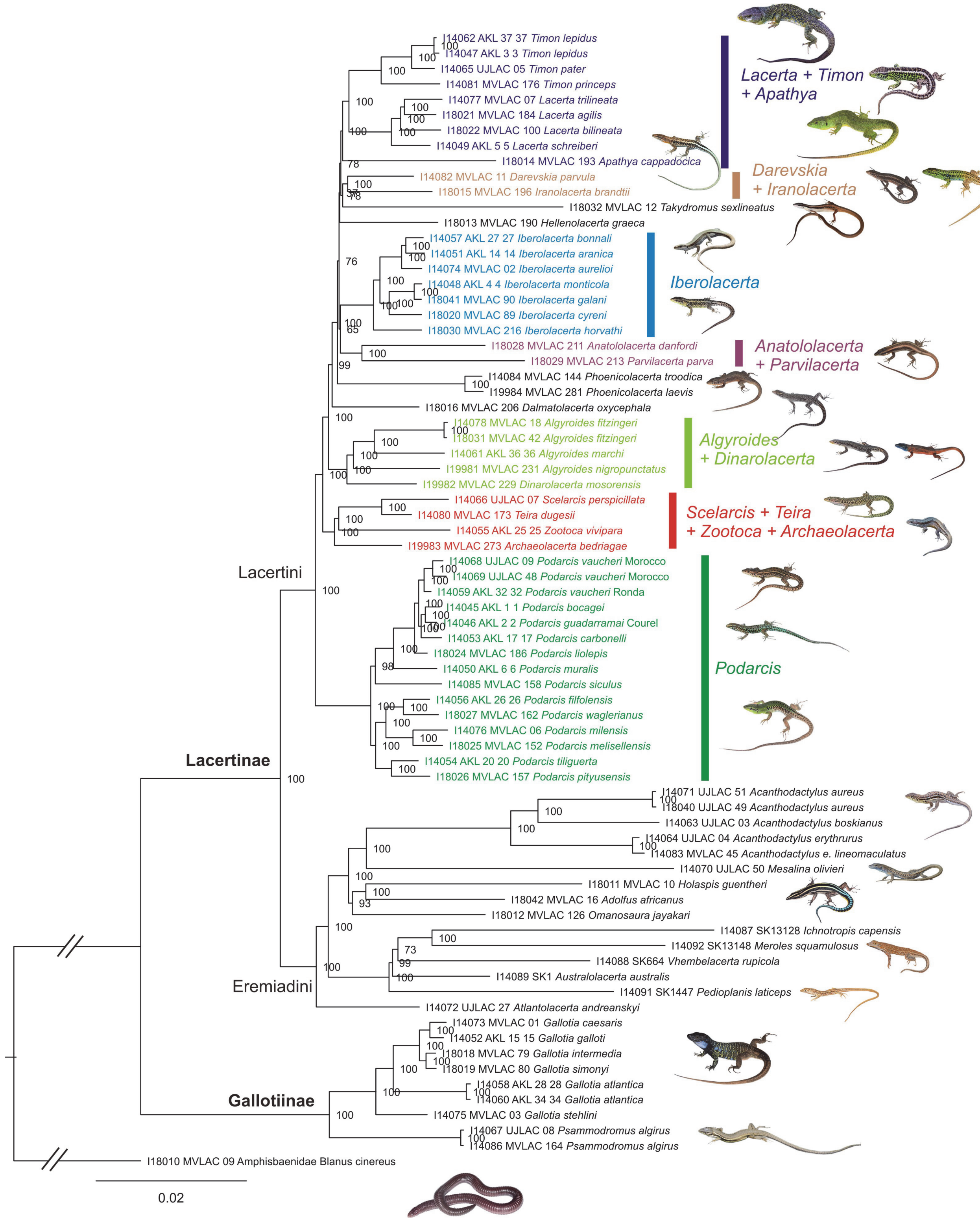

Supplementary Figure 2. Phylogenomic relationships of lacertid lizards, based on a partitioned maximum likelihood analysis of concatenated sequences of 324 anonymous nuclear loci from anchored hybrid enrichment sequencing, as given in Fig. 1B of main paper. Bootstrap values and sample numbers are indicated. Main clades recovered within Lacertini are indicated in colour.

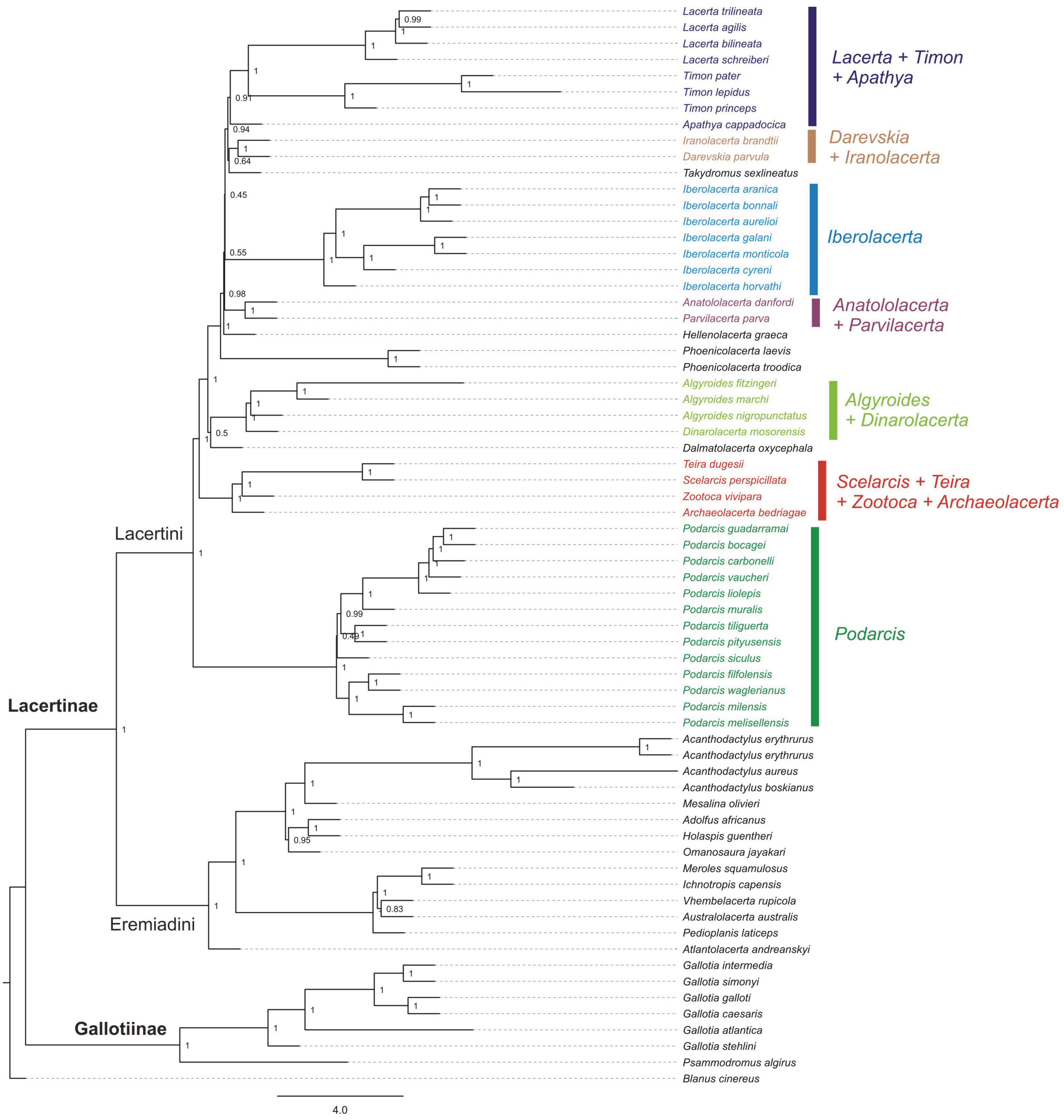

Supplementary Figure 3. Species tree of lacertid lizards, based on ASTRAL analysis of 324 anonymous nuclear loci from anchored hybrid enrichment sequencing. Main clades recovered within Lacertini are indicated in colour.

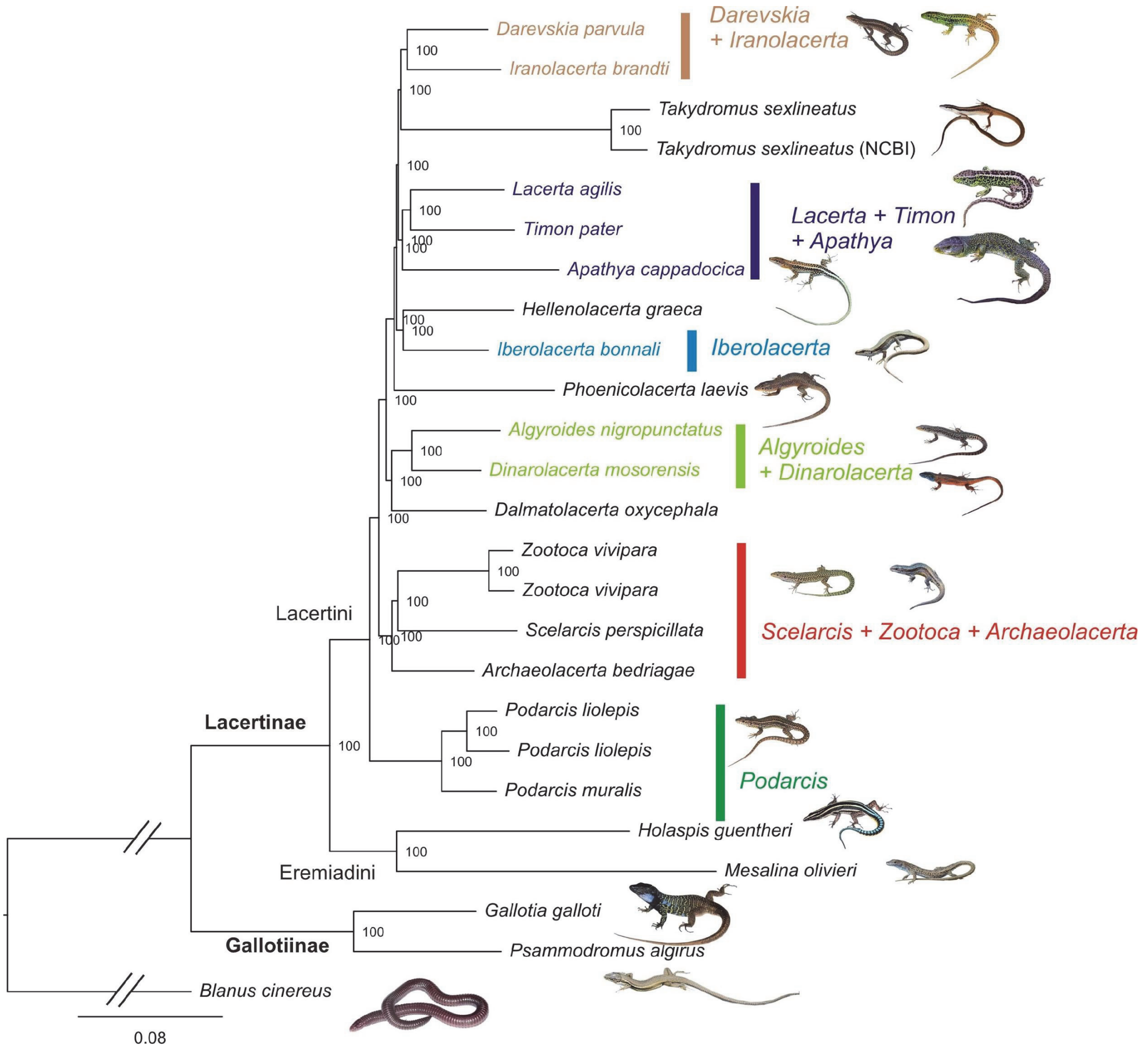

Supplementary Figure 4. Phylogenomic relationships of lacertid lizards, based on a partitioned maximum likelihood analysis of concatenated sequences of 6,269 protein-coding nuclear loci obtained by RNAseq, as in Fig. 1A of main paper. Bootstrap values and sample numbers are indicated, and outgroup is shown. Main clades recovered within Lacertini are indicated in colour.

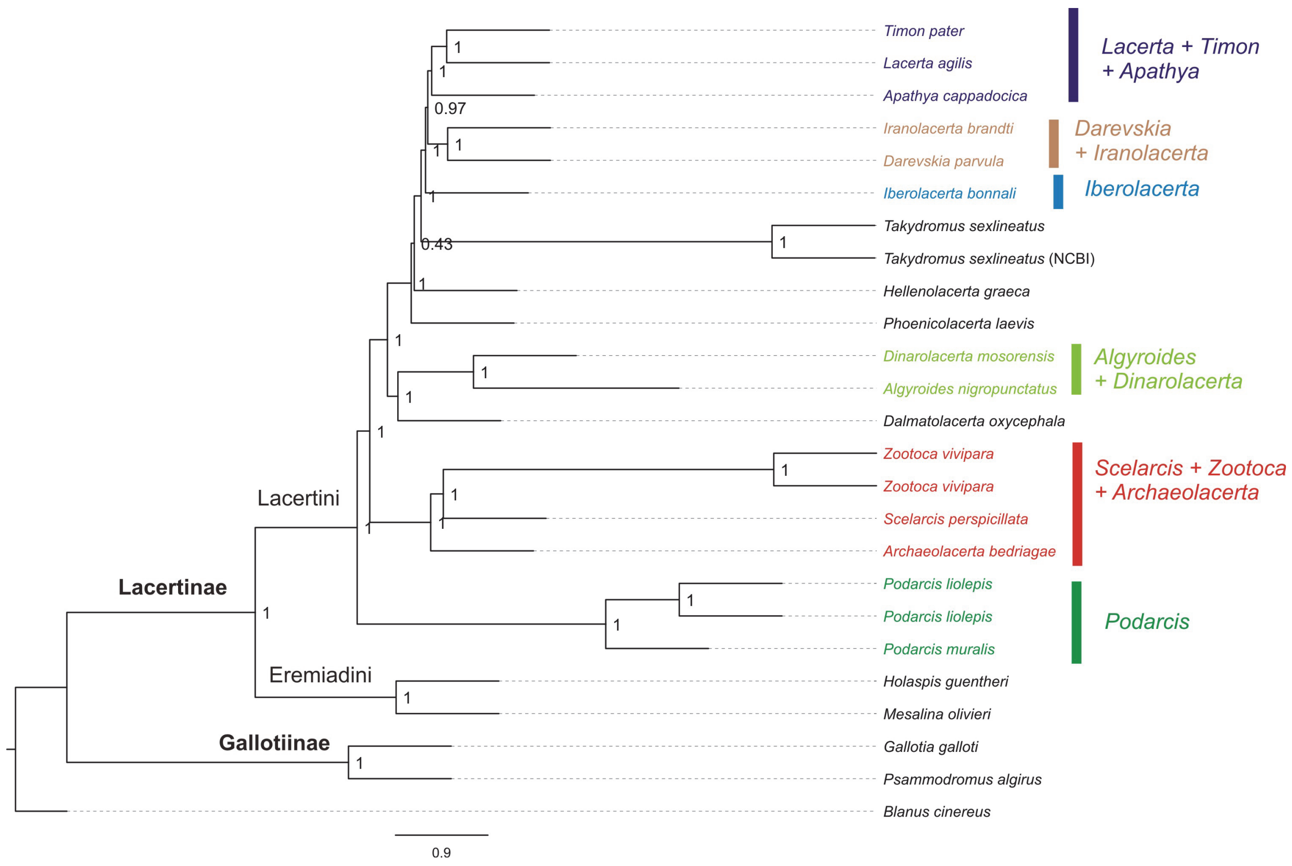

Supplementary Figure 5. Species tree of lacertid lizards, based on ASTRAL analysis of 6,269 protein-coding nuclear loci obtained by RNAseq. Main clades recovered within Lacertini are indicated in colour.

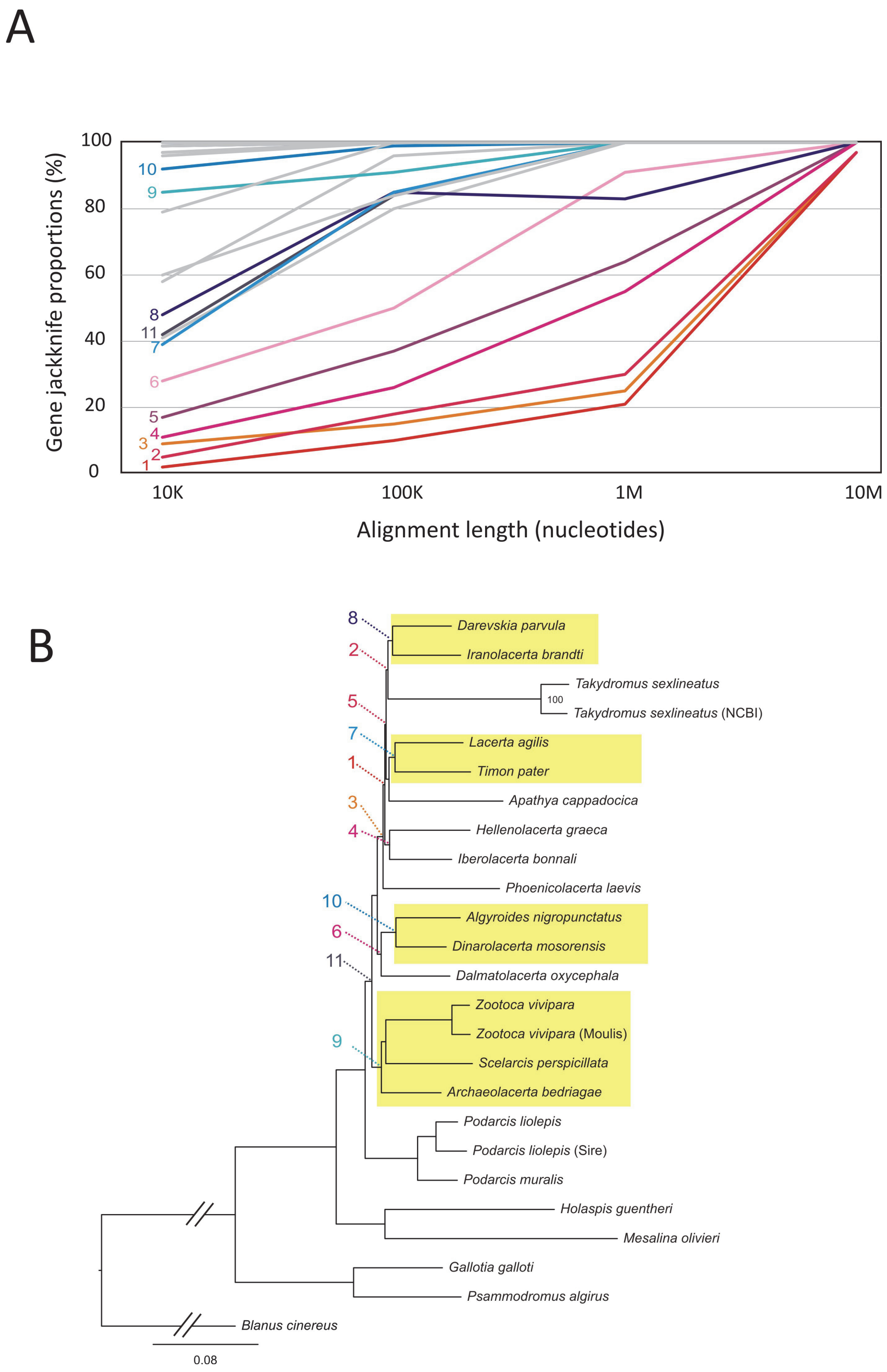

Supplementary Figure 6. Results of gene jacknifing analyses of the RNAseq data set. Panel A shows gene jackknife proportions (GJP) for different nodes in the topology of the maximum likelihood tree as reproduced in panel B. Nodes of interest are numbered in both panels, with nodes 1-6 (coloured in reddish tones) representing the six nodes that did not receive 80% GJP support with 100,000 nucleotides. Nodes 7-10 (coloured in blue tones and with taxa highlighted in yellow) define highly supported clades newly recovered in this study; all of these received 80% GJP with 100,000 nucleotides and all except node 8 received 100% GJP with 1 million nucleotides.

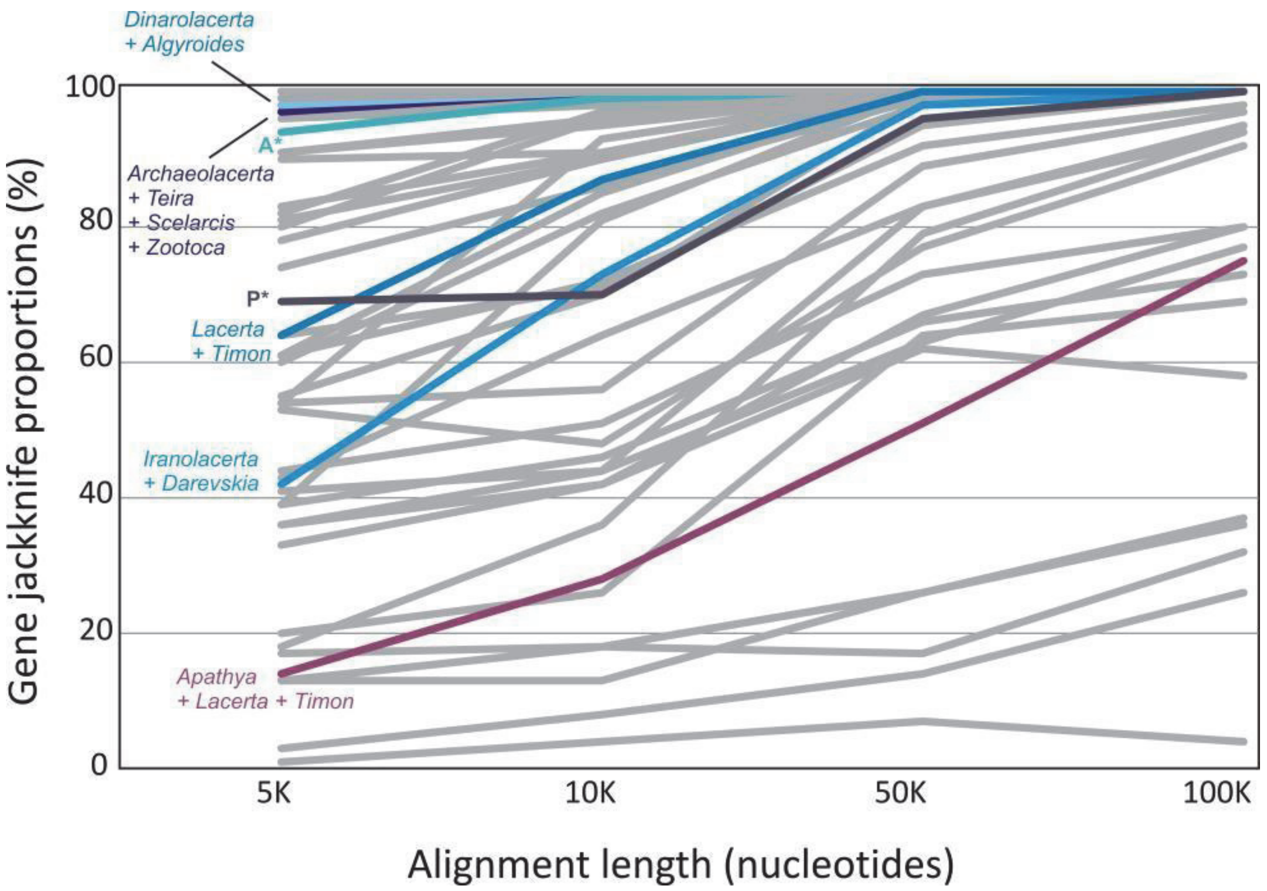

Supplementary Figure 7. Results of gene jacknifing analyses of the AHE data set, with gene jackknife proportions (GJP) for different nodes in the topology of the maximum likelihood tree as shown in Figs. 1 and S1. The coloured lines represent values for nodes defining clades newly recovered in this study; P* marks the line for the placement of *Podarcis* sister to all other Lacertini; A* marks the placement of *Atlantolacerta* sister to all other Eremiadini. All of these except for *Apathya* + *Lacerta* + *Timon* received 60% GJP with 10,000 nucleotides, and 80% GJP with 50,000 nucleotides.

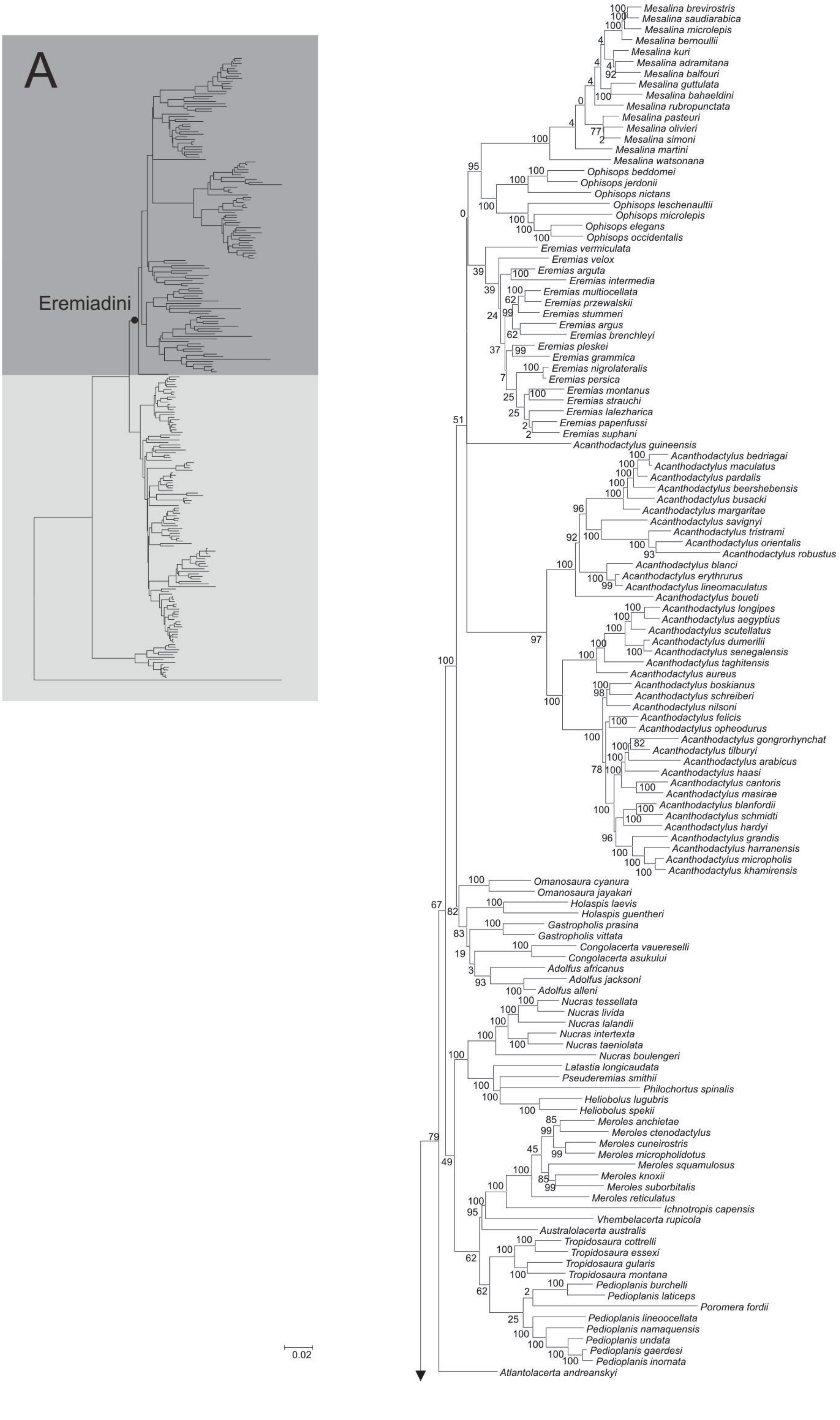
A
Eremiadini
0.02
Mesalina brevirostris
Mesalina saudiarabica
Mesalina microlepis
Mesalina bernoullii
Mesalina kuri
Mesalina adramitana
Mesalina balfouri
Mesalina guttulata
Mesalina bahaeldini
Mesalina rubropunctata
Mesalina pasteuri
Mesalina olivieri
Mesalina simoni
Mesalina martini
Mesalina watsonana
Ophisops beddomei
Ophisops jerdonii
Ophisops nictans
Ophisops leschenaultii
Ophisops microlepis
Ophisops elegans
Ophisops occidentalis
Eremias vermiculata
Eremias velox
Eremias arguta
Eremias intermedia
Eremias multiocellata
Eremias przewalskii
Eremias stummeri
Eremias argus
Eremias brenchleyi
Eremias pleskei
Eremias grammica
Eremias nigrolateralis
Eremias persica
Eremias montanus
Eremias strauchi
Eremias lalezharica
Eremias papenfussi
Eremias suphani
Acanthodactylus guineensis
Acanthodactylus bedriagai
Acanthodactylus maculatus
Acanthodactylus pardalis
Acanthodactylus beershebensis
Acanthodactylus busacki
Acanthodactylus margaritae
Acanthodactylus savignyi
Acanthodactylus tristrami
Acanthodactylus orientalis
Acanthodactylus robustus
Acanthodactylus blanci
Acanthodactylus erythrurus
Acanthodactylus lineomaculatus
Acanthodactylus boueti
Acanthodactylus longipes
Acanthodactylus aegyptius
Acanthodactylus scutellatus
Acanthodactylus dumerilii
Acanthodactylus senegalensis
Acanthodactylus taghitensis
Acanthodactylus aureus
Acanthodactylus boskianus
Acanthodactylus schreiberi
Acanthodactylus nilsoni
Acanthodactylus felicis
Acanthodactylus opheodurus
Acanthodactylus gongrorhynchat
Acanthodactylus tilburyi
Acanthodactylus arabicus
Acanthodactylus haasi
Acanthodactylus cantoris
Acanthodactylus masirae
Acanthodactylus blanfordii
Acanthodactylus schmidti
Acanthodactylus hardyi
Acanthodactylus grandis
Acanthodactylus harranensis
Acanthodactylus micropholis
Acanthodactylus khamirensis
Omanosaura cyanura
Omanosaura jayakari
Holaspis laevis
Holaspis guentheri
Gastropholis prasina
Gastropholis vittata
Congolacerta vauereselli
Congolacerta asukului
Adolfus africanus
Adolfus jacksoni
Adolfus alleni
Nucras tessellata
Nucras livida
Nucras lalandii
Nucras intertexta
Nucras taeniolata
Nucras boulengeri
Latastia longicaudata
Pseuderemias smithii
Philochortus spinalis
Heliobolus lugubris
Heliobolus spekii
Meroles anchietae
Meroles ctenodactylus
Meroles cuneirostris
Meroles micropholidotus
Meroles squamulosus
Meroles knoxii
Meroles suborbitalis
Meroles reticulatus
Ichnotropis capensis
Vhembelacerta rupicola
Australolacerta australis
Tropidosaura cottrelli
Tropidosaura essexi
Tropidosaura gularis
Tropidosaura montana
Pedioplanis burchelli
Pedioplanis laticeps
Poromera fordii
Pedioplanis lineoocellata
Pedioplanis namaquensis
Pedioplanis undata
Pedioplanis gaerdesi
Pedioplanis inornata
Atlantolacerta andreanskyi

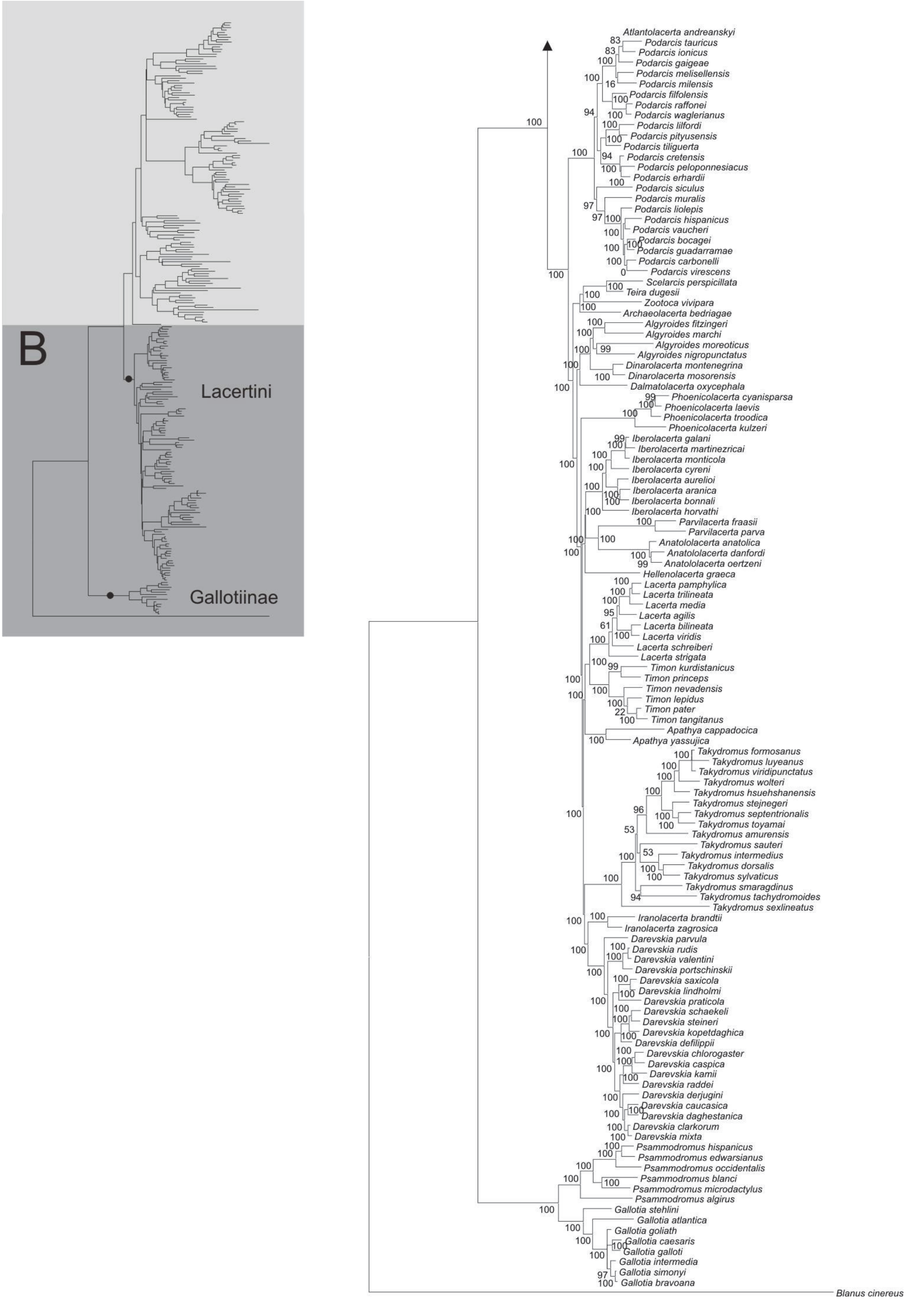

Supplementary Figure 8. Maximum likelihood tree inferred from the combined matrix of AHE, RNAseq, and five additional genes for 262 species of lacertid lizards. Numbers at nodes are support values in percent from an ultrafast bootstrap analysis (1,000 replicates). Note that in the Eremiadini, most species are only represented by 1-5 genes and many relationships are in need of confirmation.

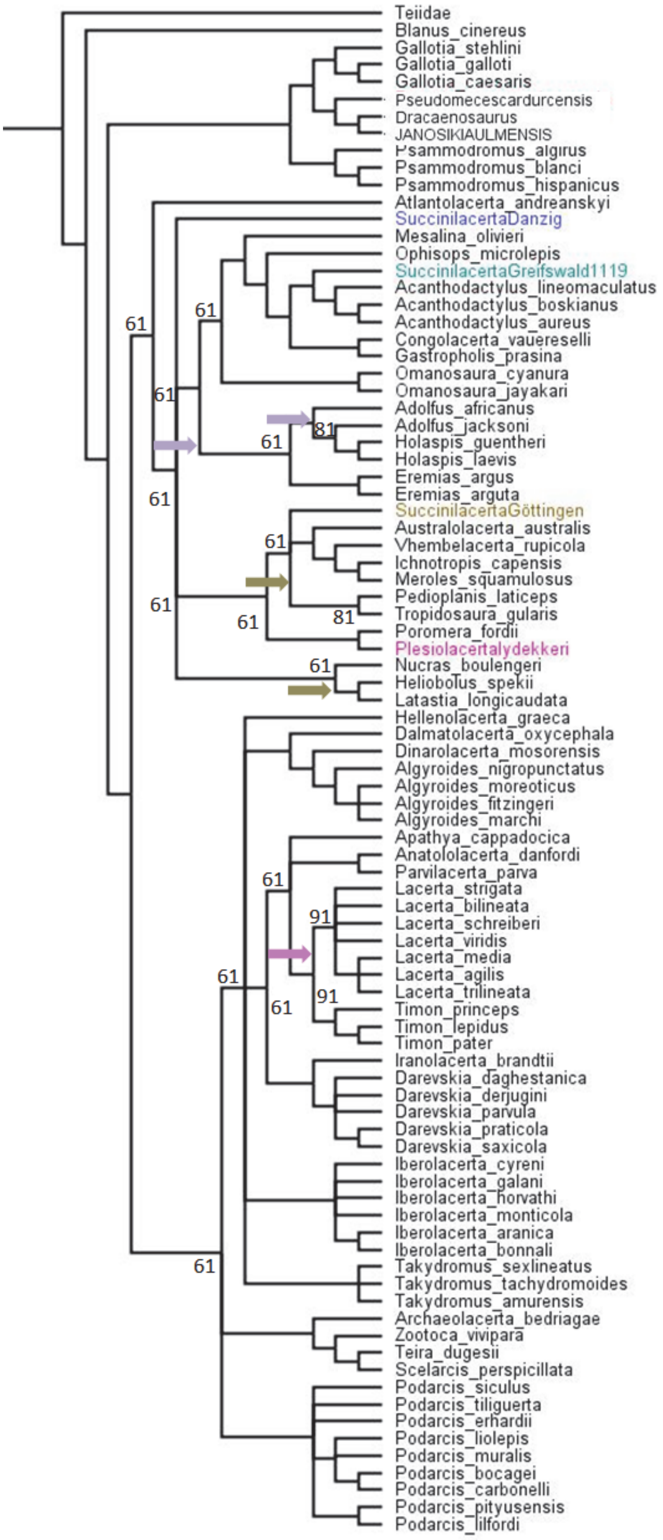

Supplementary Figure 9. Consensus tree (50% majority rule) of the 11 trees from the new technology (maximum parsimony) search combining molecular and morphological data. The data sets included also fossil lacertids, and their (partially alternative) positions recovered in the trees are marked by arrows of similar colour as the respective taxa. All unlabeled dichotomies were found in all of the trees.

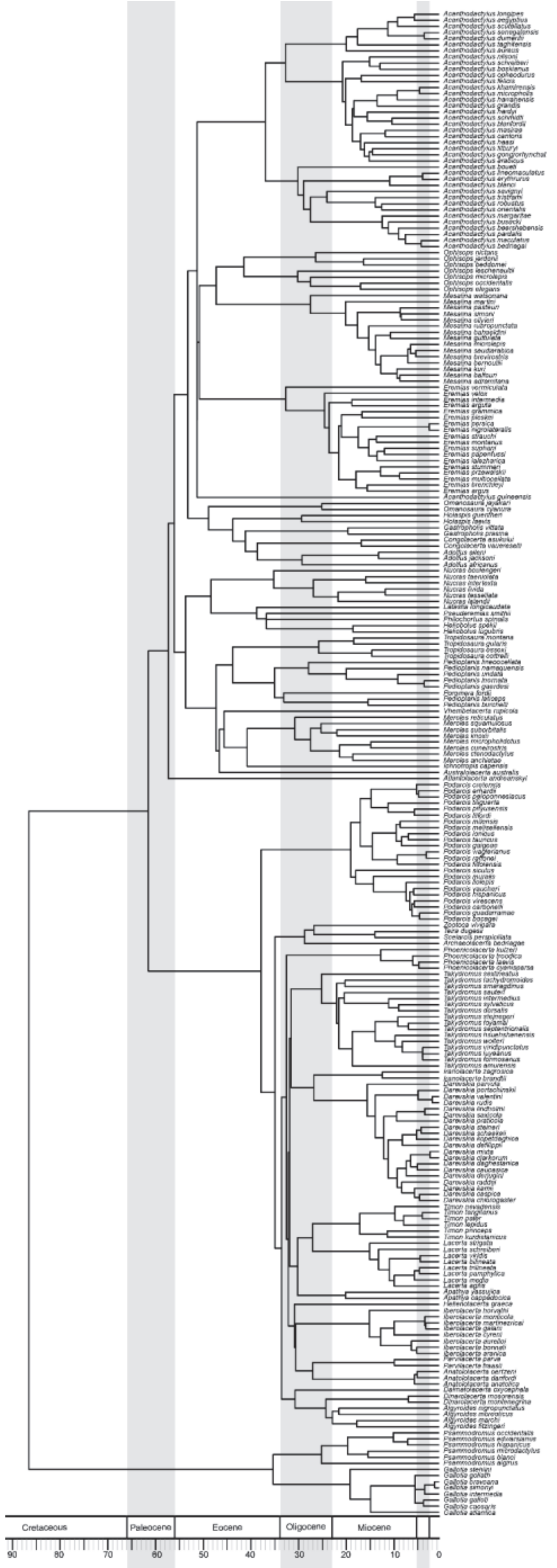

Supplementary Figure 10. Molecular timetree for Lacertidae – overview with geological periods. Scale bar in millions of years before present. Shaded bars mark Paleocene and Oligocene periods.

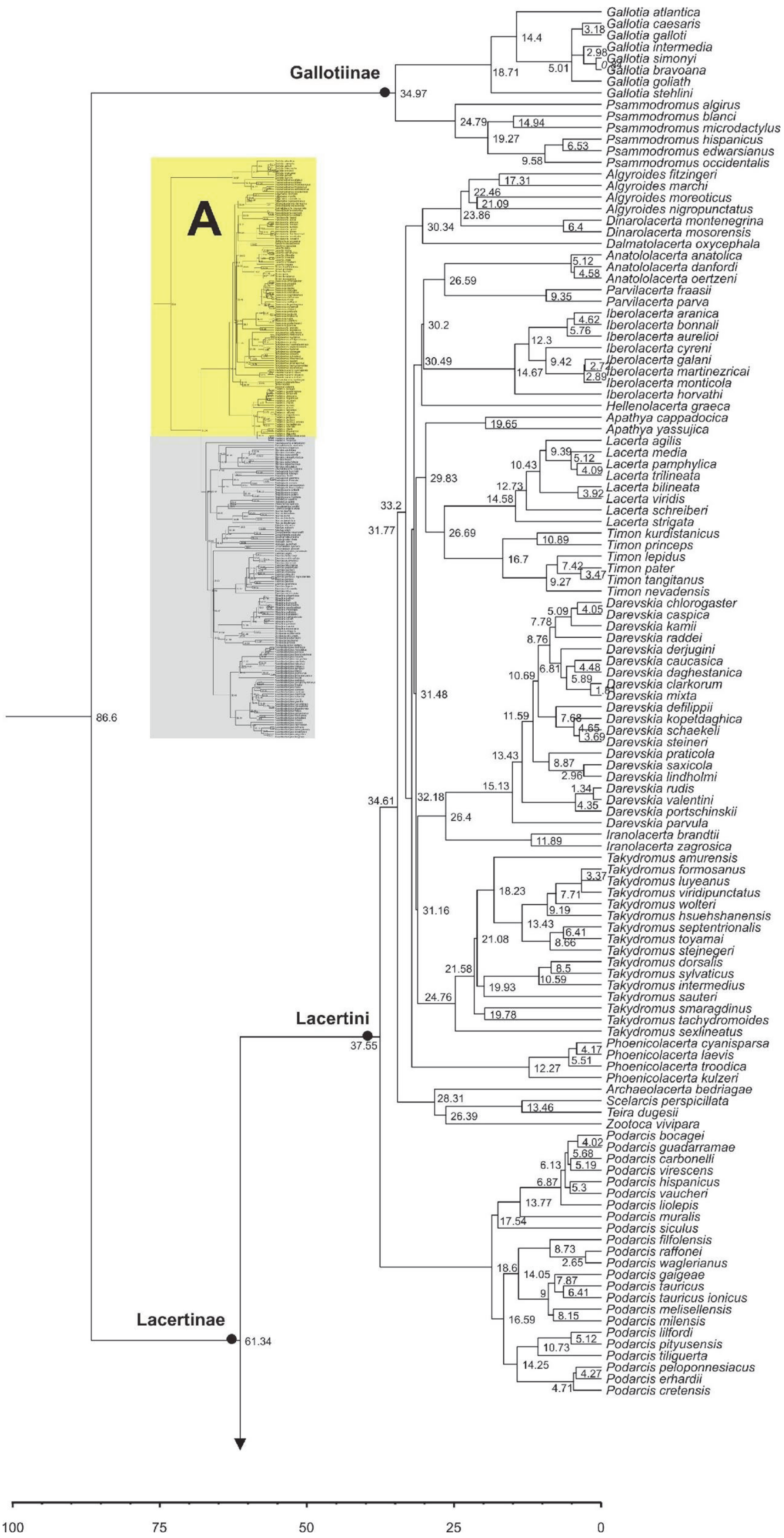
Gallotiinae
Lacertini
Lacertinae
A
86.6
34.97
37.55
61.34
Gallotia atlantica
Gallotia caesaris
Gallotia galloti
Gallotia intermedia
Gallotia simonyi
Gallotia bravoana
Gallotia goliath
Gallotia stehlini
Psammodromus algirus
Psammodromus blanci
Psammodromus microdactylus
Psammodromus hispanicus
Psammodromus edwarsianus
Psammodromus occidentalis
Algyroides fitzingeri
Algyroides marchi
Algyroides moreoticus
Algyroides nigropunctatus
Dinarolacerta montenegrina
Dinarolacerta mosorensis
Dalmatolacerta oxycephala
Anatololacerta anatolica
Anatololacerta danfordi
Anatololacerta oertzeni
Parvilacerta fraasii
Parvilacerta parva
Iberolacerta aranica
Iberolacerta bonnali
Iberolacerta aurelioi
Iberolacerta cyreni
Iberolacerta galani
Iberolacerta martinezricai
Iberolacerta monticola
Iberolacerta horvathi
Hellenolacerta graeca
Apathya cappadocica
Apathya yassujica
Lacerta agilis
Lacerta media
Lacerta pamphylica
Lacerta trilineata
Lacerta bilineata
Lacerta viridis
Lacerta schreiberi
Lacerta strigata
Timon kurdistanicus
Timon princeps
Timon lepidus
Timon pater
Timon tangitanus
Timon nevadensis
Darevskia chlorogaster
Darevskia caspica
Darevskia kamii
Darevskia raddei
Darevskia derjugini
Darevskia caucasica
Darevskia daghestanica
Darevskia clarkorum
Darevskia mixta
Darevskia defilippii
Darevskia kopetdaghica
Darevskia schaekeli
Darevskia steineri
Darevskia praticola
Darevskia saxicola
Darevskia lindholmi
Darevskia rudis
Darevskia valentini
Darevskia portschinskii
Darevskia parvula
Iranolacerta brandtii
Iranolacerta zagrosica
Takydromus amurensis
Takydromus formosanus
Takydromus luyeanus
Takydromus viridipunctatus
Takydromus wolteri
Takydromus hsuehshanensis
Takydromus septentrionalis
Takydromus toyamai
Takydromus stejnegeri
Takydromus dorsalis
Takydromus sylvaticus
Takydromus intermedius
Takydromus sauteri
Takydromus smaragdinus
Takydromus tachydromoides
Takydromus sexlineatus
Phoenicolacerta cyanisparsa
Phoenicolacerta laevis
Phoenicolacerta troodica
Phoenicolacerta kulzeri
Archaeolacerta bedriagae
Scelarcis perspicillata
Teira dugesii
Zootoca vivipara
Podarcis bocagei
Podarcis guadarramae
Podarcis carbonelli
Podarcis virescens
Podarcis hispanicus
Podarcis vaucheri
Podarcis liolepis
Podarcis muralis
Podarcis siculus
Podarcis filfolensis
Podarcis raffonei
Podarcis waglerianus
Podarcis gaigeae
Podarcis tauricus
Podarcis tauricus ionicus
Podarcis melisellensis
Podarcis milensis
Podarcis lilfordi
Podarcis pityusensis
Podarcis tiliguerta
Podarcis peloponnesiacus
Podarcis erhardii
Podarcis cretensis
100
75
50
25
0

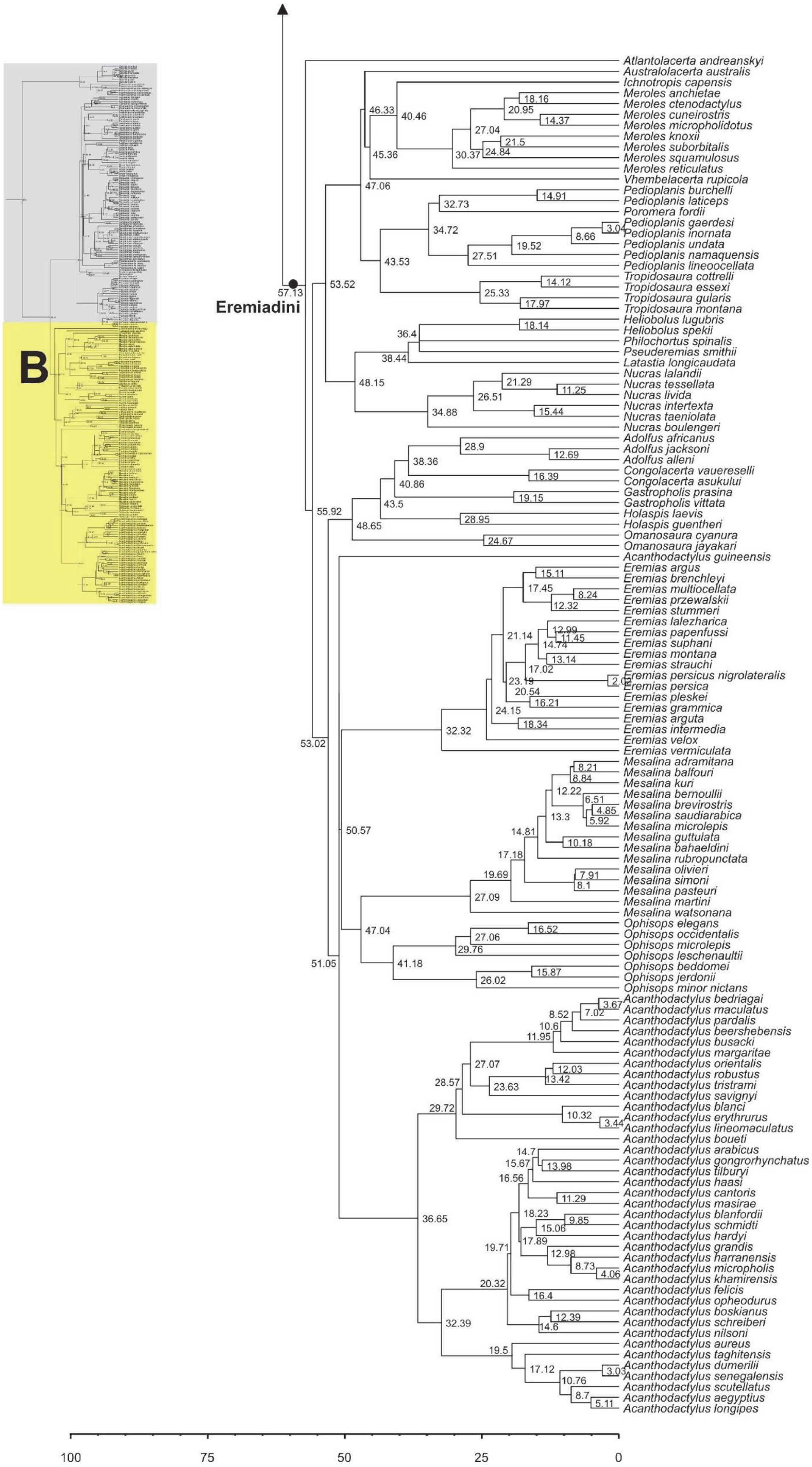

Supplementary Figure 11. Molecular timetree for Lacertidae, detailed view. Scale bar in millions of years. Values are estimated node ages in millions of years before present. Note that the penalized likelihood approach used does not estimate confidence intervals for the time estimates.

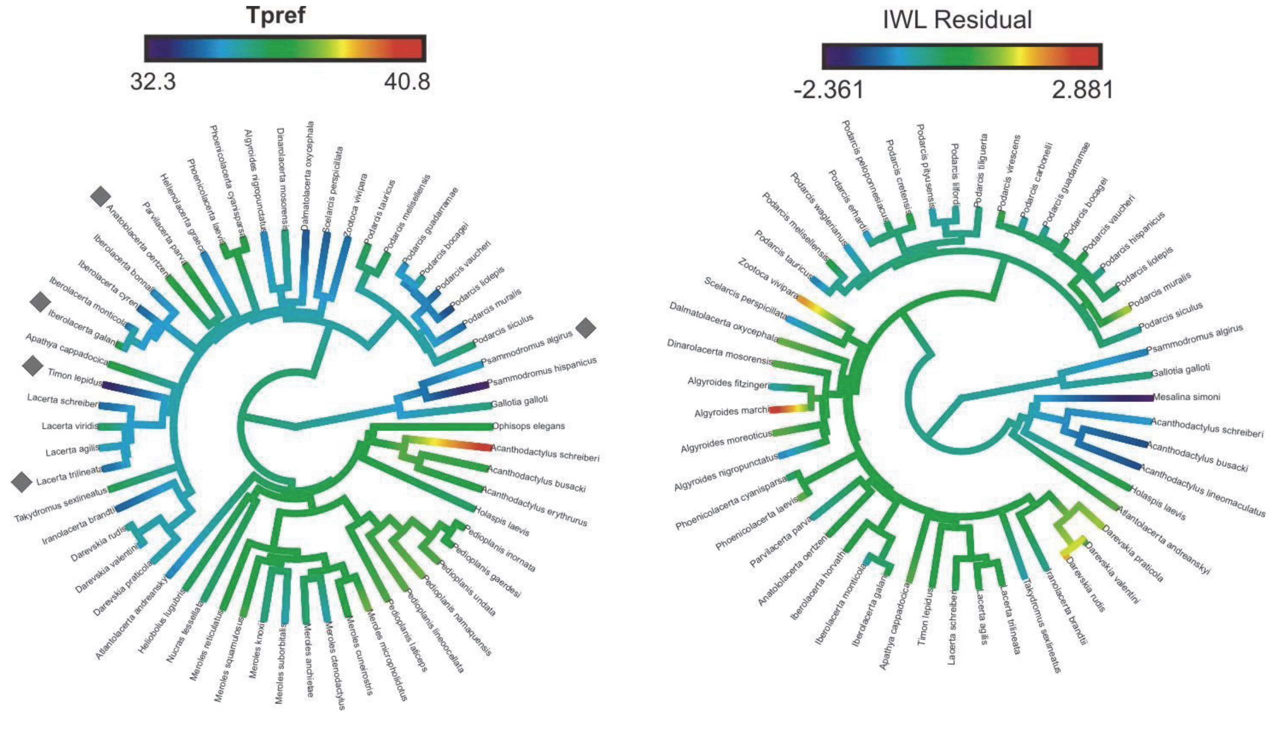

Supplementary Figure 12. Character state reconstructions for IWL and T_{pref}. Grey rhomboids mark species for which T_{pref} estimates for females were included due to missing or few male estimates.

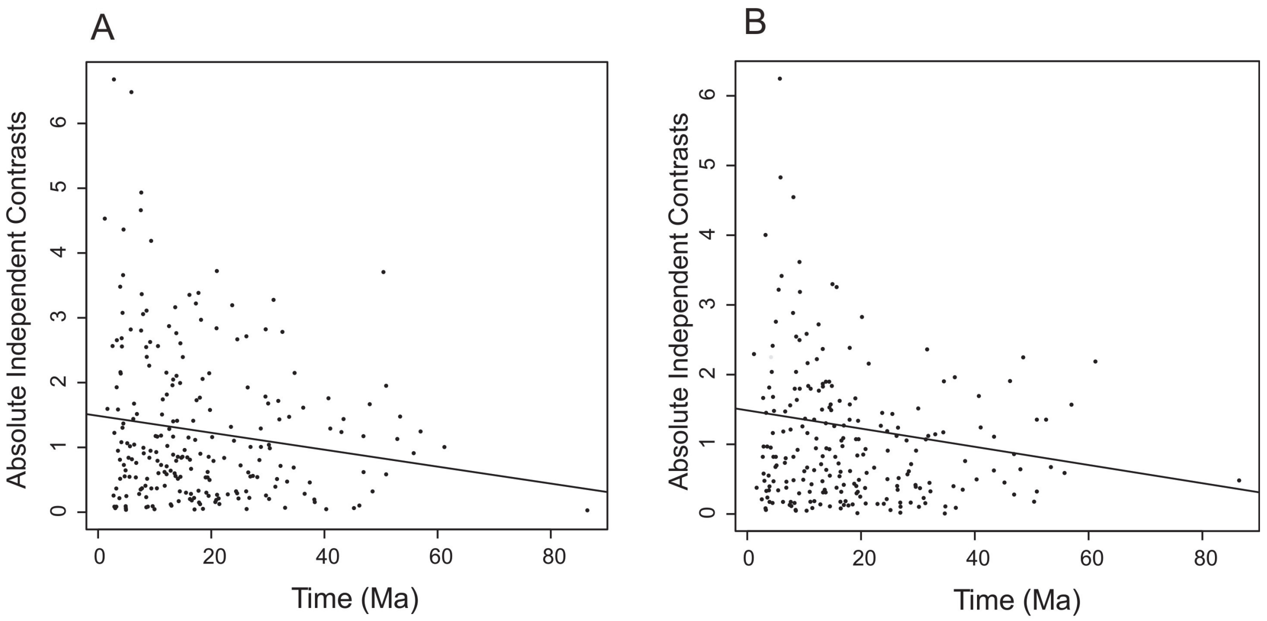

Supplementary Figure 13. Plot of absolute climate contrasts against node heights for (A) the first and (B) the second principal component (PC1 and PC2) of a phylogenetic PCA in lacertids (P = 0.02 in both analyses). Source data are provided as a Source Data file.

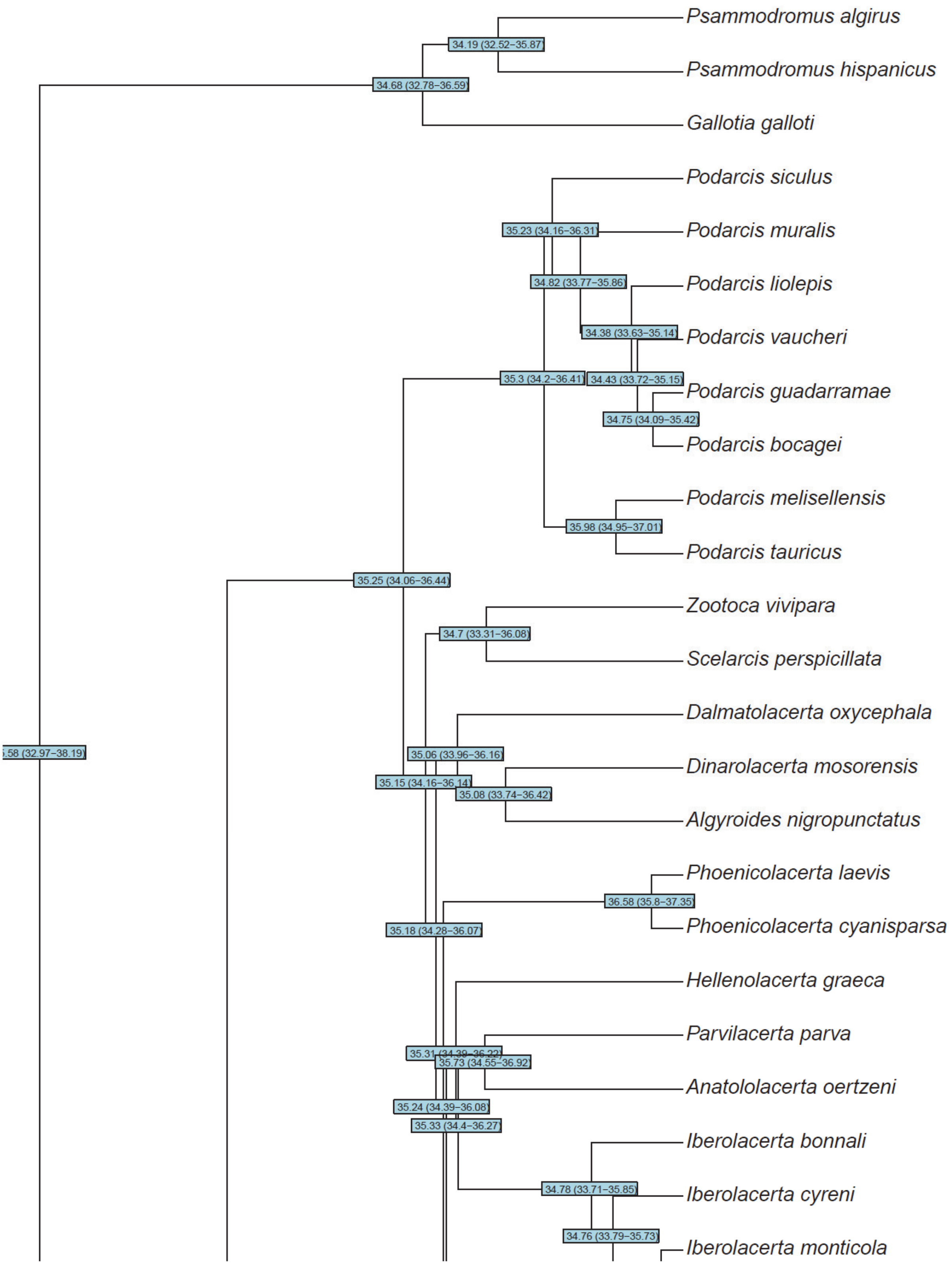
Psammodromus algirus
34.19 (32.52–35.87)
Psammodromus hispanicus
34.68 (32.78–36.59)
Gallotia galloti
Podarcis siculus
35.23 (34.16–36.31)
Podarcis muralis
34.82 (33.77–35.86)
Podarcis liolepis
34.38 (33.63–35.14)
Podarcis vaucheri
35.3 (34.2–36.41)
34.43 (33.72–35.15)
Podarcis guadarramae
34.75 (34.09–35.42)
Podarcis bocagei
Podarcis melisellensis
35.98 (34.95–37.01)
Podarcis tauricus
35.25 (34.06–36.44)
Zootoca vivipara
34.7 (33.31–36.08)
Scelarcis perspicillata
Dalmatolacerta oxycephala
.58 (32.97–38.19)
35.06 (33.96–36.16)
Dinarolacerta mosorensis
35.15 (34.16–36.14)
35.08 (33.74–36.42)
Algyroides nigropunctatus
Phoenicolacerta laevis
36.58 (35.8–37.35)
35.18 (34.28–36.07)
Phoenicolacerta cyanisparsa
Hellenolacerta graeca
Parvilacerta parva
35.31 (34.39–36.22)
35.73 (34.55–36.92)
Anatololacerta oertzeni
35.24 (34.39–36.08)
35.33 (34.4–36.27)
Iberolacerta bonnali
34.78 (33.71–35.85)
Iberolacerta cyreni
34.76 (33.79–35.73)
Iberolacerta monticola

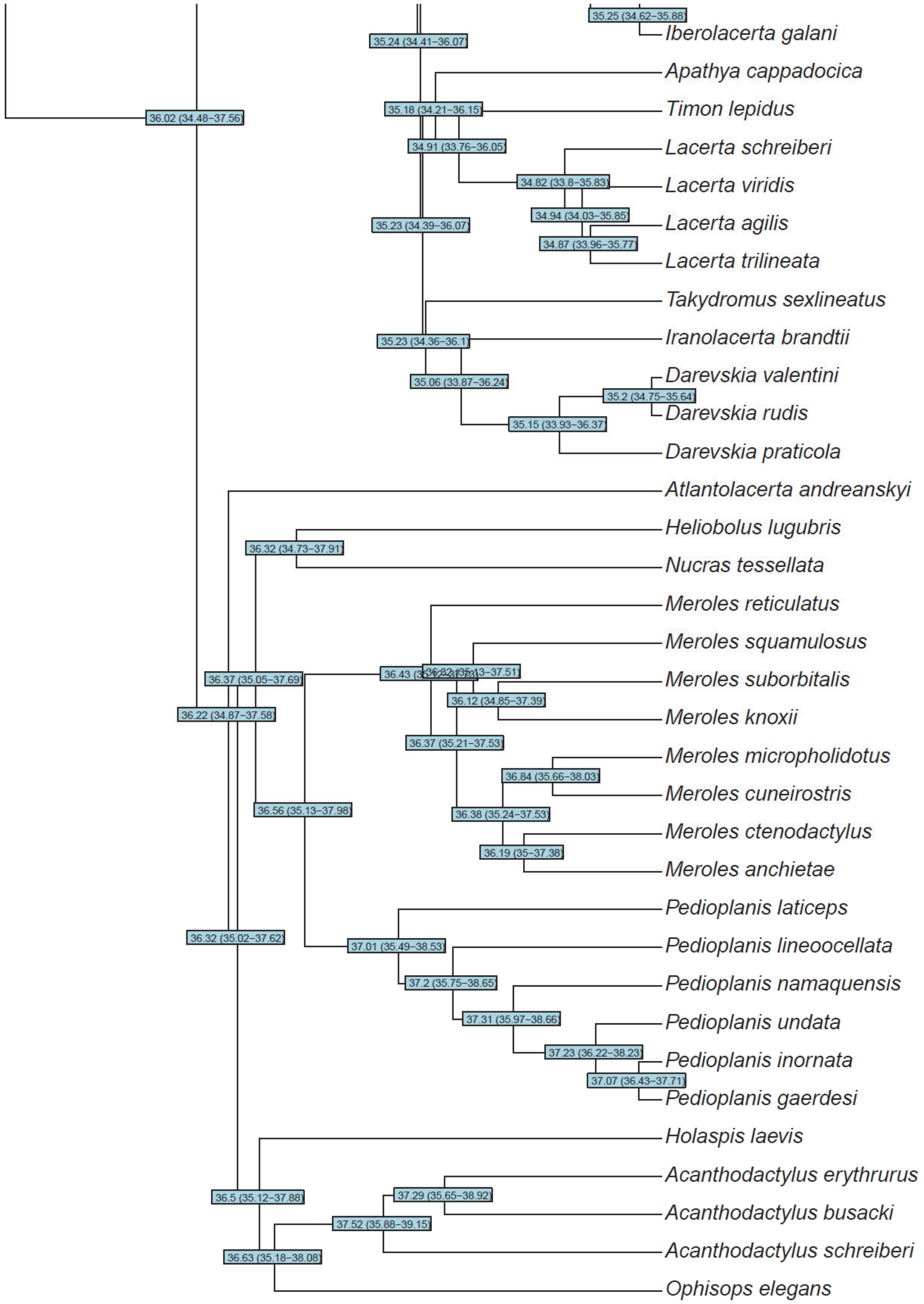

Supplementary Figure 14. Results of ancestral character state reconstructions for T_{pref} as visualized in Fig. S12 (confidence intervals in parentheses).

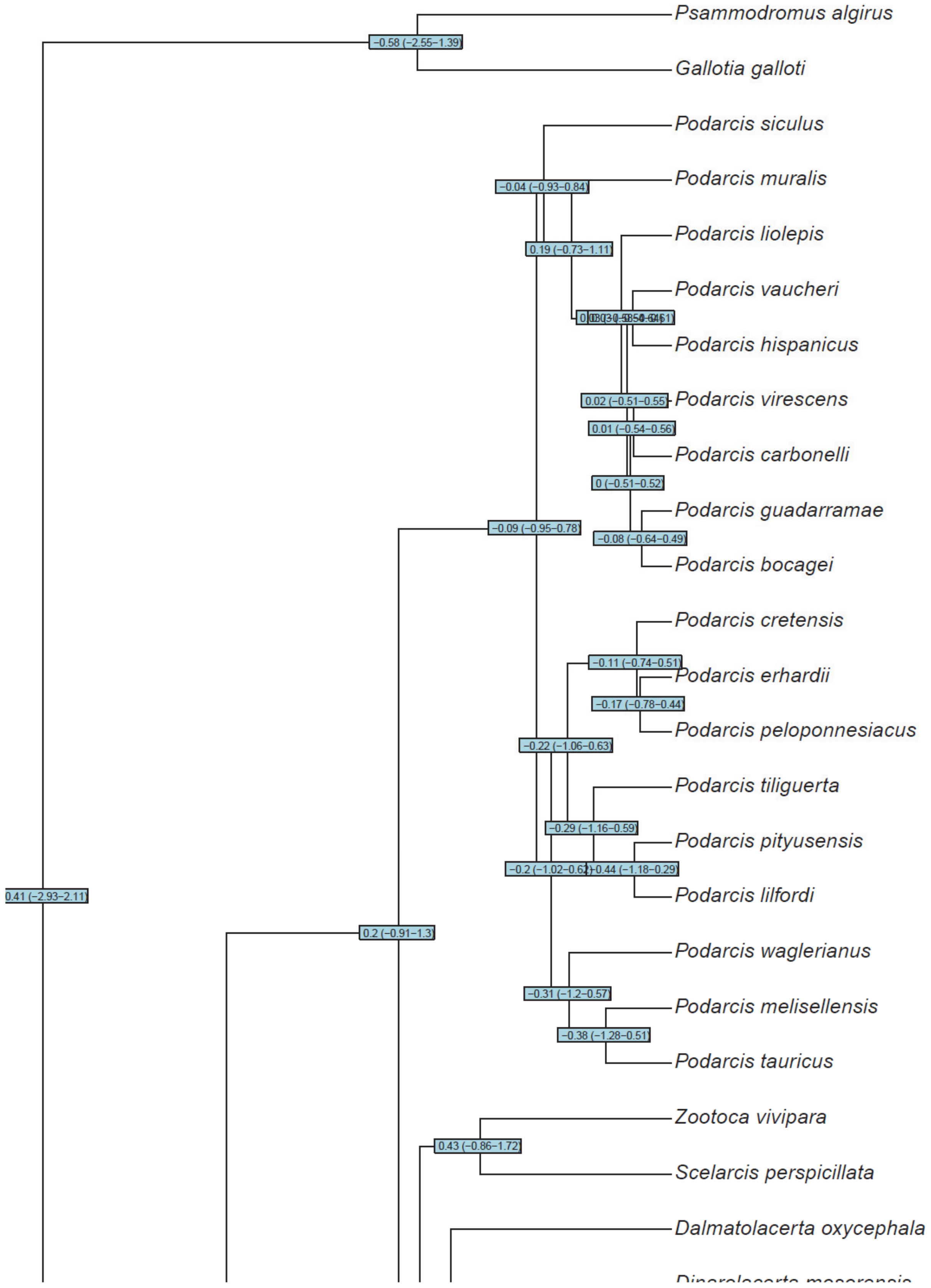
Psammodromus algirus
Gallotia galloti
Podarcis siculus
Podarcis muralis
Podarcis liolepis
Podarcis vaucheri
Podarcis hispanicus
Podarcis virescens
Podarcis carbonelli
Podarcis guadarramae
Podarcis bocagei
Podarcis cretensis
Podarcis erhardii
Podarcis peloponnesiacus
Podarcis tiliguerta
Podarcis pityusensis
Podarcis lilfordi
Podarcis waglerianus
Podarcis melisellensis
Podarcis tauricus
Zootoca vivipara
Scelarcis perspicillata
Dalmatolacerta oxycephala
−0.58 (−2.55−1.39)
−0.04 (−0.93−0.84)
0.19 (−0.73−1.11)
0.02 (−0.51−0.55)
0.01 (−0.54−0.56)
0 (−0.51−0.52)
−0.08 (−0.64−0.49)
−0.09 (−0.95−0.78)
−0.11 (−0.74−0.51)
−0.17 (−0.78−0.44)
−0.22 (−1.06−0.63)
−0.29 (−1.16−0.59)
−0.2 (−1.02−0.62)
−0.44 (−1.18−0.29)
0.41 (−2.93−2.11)
0.2 (−0.91−1.3)
−0.31 (−1.2−0.57)
−0.38 (−1.28−0.51)
0.43 (−0.86−1.72)

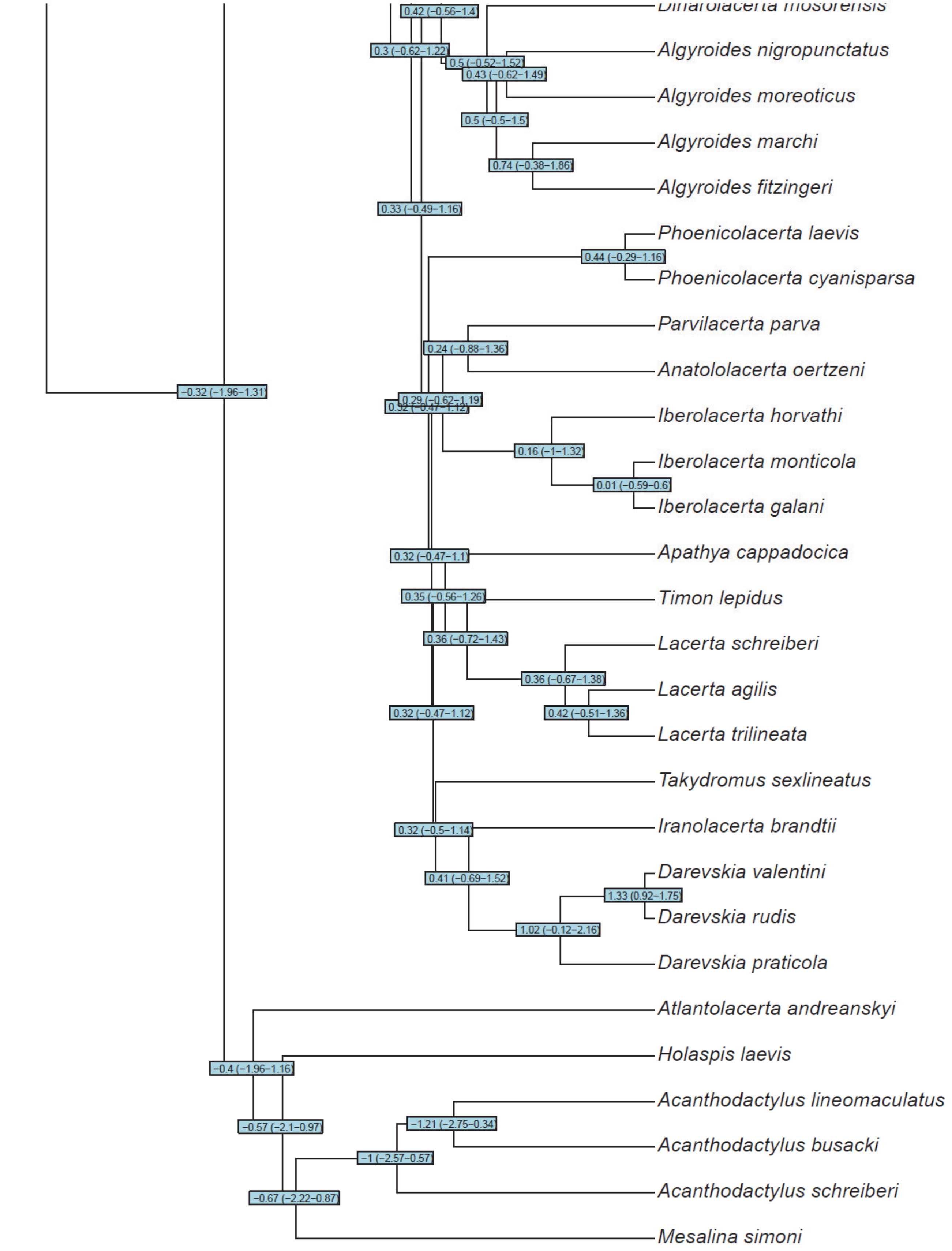

Supplementary Figure 15. Results of ancestral character state reconstructions for IWL (as visualized in Fig. S12; note that values represent regression residuals; confidence intervals in parentheses).

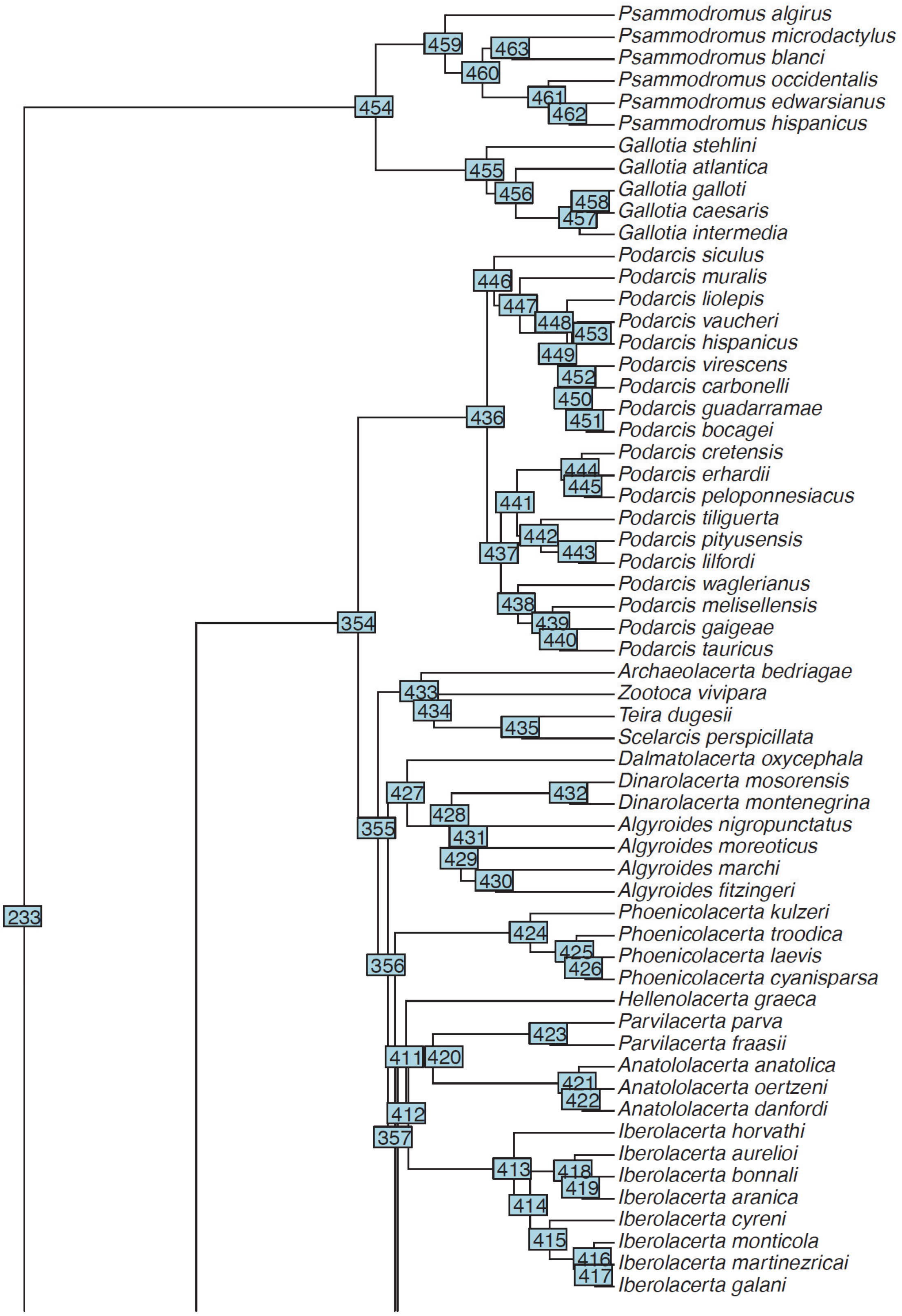
Psammodromus algirus
Psammodromus microdactylus
Psammodromus blanci
Psammodromus occidentalis
Psammodromus edwarsianus
Psammodromus hispanicus
Gallotia stehlini
Gallotia atlantica
Gallotia galloti
Gallotia caesaris
Gallotia intermedia
Podarcis siculus
Podarcis muralis
Podarcis liolepis
Podarcis vaucheri
Podarcis hispanicus
Podarcis virescens
Podarcis carbonelli
Podarcis guadarramae
Podarcis bocagei
Podarcis cretensis
Podarcis erhardii
Podarcis peloponnesiacus
Podarcis tiliguerta
Podarcis pityusensis
Podarcis lilfordi
Podarcis waglerianus
Podarcis melisellensis
Podarcis gaigeae
Podarcis tauricus
Archaeolacerta bedriagae
Zootoca vivipara
Teira dugesii
Scelarcis perspicillata
Dalmatolacerta oxycephala
Dinarolacerta mosorensis
Dinarolacerta montenegrina
Algyroides nigropunctatus
Algyroides moreoticus
Algyroides marchi
Algyroides fitzingeri
Phoenicolacerta kulzeri
Phoenicolacerta troodica
Phoenicolacerta laevis
Phoenicolacerta cyanisparsa
Hellenolacerta graeca
Parvilacerta parva
Parvilacerta fraasii
Anatololacerta anatolica
Anatololacerta oertzeni
Anatololacerta danfordi
Iberolacerta horvathi
Iberolacerta aurelioi
Iberolacerta bonnali
Iberolacerta aranica
Iberolacerta cyreni
Iberolacerta monticola
Iberolacerta martinezricai
Iberolacerta galani
454
459
463
460
461
462
455
456
458
457
446
447
448
453
449
452
450
451
436
444
445
441
442
443
437
438
439
440
354
233
433
434
435
427
432
428
431
429
430
355
424
425
426
356
411
420
423
421
422
412
357
413
418
419
414
415
416
417

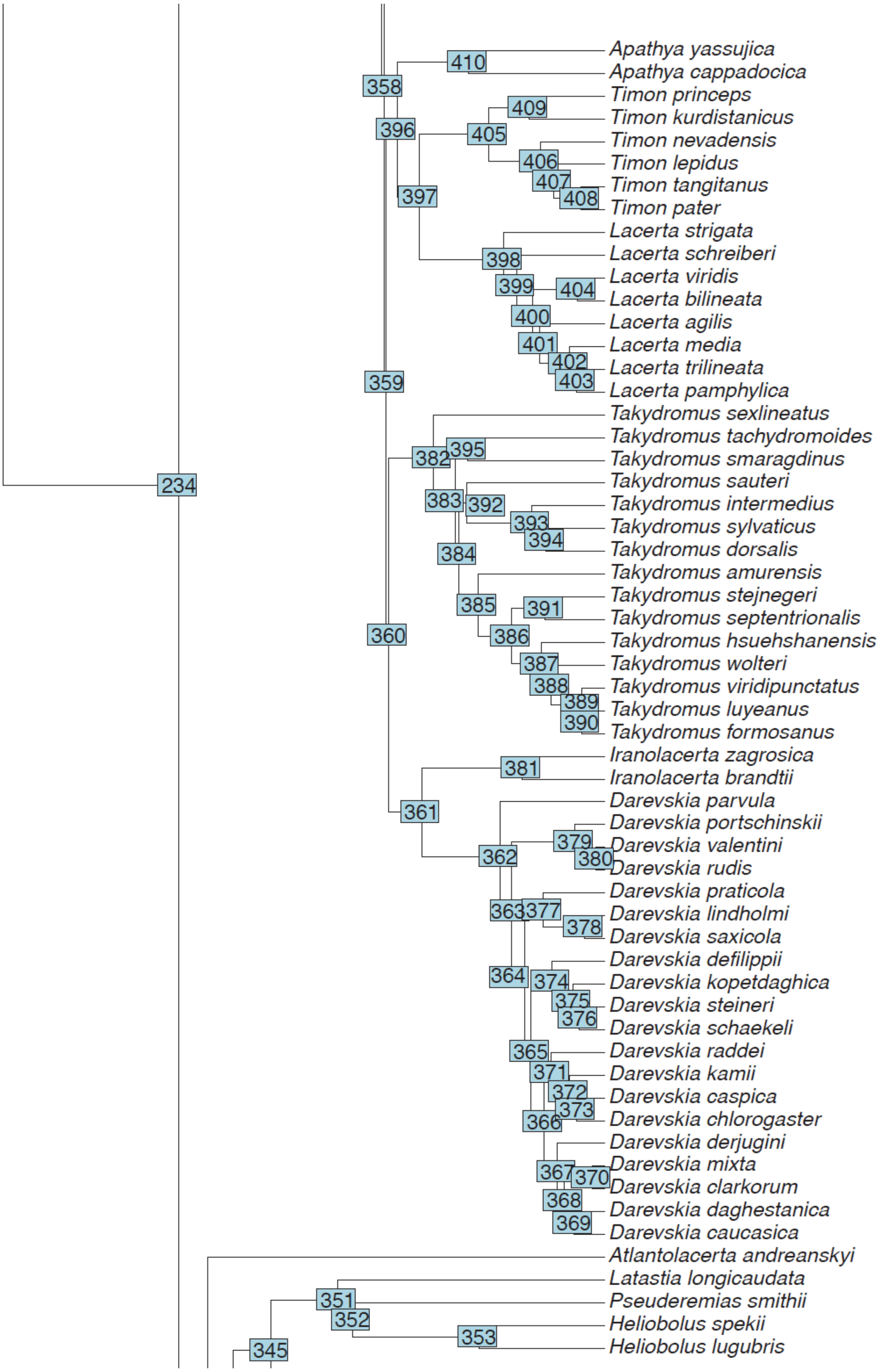

Apathya yassujica
Apathya cappadocica
Timon princeps
Timon kurdistanicus
Timon nevadensis
Timon lepidus
Timon tangitanus
Timon pater
Lacerta strigata
Lacerta schreiberi
Lacerta viridis
Lacerta bilineata
Lacerta agilis
Lacerta media
Lacerta trilineata
Lacerta pamphylica
Takydromus sexlineatus
Takydromus tachydromoides
Takydromus smaragdinus
Takydromus sauteri
Takydromus intermedius
Takydromus sylvaticus
Takydromus dorsalis
Takydromus amurensis
Takydromus stejnegeri
Takydromus septentrionalis
Takydromus hsuehshanensis
Takydromus wolteri
Takydromus viridipunctatus
Takydromus luyeanus
Takydromus formosanus
Iranolacerta zagrosica
Iranolacerta brandtii
Darevskia parvula
Darevskia portschinskii
Darevskia valentini
Darevskia rudis
Darevskia praticola
Darevskia lindholmi
Darevskia saxicola
Darevskia defilippii
Darevskia kopetdaghica
Darevskia steineri
Darevskia schaekeli
Darevskia raddei
Darevskia kamii
Darevskia caspica
Darevskia chlorogaster
Darevskia derjugini
Darevskia mixta
Darevskia clarkorum
Darevskia daghestanica
Darevskia caucasica
Atlantolacerta andreanskyi
Latastia longicaudata
Pseuderemias smithii
Heliobolus spekii
Heliobolus lugubris

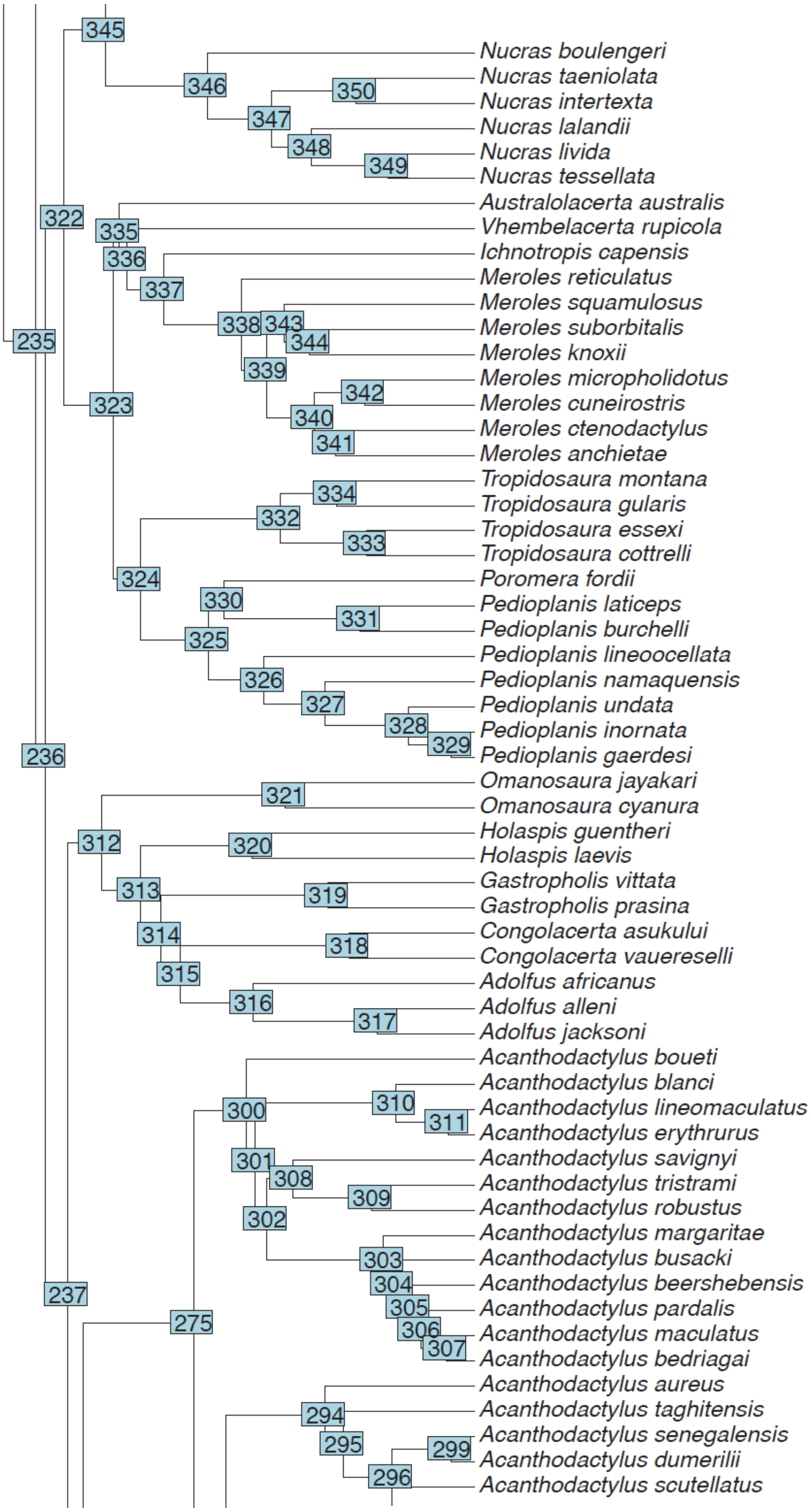
345
346
350
347
348
349
Nucras boulengeri
Nucras taeniolata
Nucras intertexta
Nucras lalandii
Nucras livida
Nucras tessellata
322
335
336
337
338
343
344
339
342
340
341
235
323
Australolacerta australis
Vhembelacerta rupicola
Ichnotropis capensis
Meroles reticulatus
Meroles squamulosus
Meroles suborbitalis
Meroles knoxii
Meroles micropholidotus
Meroles cuneirostris
Meroles ctenodactylus
Meroles anchietae
334
332
333
324
330
331
325
326
327
328
329
236
Tropidosaura montana
Tropidosaura gularis
Tropidosaura essexi
Tropidosaura cottrelli
Poromera fordii
Pedioplanis laticeps
Pedioplanis burchelli
Pedioplanis lineoocellata
Pedioplanis namaquensis
Pedioplanis undata
Pedioplanis inornata
Pedioplanis gaerdesi
321
312
320
313
319
314
318
315
316
317
Omanosaura jayakari
Omanosaura cyanura
Holaspis guentheri
Holaspis laevis
Gastropholis vittata
Gastropholis prasina
Congolacerta asukului
Congolacerta vauereselli
Adolfus africanus
Adolfus alleni
Adolfus jacksoni
300
310
311
301
308
309
302
303
304
305
306
307
237
275
Acanthodactylus boueti
Acanthodactylus blanci
Acanthodactylus lineomaculatus
Acanthodactylus erythrurus
Acanthodactylus savignyi
Acanthodactylus tristrami
Acanthodactylus robustus
Acanthodactylus margaritae
Acanthodactylus busacki
Acanthodactylus beershebensis
Acanthodactylus pardalis
Acanthodactylus maculatus
Acanthodactylus bedriagai
294
295
299
296
Acanthodactylus aureus
Acanthodactylus taghitensis
Acanthodactylus senegalensis
Acanthodactylus dumerilii
Acanthodactylus scutellatus

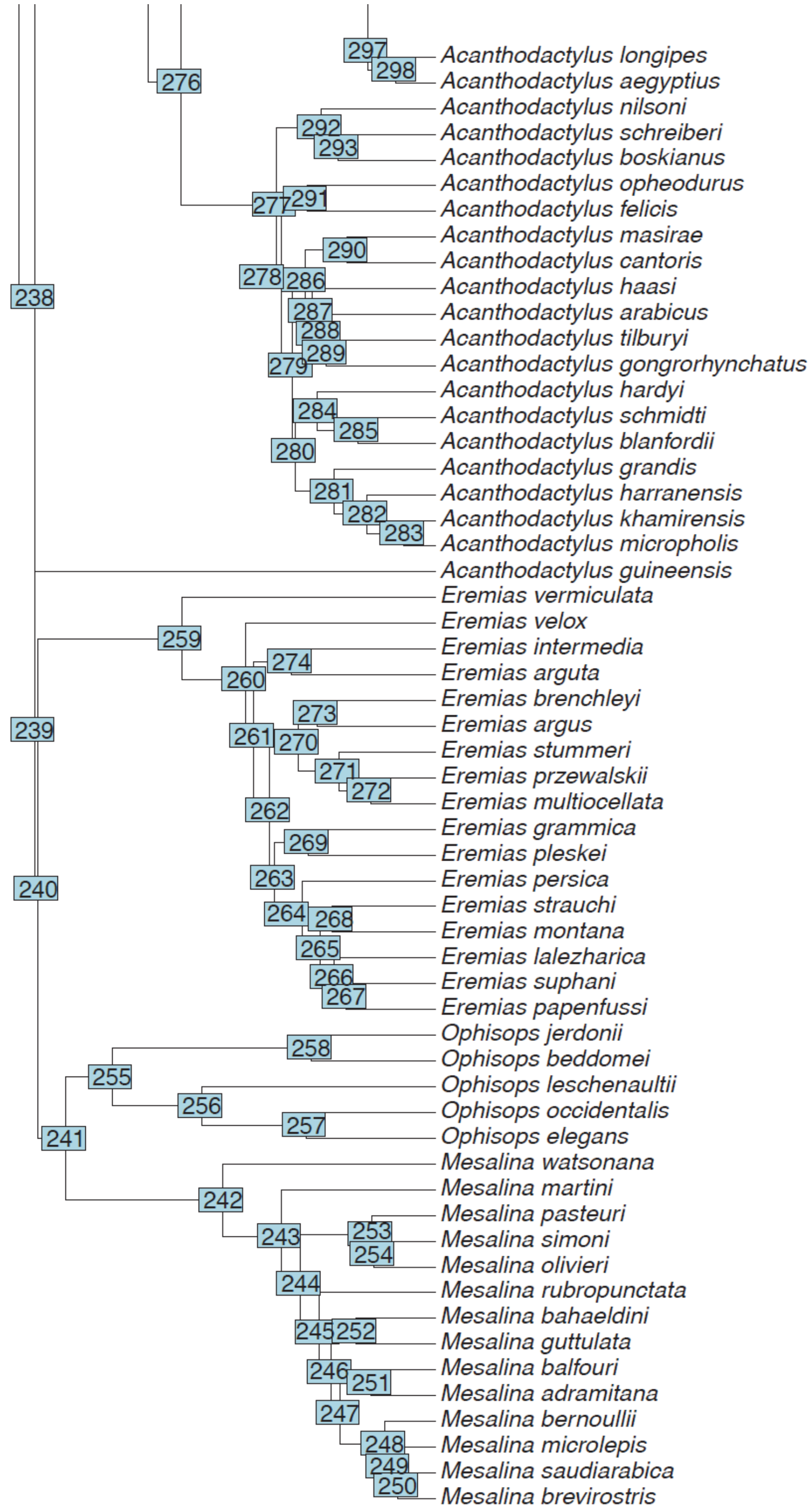

Supplementary Figure 16. Tree with node numbers, corresponding to the tree displaying results of ancestral character state reconstructions for yearly hours >30°C (as visualized in Fig. 2). See Supplementary Table 17 for the reconstructed values and confidence intervals for each node.

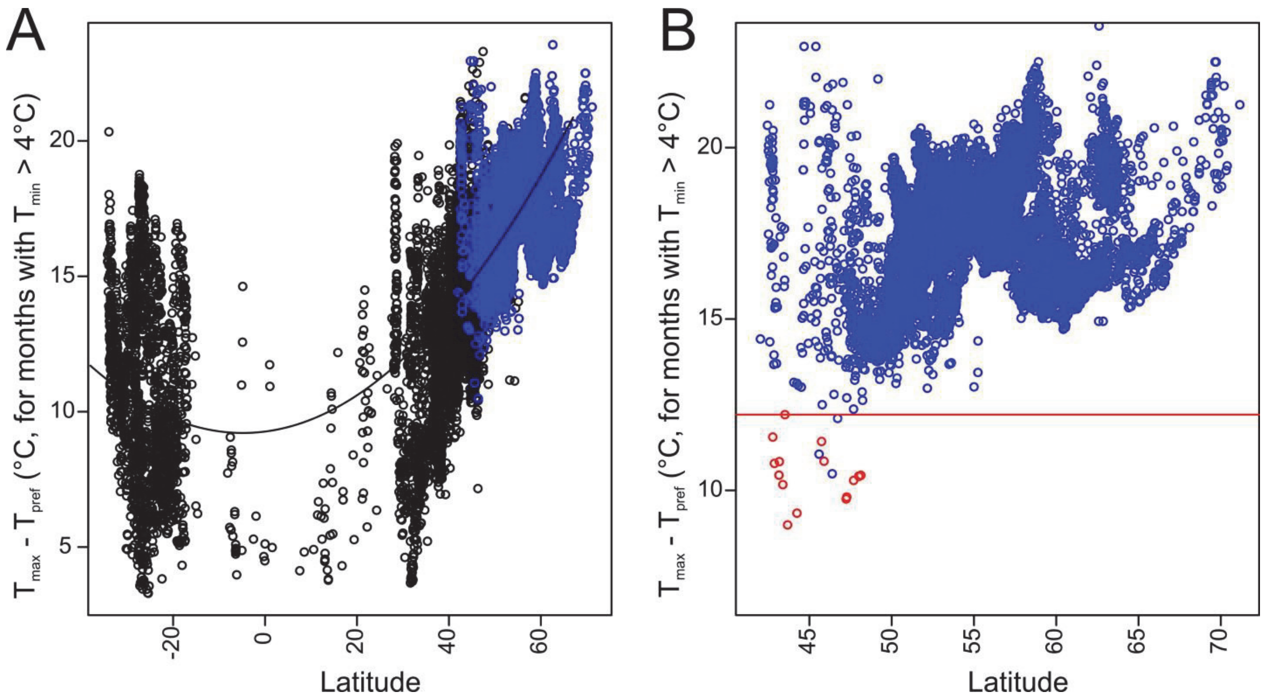

Supplementary Figure 17. Environmental requirements for thermoregulation and temperature-driven extinction risk. Both plots show latitude vs. the difference (in °C) between T_{pref} of a lacertid species, and maximal temperature (T_{max}) averaged across all months (excluding the coldest months) for all occurrence records of the species. (A) Plot for all lacertid species (N=56 species, N=29919 occurrence records, line represents simple quadratic fit). A smaller difference indicates that the preferred temperature can often be reached without extensive basking whereas large differences suggest a need for extensive basking behaviour (i.e., heliotherm thermoregulators). A smaller difference of 0–5°C characterizes populations that may be vulnerable to extinction through global warming. Blue dots correspond to the northernmost occurring species, *Zootoca vivipara*. (B) Data for *Z. vivipara* only. Red dots correspond to additional localities in which extinctions have been recorded [93], the red line marking the T_{max} - T_{pref} values that represent the contemporary limit for observed extirpations. Source data are provided as a Source Data file.

Supplementary Table 1. Specimens and voucher numbers of extant taxa that were microCT scanned and used for scoring. Specimens marked with an asterisk are digital 3D files publicly available on digimorph.org that were originally scanned at the University of Texas at Austin. Institutional abbreviations: FMNH: Field Museum of Natural History Chicago, USA; G: Amber Museum Gdańsk, Danzig, Poland; MGPU: Zoologisches Museum – Georg-August-Universität, Göttingen, Germany; MNCN: Museo Nacional de Ciencias Naturales, Madrid, Spain; ZFMK: Zoologisches Forschungsmuseum Alexander Koenig, Bonn, Germany; ZMB: Museum für Naturkunde Berlin, Germany; ZSM: Zoologische Staatssammlung München, Germany.

Species	Voucher specimens
Acanthodactylus aureus	ZMB13743
Acanthodactylus boskianus	ZMB15168; ZMB70859; ZMB15150
Acanthodactylus lineomaculatus	ZMB 1078
Adolfus africanus	ZFMK 88168; ZFMK 81207; ZFMK41093
Adolfus jacksoni	ZMB48254; ZFMK71724
Algyroides fitzingeri	ZSM0254
Algyroides marchi	MNCN31537; MNCN31538; MNCN31539; MNCN7948; MNCN32095; MNCN32122; MNCN32083; MNCN32085
Algyroides moreoticus	ZMB18777; ZMB18415a; ZMB18415b; ZMB18778; ZMB18778b
Algyroides nigropunctatus	ZMB47112; ZMB47113; ZMB47115; ZMB47114
Anatololacerta danfordi	ZMB38353
Apathya cappadocica	ZMB45846; ZMB45847
Archaeolacerta bedriagae	ZMB69157; ZMB69158
Atlantolacerta andreanskyi	ZMB76686
Australolacerta australis	ZSM0316
Blanus cinereus	ZSM 175-1993-1; ZSM 175-1993-2; ZSM 652-0-1; ZSM 652-0-2
Congolacerta vauereselli	ZMB2834; ZFMK 58683
Dalmatolacerta oxycephala	ZMB42347; ZMB60284
Darevskia daghestanica	ZMB80897; ZMB80900; ZMB80901; ZMB80899; ZMB80902
Darevskia derjugini	ZMB42313; ZMB42314
Darevskia parvula	ZMB44574; ZMB44573
Darevskia praticola	ZMB75171; ZMB75174; ZMB75172; ZMB75173
Darevskia saxicola	ZMB39081a; ZMB39081b; ZMB39081c; ZMB39081d
Dinarolacerta mosorensis	ZMB29252; ZMB29303
Eremias argus	ZMB1940b
Eremias arguta	ZMB37830; ZMB8748
Gallotia caesaris	ZMB38335; ZMB 29489; ZMB32877
Gallotia galloti	ZMB955; ZMB30554
Gallotia stehlini	ZMB29084
Gastropholis prasina	ZMB18358; ZMB18087
Heliobolus spekii	ZMB19796; ZMB48324; ZMB48325
Hellenolacerta graeca	ZMB18412
Holaspis guentheri	ZMB27197; ZMB16108; ZMB 16851
Holaspis laevis	ZMB74409
Iberolacerta aranica	ZMB12666a
Iberolacerta bonnali	MNCN33189; MNCN33190; MNCN33192; MNCN13861; MNCN13860
Iberolacerta cyreni	MNCN33062; MNCN6403; MNCN6382; MNCN33293; MNCN33245; MNCN33291; MNCN33246;
Iberolacerta galani	MNCN33100; MNCN33101; MNCN13862; MNCN33102
Iberolacerta horvathi	ZMB23666a; ZMB23666b

Iberolacerta monticola	MNCN10982; MNCN10983; MNCN10985; MNCN10986; MNCN10988; MNCN33211
Ichnotropis capensis	ZMB25829; ZMB15564
Iranolacerta brandtii	ZMB37965; ZMB71092
Lacerta agilis	ZMB28322; ZMB69529; ZMB69531; ZMB69532; ZMB69449
Lacerta bilineata	ZMB60298; ZMB36201a; ZMB36201b; ZMB36201c
Lacerta media	MNCN7563; ZMB38481
Lacerta schreiberi	ZMB38343; ZMB80924; ZMB11024; ZMB21805; ZMB39414; ZMB43464; ZMB43607;
Lacerta strigata	ZMB58218; ZMB58219; ZMB58220; ZMB58221; ZMB58221; ZMB58222; ZMB39145;
Lacerta trilineata	ZMB38671a; ZMB38671b; ZMB38671c; ZMB38671d
Lacerta viridis	ZMB9254; ZMB9254b; ZMB42830
Latastia longicaudata	ZMB19784; ZMB22443; ZMB74545
Meroles squamulosus	ZMB6615
Mesalina olivieri	ZMB79491; ZMB79492
Nucras boulengeri	ZMB29823
Omanosaura cyanura	ZSM0253
Omanosaura jayakari	ZMB 50029
Ophisops microlepis	ZMB9938
Parvilacerta parva	ZMB38384a; ZMB38384b
Pedioplanis laticeps	ZMB23443
Podarcis bocagei	ZMB10644; ZMB10673; ZMB74011; ZMB74012; ZMB10702; ZMB74013; ZMB74014;
Podarcis carbonelli	MNCN17335; MNCN15828; MNCN15829; MNCN15803; MNCN15801; MNCN10013
Podarcis erhardii	ZMB38528a; ZMB38528b; ZMB38528c; ZMB38528d; ZMB38528e
Podarcis lilfordi	ZMB24575; ZMB31654; ZMB29117; ZMB31975; ZMB31976
Podarcis liolepis	MNCN44286
Podarcis muralis	ZMB10966; ZMB11023; ZMB38496a; ZMB73919; ZMB73921; ZMB73920; ZMB38496b;
Podarcis pityusensis	ZMB36007; ZMB 35987h; ZMB 35987f; ZMB 35987e; ZMB52840; ZMB35982; ZMB 35975; ZMB 32298a
Podarcis siculus	ZMB38504a; ZMB38504b; ZMB38504c; ZMB38504d; ZMB38504e
Podarcis tiliguerta	MNCN10174
Poromera fordii	ZMB15526
Psammodromus algirus	ZMB26522; ZMB17934; ZMB17932b; ZMB17932a; ZMB19611; ZMB17870; ZMB17931;
Psammodromus blanci	ZMB19631; ZMB19630; ZMB14484a; ZMB14484b
Psammodromus hispanicus	ZMB6352; ZMB8553; ZMB10662a; ZMB10662b; ZMB10685; ZMB10968; ZMB15563;
Scelarcis perspicillata	ZMB 14245
Takydromus amurensis	ZMB9869
Takydromus sexlineatus	ZMB69327; ZMB54776
Takydromus tachydromoides	ZMB4309a; ZMB4309b; ZMB26625a; ZMB26625b; ZMB26625c; ZMB26625d; ZMB26625e;
**Teius teyou*	FMNH10873
Timon lepidus	ZMB10680; ZMB17872; ZMB9608a; ZMB9608b; ZMB9608c; ZMB10681
Timon pater	ZMB14869a; ZMB14869b
Timon princeps	MNCN6043; MNCN6066; MNCN6056
**Tupinambis teguixin*	FMHN22416
Vhembelacerta rupicola	ZMB660
Zootoca vivipara	ZMB75196; ZMB75197; ZMB75198; ZMB75199; ZMB75200

Supplementary Table 2. Characters and character states scored in fossil and recent Lacertidae, used to optimize the position of fossils in the phylogenetic tree, in order to use them as time constraints for divergence dating. The 89 characters were scored in 92 lacertid species, with a focus on cranial morphology, based on Arnold [88] who used 21 characters, and Arnold et al. [89] who used 12 characters. Seven of the essential characters of these authors were not used, mostly because of high interspecific variation, and others were redefined more precisely based on linear measurements on 3D microCT-Scans to find thresholds to distinguish between to character states. The characters refer to the cranium (34 characters), mandible (12), postcranium (7), scales of the head (12), scales of the body (11), and soft tissue and behaviour (11). With a few exceptions we scored 3-5 specimens per species, for 86 extant specimens of 36 genera. Characters marked with an asterisk are new.

**Character number**	**Description of character and character states**
	**Snout:**
1	Nares are small (0) or large (1) (modified from [88])
2	Frontal-nasal suture almost straight (1) or round (0) *
3	Septomaxilla is simply convex above with a rounded margin and a narrow anterior shelf with at most slight anterior and posterior projections (0), with distinct and widely separated anterior and posterior projections (1), with distinct and widely separated anterior and posterior projections and a clear posterolateral process (2), with distinct posterior projection and posterolateral process but without anterior projection (3), with distinct posterior projection and posterolateral process and without anterior projection but with an anterolateral process (4), like 4 but anterolateral process posteriorly orientated (5), anteriolateral processes with anterior processes (modified from [88])
4	Medial depression on snout is absent (0) or present (1) [88,89]
5	Nasal process of premaxilla: (0) broad; (1) intermediate; (2) slender (modified from [89])
6	Nasal process of premaxilla: broadest point anterior (0); broadest point not anterior (1); straight (2) *
7	Antero-lateral process of maxilla not in contact with premaxilla (0), slightly in contact (1) or in contact with overlap (2)*
8	Antero-medial process of maxilla single-ended (0), double-ended (1) or double-ended like a fork (2)*
9	Anterior (lateral) descending processes of frontal bone present and well developed (1), reduced or absent (0) [88,89]
10	Premaxillary tooth count less than 8 (0) or equal to or more than 8 (1) *
11	Maxillary tooth count less than 16 (0), 16 -18 (1) or more or than 18 (2) *
12	Maxillary tooth crown size and height: (0) increases posteriorly or (1) constant throughout tooth row (modified from [90])
13	Pterygoid teeth absent (0) or present (1) (modified from [89])
14	Teeth on maxilla are slender with one or two cusps (0). broader posteriorly and often tricuspid (1), or clearly tri- or polycuspid (2) *
	**Skull roof & occipital region:**
15	Fronto-parietal suture more or less straight (0) or interdigitating (1) [88]
16	General outline of fronto-parietal suture is linear (0), forming an anteriorly convex curve (1) forming multiple curves (2) *
17	Parietal table is longer than broad (0) broader than long (1) or as broad as long (2) in adults (modified from [88])
18	Braincase is well-visible in dorsal view (1) or almost completely roofed by the parietal table (0) (modified from [88])
19	Median crest on ventral side of parietal is absent (0) or present (1) in adults
20	Osteodermal layer on the skull roof is weakly developed or absent (0), or strongly developed (1) [88]
21	Temporal scales are not ossified (0) or ossified, at least partially (1) [88]
22	Squamosal as long as postorbital (0) or or much shorter (1) *
23	Quadrojugal process clearly present (0) or barely visible or absent (1) [88]
24	Broadest point of skull at quadrate (0), at jugal (1) or along both (2) *
25	Frontals are discrete (0), partially fused in later ontogeny (1) or fused (2) [88,91]

26	Postorbital and postfrontal fused (0) or clearly separated (1) [88,89]
27	Ossified bar between parietal and supraoccipital absent (0) or present (1) *
28	Foramen magnum relative posterior extent: supraoccipital (0) or presents the posteriormost point (1)
29	Distance between parietal and supraoccipital smaller than diameter of foramen magnum (0) or not (1) *
30	Foramen magnum round (0), mediolaterally broader than dorsoventrally high (1) or higher than broad (2) *
31	Subolfactory process of frontal well developed and in contact with palatine (0), absent (1) or prefrontal intersects between frontal and palatine (2) *
32	Distal end of subolfactory process of frontal broad and sometimes interdigitating (0) or long and narrow (1) *
33	Basipterygoid process of parabasisphenoid strongly bent ventrally (0) or almost at level with remaining part of parabasisphenoid
34	Supratemporal processes of parietal long and slender (0) or short or formed like a short triangle (1)
	**Mandible:**
35	Teeth on dentary slender with one or two cusps (0) or broader posteriorly and often tricuspid (1) *
36	Number of dentary teeth <20 (0), 21 - 25 (1), >25 (2) *
37	Ossified mandibular scales absent (0) or present (1) [88]
38	Coronoid muscle attachment form type A (0), type B (1) *
39	Medial posterior coronoid process type A (0), type B (1) or type C (1) *
40	Medial posterior coronoid process round (0) or with at least one edge (1) *
41	Coronoid process more than 1/3 (0) or less than 1/3 of mandibular height (1)
42	Medial crest of coronoid absent (0) or present (1) (modified from [88])
43	Antero-lateral process of coronoid ends anterior to posterior most tooth (0), at level with posterior most tooth (1) or posterior to posterior most tooth (2) *
44	Coronoid without lateral process (0), with a posterior process (1) or with a well visible ventral oriented process (2)
45	General form of mandibles in ventral view: shape like a downturned “V” (1), with posterior parts parallel to each other (0) or with posterior margins posterolaterally extending (2)
46	Thickness of mandibles relative to the teeth: relatively massive (0) or more delicate (1)
47	Lateral crest on mandible present (0) or absent (1) [88]
	**Postcranium:**
48	Number of presacral vertebrae <25 (0), 26 (1), 27 (2) [89]
49	Medial loop of clavicle without loop (0), with interrupted loop (1) or with complete loop (2) [88,89]
50	Lateral arms of interclavicle directed laterally or obliquely forwards (0); directed obliquely backwards (1) [89]
51	Sternal fontanelle oval or round (0) or markedly heart-shaped (1) (modified from [88,89])
52	Inscriptional ribs are present (0) or absent (1) [88,89]
53	Number of posterior presacral vertebrae with short ribs: often five or fewer (0); often six (1); often seven or more (2) [89]
54	Tail vertebrae: with A-pattern only (0), A-and B-patterns (1), BC-pattern (2), C-pattern (3) [89]
55	Course of ulnar nerve: 'lacertid'-like (0), intermediate (1) or 'varanid'-like (2) [88]
	**Head scalation:**
56	Masseteric scale: absent (0) or present (1) [88,89]
57	Contact between rostral and frontonasal scales: absent (0) or present (1) [89]
58	Outer edge of parietal scale not reaching lateral border of parietal table (0), reaching lateral border of parietal table only posteriorly (1), or reaching lateral border of parietal table both posteriorly and anteriorly (2) [89]
59	Occipital scale more narrow (0) or as broad as posterior border of frontal scale (1) (sensu [89])
60	Number of postnasal scales: one (0) or two (1) [88,89]
61	Contact between supranasal scale and anterior loreal scale above single postnasal scale: mostly absent (0) or mostly present (1) [88,89]
62	Nostril separated from the first upper labial scale by contact between the postnasal and supranasal scales below: mostly absent (0) or mostly present (1) [88,89]
63	Number of upper labial scales in front of subocular scale: four (0) or five or more (1) (modified from [88,89])
64	Lower eyelid without transparent window (0) or with a transparent window of one or more scales (1) [88,89]
65	Interparietal scale in contact with occipital scale: yes (0) or no (1)*
66	Relative position of the frontoparietal scale on frontal bone: covering the posterior third of the

	frontal bone (0) or covering the posterior half (1) *
	**Body scalation:**
67	Dorsal scales on body smaller than those on upper surface of tail (0) or larger (1) [89]
68	Collar beneath throat: absent (0) or present (1) [88,89]
69	Posterior edge of collar: smooth (0) or clearly serrated (1) (modified from [89])
70	Number of longitudinal rows of ventral scales: usually six (0), usually eight (1), ten or more (2) [89]
71	Shape of ventral scales: rectangular without little posterior overlap (0), with slightly sloping sides and overlapping posteriorly (1) or with strongly sloping sides and strongly overlapping posteriorly (2) [89]
72	Anterior semicircles of enlarged scales around preanal scale zero to one (0), sometimes 2 (1) or 2-3 (2) [89]
73	Dorsal scales are small and round (0), or large and diamond shaped with a keel [89]
74	Keeling on ventral body scales: absent (0), outer row on each side keeled (1) or all rows are keeled (2) [88,89]
75	Lateral scale rows on fingers: present (0) or absent (1) [88,89]
76	Lateral scale rows on toes: present (0) or absent (1) [88,89]
77	First scale posteriorly to the toe claw: not exposed ventrally (0), or strongly exposed and convex (1) [89]
78	Scales bordering ventral mid-line of tail: narrow and not much wider than adjoining scales (0) or broad and much wider than adjoining scales (1) [88,89]
	**Miscellaneous soft-part characters**
79	Lateral septum on bodenaponeurosis: present (0) or absent (1) [89]
80	Insertion of retractor lateralis anterior muscle in front of vent: near mid-line (0) or more laterally (1) [89]
81	Size of retractor lateralis anterior muscle in front of vent: narrow, with no fibres extending to region of vent lip (0) or broad, with some fibres extending posteriorly to region of vent lip (1) [89]
82	Some fibres of retractor lateralis anterior muscle reaching base of hemipenis: no (0), yes (1) [89]
83	Hemipenial armature: absent (0), traces (1) or strongly developed (2) [89]
84	Cross section of lobes in retracted hemipenis: simple (0), with limited folding (1) or with complex folding (2) [89]
85	Distal section of hemipenial lobes: not longer than proximal section (0) or longer (1) [89]
86	Hemipenial microornamentation on distal lobes: hook-shaped spines (0), crown-shaped tubercles (1) or bicuspid tubercles (2) [89]
87	Hemipenial microornamentation on sides of lobes: made up of long spines (1) or not (0) [89]
	**Behavior:**
88	Voice: mute (0) or speaks frequently (1) [88]
89	Copulatory position: flank-bite (0) or neck-bite (1) [88]

Supplementary Table 3. Results of mixed Spatial Auto-Regressive (SAR) models on the influence of bioclimatic variables for species richness of Lacertidae, and separately for our main target clade (Lacertini). See Supplementary Methods for an explanation of variables.

Variable	OLS Coeff.	SAR Coeff.	Std Coeff.	Std Error	t	P Value
**Analysis: All Lacertidae**						
Constant	6.41	-1.322	0	0.845	-1.564	0.118
hours >4°C and >100W/m^2	0.003	0.002	0.692	<.001	9.97	<.001
bioSR3	0.001	<.001	0.09	<.001	4.231	<.001
bioSR4	<.001	0.002	0.102	<.001	4.895	<.001
bio27	<.001	<.001	-0.395	<.001	-6.193	<.001
bio26	<.001	<.001	0.317	<.001	10.736	0
bio23	-0.081	-0.053	-0.584	0.006	-8.48	<.001
bio1	0.008	0.005	0.286	0.002	3.451	<.001
bio10	-0.003	-0.006	-0.191	0.002	-3.38	<.001
bio15	0.014	0.009	0.125	0.001	6.016	<.001
bio16	-0.004	-0.003	-0.306	<.001	-11.461	<.001
bio3	-0.046	-0.042	-0.313	0.005	-7.845	<.001
bio8	-0.01	-0.006	-0.181	<.001	-7.599	<.001
**Descriptive Statistics: All Lacertidae**	**Species Richness**	**Estimated**	**Error**			
Min	0	-4.392	-5.651			
Max	19	7.345	15.081			
Mean	2.753	2.785	-0.032			
Std.Dev.	2.514	1.675	1.874			
Skewness	1.409	0.153	1.803			
Kurtosis	-563.578	-17.431	-1396.46			
**Statistics details: All Lacertidae**						
	n: 3377	F: 136.67	P: <.001			
OLS Result:	r: 0.627	r^2: 0.393	AICc: 14158.259			
Explained by Predictor Variables:	r: 0.588	r^2: 0.346	AICc: 14409.469			
Total Explained (Predictor + Space):	r: 0.666	r^2: 0.444	AICc: 13859.06			
Spatial autoregressive parameter (rho): 0.936						
Alpha: 1.0						
**Variable**	**OLS Coeff.**	**SAR Coeff.**	**Std Coeff.**	**Std Error**	**t**	**P Value**
**Analysis: Lacertini**						
Constant	4.401	0.433	0.000	0.510	0.850	0.396
bio9	-0.002	-0.002	-0.319	<.001	-6.317	<.001
bio6	0.006	0.005	0.619	<.001	9.614	<.001
bio5	-0.005	-0.007	-0.379	<.001	-12.345	0.000
bio3	-0.057	-0.052	-0.664	0.003	-17.057	0.000
bio23	-0.031	-0.022	-0.404	0.003	-6.820	<.001
bioSR1	<.001	<.001	0.094	<.001	5.869	<.001
bioSR2	<.001	0.002	0.158	<.001	8.464	<.001
bio27	<.001	<.001	-0.728	<.001	-11.831	<.001
bio26	<.001	<.001	0.162	<.001	6.142	<.001
hours >4°C and >100W/m^2	0.001	0.001	0.710	<.001	14.392	0.000
**Descriptive Statistics: Lacertini**	**Species Richness**	**Estimated**	**Error**			
Min	0	-2.521	-3.277			
Max	15	5.244	13.582			
Mean	0.865	0.859	0.006			
Std.Dev.	1.473	0.89	1.174			
Skewness	3.249	0.418	3.541			
Kurtosis	-3030.623	-26.432	-4816.77			
**Statistics details: Lacertini**						
	n: 3377	F: 177.497	P: <.001			
OLS Result:	r: 0.623	r^2: 0.388	AICc: 10569.661			
Explained by Predictor Variables:	r: 0.588	r^2: 0.345	AICc: 10796.813			
Total Explained (Predictor + Space):	r: 0.604	r^2: 0.365	AICc: 10694.152			
Spatial autoregressive parameter (rho): 0.936						
Alpha: 1.0						
**Analysis: Eremiadini**						
Constant	2.086	-1.275	0	0.649	-1.965	0.049
hours >4°C and >100W/m^2	<.001	<.001	-0.122	<.001	-2.202	0.028
bioSR3	<.001	<.001	0.046	<.001	2.444	0.015
bio27	<.001	<.001	-0.307	<.001	-4.234	<.001
bio25	<.001	<.001	-0.102	<.001	-2.834	0.005
bio23	-0.034	-0.024	-0.271	0.005	-4.774	<.001
bio1	-0.011	-0.012	-0.639	0.002	-6.128	<.001
bio10	0.016	0.015	0.481	0.002	9.035	<.001
bio15	0.013	0.009	0.14	0.001	7.052	<.001
bio16	-0.003	-0.003	-0.302	<.001	-13.843	<.001
bio17	-0.003	-0.002	-0.063	<.001	-3.497	<.001
bio2	-0.004	-0.006	-0.071	0.002	-2.992	0.003

bio3	0.028	0.027	0.208	0.005	5.311	<.001
bio8	-0.009	-0.006	-0.196	<.001	-6.688	<.001
bio9	0.006	0.005	0.39	<.001	5.744	<.001
ANNUAL MEAN Solar Radiation	<.001	<.001	0.624	<.001	9.275	<.001

**Descriptive Statistics: Eremiadini**	**Species Richness**	**Estimated**	**Error**
Min	0	-4.618	-4.207
Max	13	6.01	9.488
Mean	1.861	1.86	0.001
Std.Dev.	2.39	1.741	1.637
Skewness	1.263	0.383	1.247
Kurtosis	-193.874	144.475	-743.022
**Statistics details: Eremiadini**			
	n: 3377	F: 233.378	P: <.001
OLS Result:	r: 0.724	r^2: 0.525	AICc: 12992.004
Explained by Predictor Variables:	r: 0.714	r^2: 0.51	AICc: 13092.918
Total Explained (Predictor + Space):	r: 0.729	r^2: 0.531	AICc: 12947.11
Spatial autoregressive parameter (rho): 0.797			
Alpha: 1.0			

Supplementary Table 4. Phylogenetic signal of morphological, bioclimatic and physiological variables across the lacertid tree (Blomberg's K). Analyses were performed for all Lacertidae (Gallotiinae+Lacertini+Eremiadini), and for Lacertini and Eremiadini separately. Bioclimatic variables are the medians of all occurrence points of a species. See Supplementary Methods for an explanation of variables. Values shown only for calculations with sample sizes of N>10; analyses estimated with low species samples (N<50) should be interpreted with caution.

Variable	K	K's p_value	N (number of spp)
all Lacertidae			
MORPH.1MDS.1	0.891371046	0.001	80
IWL.Griggs.Residual...1000	0.275778334	0.126	51
Tpref.Median.corrected	0.45624383	0.009	58
Number.genes.under.selection	0.459403005	0.907	21
Root.to.tip.length	4.633928783	0.001	243
bio01	0.682954609	0.001	239
bio02	0.539323341	0.001	239
bio03	1.524278004	0.001	239
bio04	0.76642058	0.001	239
bio05	0.662302338	0.001	239
bio06	0.581670086	0.001	239
bio07	0.556717549	0.001	239
bio08	0.549369526	0.001	239
bio09	0.545386774	0.001	239
bio10	0.623729135	0.001	239
bio11	0.682906966	0.001	239
bio12	0.714443083	0.001	239
bio13	0.611960042	0.001	239
bio14	0.295821152	0.002	239
bio15	0.388501066	0.001	239
bio16	0.638571358	0.001	239
bio17	0.32838348	0.001	239
bio18	0.982450018	0.001	239
bio19	0.373392002	0.001	239
Hours >30°C	0.681676497	0.001	232
SVL	0.348912121	0.001	239
Tb	0.565572824	0.001	239
Range size	0.248794802	0.001	239
hours >4°C and >100W/m^2	0.551247496	0.001	239
Eremiadini			
MORPH.1MDS.1	0.83719283	0.382	27
Tpref.Median.corrected	0.864891327	0.033	22
Root.to.tip.length	3.462308236	0.001	124
bio01	0.975720349	0.001	122
bio02	0.476707713	0.001	122
bio03	1.570953595	0.001	122
bio04	1.189955542	0.001	122
bio05	0.869792287	0.001	122
bio06	0.973479547	0.001	122
bio07	0.902091922	0.001	122
bio08	0.508217184	0.001	122
bio09	1.06939918	0.001	122
bio10	0.984554928	0.001	122
bio11	1.078030965	0.001	122
bio12	1.234557946	0.001	122
bio13	0.771976104	0.006	122
bio14	1.356357423	0.001	122

bio15	0.415338257	0.011	122
bio16	0.825159197	0.002	122
bio17	1.468890691	0.001	122
bio18	1.32402122	0.001	122
bio19	0.741250801	0.001	122
Hours >30°C	0.659725997	0.001	120
SVL	0.559141	0.001	122
Tb	0.586228	0.025	122
Range size	0.29001	0.783	122
hours >4°C and >100W/m^2	1.402283	0.001	122
<u>Lacertini</u>			
MORPH.1MDS.1	1.764536797	0.001	47
IWL.Griggs.Residual...1000	0.314800276	0.492	43
Tpref.Median.corrected	0.42920865	0.213	33
Number.genes.under.selection	0.843405545	0.76	17
Root.to.tip.length	2.677919868	0.001	105
bio01	0.645475458	0.001	105
bio02	0.584576456	0.001	105
bio03	0.564840713	0.001	105
bio04	0.515971072	0.001	105
bio05	0.689976566	0.001	105
bio06	0.534076317	0.001	105
bio07	0.521630199	0.001	105
bio08	1.005823701	0.001	105
bio09	0.661022541	0.001	105
bio10	0.68642969	0.001	105
bio11	0.533719939	0.001	105
bio12	0.970698274	0.001	105
bio13	1.244646667	0.001	105
bio14	0.434230167	0.006	105
bio15	0.52737519	0.001	105
bio16	1.144086873	0.001	105
bio17	0.455429429	0.001	105
bio18	1.871363259	0.001	105
bio19	0.353406281	0.009	105
Hours >30°C	0.729893623	0.001	101
SVL	1.202868	0.001	105
Tb	0.541497	0.025	105
Range size	0.364158	0.005	105
hours >4°C and >100W/m^2	0.49238	0.001	105

Supplementary Table 5. Results of Response screening (calculated in JMP 13.0) for all bioclimatic predictor variables considered in this study, for the three response variables representing $T_{pref,}$ IWL, and root-to-tip paths, across all Lacertidae. Variables sorted by decreasing effect size and coloured by FDR-corrected LogWorth values. LogWorth represents -log10 transformed P-values; higher values correspond to lower P-values. FDR refers to P-values adjusted to control the false discovery rate for multiple tests, calculated using the Benjamini-Hochberg technique.

Y	X	Count	PValue	LogWorth	FDR PValue	FDR LogWorth	Effect Size	Rank Fraction	YMean	SSE	DFE	MSE	F Ratio	RSquare
Tpref Median corrected	MED_bio15	58	1.17E-06	5.93212	0.00004	4.40065	0.48562	0.02941	35.69621	86.09797	56	1.53746	29.70542	0.34660
Tpref Median corrected	MED_HoursAbove30C	58	4.81E-05	4.31830	0.00055	3.26289	0.41852	0.05882	35.69621	97.84730	56	1.74727	19.41406	0.25743
Tpref Median corrected	MED_bio20	58	6.67E-05	4.17615	0.00057	3.24673	0.41168	0.11765	35.69621	98.94606	56	1.76689	18.57661	0.24909
Tpref Median corrected	MED_bio27	58	0.000101	3.99590	0.00058	3.24029	0.40275	0.14706	35.69621	100.35408	56	1.79204	17.53027	0.23841
Tpref Median corrected	MED_bio23	58	0.000101	3.99362	0.00058	3.24029	0.40264	0.17647	35.69621	100.37199	56	1.79236	17.51714	0.23827
Tpref Median corrected	MED_bio01	58	0.000135	3.87053	0.00065	3.18415	0.39637	0.20588	35.69621	101.34296	56	1.80970	16.81278	0.23090
Tpref Median corrected	MED_bio08	58	0.000345	3.46252	0.00139	2.85556	0.37445	0.23529	35.69621	104.61478	56	1.86812	14.53556	0.20607
Tpref Median corrected	MED_bio05	58	0.000369	3.43279	0.00139	2.85556	0.37278	0.26471	35.69621	104.85626	56	1.87243	14.37312	0.20424
Tpref Median corrected	MED_bio11	58	0.000545	3.26359	0.00173	2.76290	0.36308	0.29412	35.69621	106.23871	56	1.89712	13.45737	0.19375
Tpref Median corrected	MED_bioSR3	58	0.000558	3.25299	0.00173	2.76290	0.36246	0.32353	35.69621	106.32577	56	1.89867	13.40050	0.19309
Tpref Median corrected	MED_bio24	58	0.001231	2.90977	0.00313	2.50500	0.34159	0.35294	35.69621	109.17103	56	1.94948	11.59176	0.17150
Tpref Median corrected	MED_bio17	58	0.001418	2.84824	0.00313	2.50500	0.33767	0.38235	35.69621	109.68639	56	1.95869	11.27418	0.16759
Tpref Median corrected	MED_bio03	58	0.001443	2.84068	0.00313	2.50500	0.33719	0.41176	35.69621	109.74983	56	1.95982	11.23529	0.16710
Tpref Median corrected	MED_bioSR4	58	0.001493	2.82602	0.00313	2.50500	0.33625	0.44118	35.69621	109.87285	56	1.96202	11.16001	0.16617
Tpref Median corrected	MED_bio10	58	0.001538	2.81312	0.00313	2.50500	0.33541	0.47059	35.69621	109.98126	56	1.96395	11.09381	0.16535
Tpref Median corrected	MED_bio06	58	0.001563	2.80603	0.00313	2.50500	0.33495	0.50000	35.69621	110.04084	56	1.96502	11.05748	0.16490
Tpref Median corrected	MED_bio02	58	0.005294	2.27622	0.00900	2.04577	0.29823	0.58824	35.69621	114.54398	56	2.04543	8.42121	0.13072
Tpref Median corrected	MED_bio14	58	0.007334	2.13464	0.01187	1.92538	0.28750	0.61765	35.69621	115.76156	56	2.06717	7.74363	0.12148
Tpref Median corrected	MED_bio04	58	0.016814	1.77433	0.02599	1.58528	0.25802	0.64706	35.69621	118.87550	56	2.12278	6.07387	0.09785
Tpref Median corrected	MED_bio12	58	0.037046	1.43126	0.05476	1.26151	0.22642	0.67647	35.69621	121.84016	56	2.17572	4.56346	0.07535
Tpref Median corrected	MED_bioSR1	58	0.063598	1.19656	0.09010	1.04529	0.20224	0.70588	35.69621	123.84811	56	2.21157	3.58155	0.06011
Tpref Median corrected	MED_Hoursabove_4Cand100W	58	0.075478	1.12218	0.10265	0.98864	0.19404	0.73529	35.69621	124.47734	56	2.22281	3.28036	0.05534
Tpref Median corrected	MED_bio19	58	0.105758	0.97569	0.13830	0.85918	0.17700	0.76471	35.69621	125.70146	56	2.24467	2.70307	0.04605
Tpref Median corrected	MED_bioSR2	58	0.141656	0.84877	0.17838	0.74865	0.16115	0.79412	35.69621	126.73979	56	2.26321	2.22214	0.03817
Tpref Median corrected	MED_bio26	58	0.172606	0.76294	0.20959	0.67862	0.14975	0.82353	35.69621	127.42601	56	2.27546	1.90860	0.03296
Tpref Median corrected	MED_bio09	58	0.332373	0.47837	0.38968	0.40929	0.10687	0.85294	35.69621	129.55697	56	2.31352	0.95612	0.01679
Tpref Median corrected	MED_bio18	58	0.414658	0.38231	0.46995	0.32795	0.09005	0.88235	35.69621	130.19866	56	2.32498	0.67541	0.01192
Tpref Median corrected	MED_bio07	58	0.469168	0.32867	0.50675	0.29521	0.07995	0.91176	35.69621	130.53094	56	2.33091	0.53113	0.00940
Tpref Median corrected	MED_bio16	58	0.476939	0.32154	0.50675	0.29521	0.07857	0.94118	35.69621	130.57347	56	2.33167	0.51272	0.00907
Tpref Median corrected	MED_bio13	58	0.552982	0.25729	0.56974	0.24432	0.06559	0.97059	35.69621	130.93592	56	2.33814	0.35629	0.00632
Tpref Median corrected	MED_bio25	58	0.729168	0.13717	0.72917	0.13717	0.03831	1.00000	35.69621	131.48467	56	2.34794	0.12108	0.00216

Y	X	Count	PValue	LogWorth	FDR PValue	FDR LogWorth	Effect Size	Rank Fraction	YMean	SSE	DFE	MSE	F Ratio	RSquare
IWL Griggs Residual * 1000	MED_bio01	51	0.000778	3.10923	0.00724	2.14041	0.49206	0.05882	0.05709	38.26465	49	0.78091	12.84463	0.20769
IWL Griggs Residual * 1000	MED_bio09	51	0.000875	3.05818	0.00724	2.14041	0.48781	0.08824	0.05709	38.43705	49	0.78443	12.56725	0.20412
IWL Griggs Residual * 1000	MED_bio11	51	0.000994	3.00246	0.00724	2.14041	0.48312	0.11765	0.05709	38.62583	49	0.78828	12.26633	0.20021
IWL Griggs Residual * 1000	MED_bioSR3	51	0.001064	2.97292	0.00724	2.14041	0.48060	0.14706	0.05709	38.72619	49	0.79033	12.10756	0.19814
IWL Griggs Residual * 1000	MED_bio06	51	0.002229	2.65180	0.01256	1.90092	0.45207	0.17647	0.05709	39.82855	49	0.81283	10.41626	0.17531
IWL Griggs Residual * 1000	MED_Hoursabove_4Cand100W	51	0.002618	2.58201	0.01256	1.90092	0.44556	0.20588	0.05709	40.07073	49	0.81777	10.05715	0.17030
IWL Griggs Residual * 1000	MED_bio25	51	0.002956	2.52931	0.01256	1.90092	0.44056	0.23529	0.05709	40.25423	49	0.82151	9.78794	0.16650
IWL Griggs Residual * 1000	MED_bio05	51	0.00431	2.36547	0.01383	1.85903	0.42456	0.26471	0.05709	40.82772	49	0.83322	8.96216	0.15462
IWL Griggs Residual * 1000	MED_bio10	51	0.004426	2.35397	0.01383	1.85903	0.42341	0.29412	0.05709	40.86816	49	0.83404	8.90481	0.15378
IWL Griggs Residual * 1000	MED_bioSR4	51	0.004476	2.34911	0.01383	1.85903	0.42292	0.32353	0.05709	40.88523	49	0.83439	8.88064	0.15343
IWL Griggs Residual * 1000	MED_bio27	51	0.005567	2.25437	0.01568	1.80476	0.41329	0.35294	0.05709	41.21906	49	0.84121	8.41186	0.14652

IWL Griggs Residual * 1000	MED_bio20	51	0.005994	2.22229	0.01568	1.80476	0.40996	0.38235	0.05709	41.33238	49	0.84352	8.25447	0.14417
IWL Griggs Residual * 1000	MED_bio14	51	0.008034	2.09508	0.01951	1.70973	0.39647	0.41176	0.05709	41.78313	49	0.85272	7.63681	0.13484
IWL Griggs Residual * 1000	MED_bio23	51	0.009043	2.04367	0.02050	1.68829	0.39087	0.44118	0.05709	41.96583	49	0.85645	7.39024	0.13106
IWL Griggs Residual * 1000	MED_bio15	51	0.00993	2.00303	0.02110	1.67567	0.38638	0.47059	0.05709	42.11048	49	0.85940	7.19654	0.12806
IWL Griggs Residual * 1000	MED_bio03	51	0.012665	1.89740	0.02484	1.60478	0.37443	0.50000	0.05709	42.48715	49	0.86708	6.69833	0.12026
IWL Griggs Residual * 1000	MED_bio08	51	0.013153	1.88098	0.02484	1.60478	0.37253	0.52941	0.05709	42.54575	49	0.86828	6.62162	0.11905
IWL Griggs Residual * 1000	MED_bio17	51	0.014757	1.83100	0.02641	1.57827	0.36670	0.55882	0.05709	42.72434	49	0.87193	6.38910	0.11535
IWL Griggs Residual * 1000	MED_HoursAbove30C	51	0.020526	1.68770	0.03172	1.49864	0.34942	0.64706	0.05709	43.23699	49	0.88239	5.73237	0.10473
IWL Griggs Residual * 1000	MED_bio04	51	0.023216	1.63422	0.03432	1.46447	0.34275	0.67647	0.05709	43.42842	49	0.88629	5.49112	0.10077
IWL Griggs Residual * 1000	MED_bioSR2	51	0.031663	1.49945	0.04486	1.34818	0.32533	0.70588	0.05709	43.91060	49	0.89613	4.89276	0.09079
IWL Griggs Residual * 1000	MED_bio07	51	0.086874	1.06111	0.11815	0.92757	0.26147	0.73529	0.05709	45.46294	49	0.92782	3.05258	0.05864
IWL Griggs Residual * 1000	MED_bio18	51	0.153901	0.81276	0.20126	0.69625	0.21876	0.76471	0.05709	46.31263	49	0.94516	2.09758	0.04105
IWL Griggs Residual * 1000	MED_bio12	51	0.187353	0.72734	0.23593	0.62723	0.20258	0.79412	0.05709	46.59499	49	0.95092	1.78794	0.03520
IWL Griggs Residual * 1000	MED_bio19	51	0.257311	0.58954	0.31245	0.50522	0.17446	0.82353	0.05709	47.03427	49	0.95988	1.31360	0.02611
IWL Griggs Residual * 1000	MED_bioSR1	51	0.367313	0.43496	0.43064	0.36588	0.13918	0.85294	0.05709	47.49266	49	0.96924	0.82798	0.01662
IWL Griggs Residual * 1000	MED_bio13	51	0.512292	0.29048	0.57675	0.23902	0.10136	0.88235	0.05709	47.86951	49	0.97693	0.43571	0.00881
IWL Griggs Residual * 1000	MED_bio16	51	0.525856	0.27913	0.57675	0.23902	0.09814	0.91176	0.05709	47.89615	49	0.97747	0.40821	0.00826
IWL Griggs Residual * 1000	MED_bio26	51	0.583697	0.23381	0.62018	0.20748	0.08483	0.94118	0.05709	47.99708	49	0.97953	0.30432	0.00617
IWL Griggs Residual * 1000	MED_bio02	51	0.633211	0.19845	0.65240	0.18549	0.07390	0.97059	0.05709	48.06894	49	0.98100	0.23061	0.00468
IWL Griggs Residual * 1000	MED_bio24	51	0.706404	0.15095	0.70640	0.15095	0.05836	1.00000	0.05709	48.15409	49	0.98274	0.14356	0.00292

Y	X	Count	PValue	LogWorth	FDR PValue	FDR LogWorth	Effect Size	Rank Fraction	YMean	SSE	DFE	MSE	F Ratio	RSquare
Root-to-tip length	MED_bio05	239	4.02E-32	31.39536	0.00000	29.90400	0.52698	0.03226	0.15553	0.20536	237	0.00087	189.87655	0.44480
Root-to-tip length	MED_bio01	239	2.36E-29	28.62715	0.00000	27.43682	0.50859	0.06452	0.15553	0.21664	237	0.00091	167.64074	0.41430
Root-to-tip length	MED_HoursAbove30C	232	8.28E-27	26.08212	0.00000	25.12964	0.49469	0.09677	0.15581	0.21661	230	0.00094	149.43599	0.39384
Root-to-tip length	MED_bioSR4	239	1.08E-26	25.96622	0.00000	25.12964	0.48926	0.12903	0.15553	0.22807	237	0.00096	147.37359	0.38341
Root-to-tip length	MED_bio10	239	1.2E-26	25.92203	0.00000	25.12964	0.48893	0.16129	0.15553	0.22826	237	0.00096	147.04592	0.38289
Root-to-tip length	MED_bio20	239	4E-24	23.39764	0.00000	22.68443	0.46885	0.19355	0.15553	0.23965	237	0.00101	128.78892	0.35209
Root-to-tip length	MED_bio23	239	5.88E-23	22.23066	0.00000	21.58439	0.45893	0.22581	0.15553	0.24511	237	0.00103	120.65076	0.33734
Root-to-tip length	MED_bio27	239	1.49E-21	20.82541	0.00000	20.23714	0.44638	0.25806	0.15553	0.25183	237	0.00106	111.09647	0.31915
Root-to-tip length	MED_bioSR3	239	1.42E-18	17.84884	0.00000	17.31172	0.41737	0.29032	0.15553	0.26668	237	0.00113	91.71580	0.27901
Root-to-tip length	MED_bio11	239	1.58E-18	17.80151	0.00000	17.31015	0.41688	0.32258	0.15553	0.26692	237	0.00113	91.41681	0.27836
Root-to-tip length	MED_bio08	239	1.78E-15	14.74994	0.00000	14.29997	0.38288	0.35484	0.15553	0.28303	237	0.00119	72.72472	0.23480
Root-to-tip length	MED_bio02	239	6.65E-15	14.17744	0.00000	13.76525	0.37593	0.38710	0.15553	0.28616	237	0.00121	69.34404	0.22636
Root-to-tip length	MED_bio15	239	9.57E-15	14.01913	0.00000	13.64171	0.37397	0.41935	0.15553	0.28703	237	0.00121	68.41613	0.22401
Root-to-tip length	MED_bio06	239	6.33E-12	11.19841	0.00000	10.85317	0.33616	0.45161	0.15553	0.30294	237	0.00128	52.37702	0.18100
Root-to-tip length	MED_bio19	239	4.56E-11	10.34128	0.00000	10.02601	0.32339	0.48387	0.15553	0.30793	237	0.00130	47.68634	0.16750
Root-to-tip length	MED_bio24	239	4.9E-11	10.30966	0.00000	10.02242	0.32290	0.51613	0.15553	0.30811	237	0.00130	47.51491	0.16700
Root-to-tip length	MED_bio09	239	1.12E-10	9.95094	0.00000	9.69003	0.31733	0.54839	0.15553	0.31022	237	0.00131	45.57821	0.16129
Root-to-tip length	MED_bio03	239	3.03E-09	8.51799	0.00000	8.28190	0.29368	0.58065	0.15553	0.31879	237	0.00135	37.98929	0.13815
Root-to-tip length	MED_bio17	239	5.65E-07	6.24795	0.00000	6.03534	0.25039	0.61290	0.15553	0.33274	237	0.00140	26.45564	0.10042
Root-to-tip length	MED_bio14	239	4.12E-06	5.38486	0.00001	5.19453	0.23140	0.64516	0.15553	0.33816	237	0.00143	22.23352	0.08577
Root-to-tip length	MED_bioSR1	239	6.45E-06	5.19077	0.00001	5.02163	0.22689	0.67742	0.15553	0.33939	237	0.00143	21.29709	0.08245
Root-to-tip length	MED_Hoursabove_4Cand100W	232	0.000119	3.92615	0.00016	3.79651	0.19709	0.74194	0.15581	0.33500	230	0.00146	15.33763	0.06252
Root-to-tip length	MED_bio12	239	0.000103	3.98869	0.00014	3.83975	0.19643	0.70968	0.15553	0.34702	237	0.00146	15.61193	0.06180
Root-to-tip length	MED_bio04	239	0.007874	2.10382	0.01017	1.99267	0.13553	0.77419	0.15553	0.35900	237	0.00151	7.18358	0.02942
Root-to-tip length	MED_bio16	239	0.01475	1.83119	0.01829	1.73777	0.12450	0.80645	0.15553	0.36070	237	0.00152	6.03410	0.02483
Root-to-tip length	MED_bio25	239	0.031005	1.50856	0.03697	1.43217	0.11028	0.83871	0.15553	0.36268	237	0.00153	4.70864	0.01948
Root-to-tip length	MED_bio26	239	0.046769	1.33004	0.05370	1.27004	0.10174	0.87097	0.15553	0.36375	237	0.00153	3.99531	0.01658
Root-to-tip length	MED_bio13	239	0.048982	1.30996	0.05423	1.26576	0.10074	0.90323	0.15553	0.36387	237	0.00154	3.91614	0.01626
Root-to-tip length	MED_bioSR2	239	0.177178	0.75159	0.18940	0.72263	0.06920	0.93548	0.15553	0.36705	237	0.00155	1.83202	0.00767
Root-to-tip length	MED_bio18	239	0.331013	0.48016	0.34205	0.46591	0.04990	0.96774	0.15553	0.36841	237	0.00155	0.94882	0.00399
Root-to-tip length	MED_bio07	239	0.625	0.20412	0.62500	0.20412	0.02511	1.00000	0.15553	0.36951	237	0.00156	0.23953	0.00101

Supplementary Table 6. Results of single-predictor phylogenetic regressions (single and polynomial) for T_{pref}, IWL, body size and root-to-tip path. Based on its lowest AIC values in most analyses, all analyses were done under the fixed-root Ornstein-Uhlenbeck model. The bioclimatic variable (hours >30°C) represents the median of all occurrence points of a species. Statistically significant P-values are marked in boldface and red font. Asterisks mark values that remain significant after Bonferroni adjustment (separately for the 24 comparisons each in Lacertidae, Lacertini, Eremiadini).

Regression type	dep. variable	indep. variable	AIC	P-value variable	P-value var^2	Estimate intercept	Estimate variable	Estimate var^2	StdErr intercept	StdErr variable	StdErr var^2	t.value intercept	t.value variable	t.value var^2	P-value intercept
**Lacertidae**															
simple	IWL	MHA_30	144.2988	**0.0213**	NA	0.6570	-0.0005	NA	0.2855	0.0002	NA	2.3011	-2.3781	NA	0.0257
polynomial	IWL	MHA_30	143.5095	0.0190	0.1063	1.2197	-0.0013	0.0000	0.4393	0.0005	0.0000	2.7763	-2.4285	1.6461	0.0078
simple	IWL	SVL	149.7787	0.9577	NA	0.1537	-0.0240	NA	1.9642	0.4503	NA	0.0782	-0.0533	NA	0.9379
polynomial	IWL	SVL	151.4178	0.5600	0.5614	9.4727	-4.1275	0.4486	16.0593	7.0320	0.7671	0.5899	-0.5870	0.5848	0.5581
simple	IWL	Tpref	96.2369	0.2089	NA	4.6142	-0.1255	NA	3.4689	0.0980	NA	1.3302	-1.2816	NA	0.1926
polynomial	IWL	Tpref	97.7141	0.5226	0.4927	-28.8017	1.7232	-0.0255	48.2778	2.6657	0.0368	-0.5966	0.6464	-0.6940	0.5550
simple	Root_tip	IWL	-290.4671	0.1270	NA	0.1376	-0.0023	NA	0.0134	0.0015	NA	10.2724	-1.5524	NA	0.0000
polynomial	Root_tip	IWL	-290.8208	0.0345	0.1387	0.1349	-0.0043	0.0015	0.0133	0.0020	0.0010	10.1053	-2.1762	1.5057	0.0000
simple	Root_tip	MHA_30	-1451.1588	**0.0000***	NA	0.1320	0.0000	NA	0.0109	0.0000	NA	12.0556	4.3192	NA	0.0000
polynomial	Root_tip	MHA_30	-1449.5921	0.0369	0.5136	0.1304	0.0000	0.0000	0.0112	0.0000	0.0000	11.6138	2.0987	-0.6542	0.0000
simple	Root_tip	Tpref	-329.5752	**0.0124**	NA	0.0320	0.0031	NA	0.0437	0.0012	NA	0.7329	2.5827	NA	0.4667
polynomial	Root_tip	Tpref	-329.0176	0.2782	0.2443	0.8180	-0.0413	0.0006	0.6693	0.0377	0.0005	1.2222	-1.0953	1.1768	0.2269
simple	SVL	MHA_30	-25.0433	0.0589	NA	4.4240	0.0000	NA	0.1543	0.0000	NA	28.6799	-1.8987	NA	0.0000
polynomial	SVL	MHA_30	-23.2334	0.8190	0.6645	4.3999	0.0000	0.0000	0.1628	0.0001	0.0000	27.0190	-0.2291	-0.4342	0.0000
simple	SVL	Root_tip	107.3312	0.0025	NA	4.7329	-2.7990	NA	0.1511	0.9144	NA	31.3204	-3.0610	NA	0.0000
polynomial	SVL	Root_tip	97.5803	0.0001	**0.0006***	6.4356	-24.0235	62.8472	0.5114	6.1758	18.0673	12.5836	-3.8899	3.4785	0.0000
simple	SVL	Tpref	15.6767	0.7861	NA	4.5662	-0.0068	NA	0.8941	0.0248	NA	5.1072	-0.2726	NA	0.0000
polynomial	SVL	Tpref	16.7408	0.3420	0.3469	16.7704	-0.6920	0.0096	12.8715	0.7219	0.0101	1.3029	-0.9584	0.9488	0.1980
simple	Tpref	IWL	134.7789	0.2089	NA	35.4424	-0.3776	NA	0.2627	0.2947	NA	134.9412	-1.2816	NA	0.0000
polynomial	Tpref	IWL	136.2536	0.7011	0.4300	35.7049	0.1241	0.1361	0.6556	0.3204	0.1703	54.4598	0.3874	0.7993	0.0000
simple	Tpref	MHA_30	202.9288	**0.0000***	NA	34.2977	0.0009	NA	0.3618	0.0002	NA	94.8066	4.4061	NA	0.0000
polynomial	Tpref	MHA_30	203.3536	0.0161	0.2237	33.7379	0.0018	0.0000	0.5802	0.0007	0.0000	58.1494	2.4826	-1.2306	0.0000
simple	Range	SVL	1149.6758	0.4702	NA	13.0483	-0.3910	NA	2.3366	0.5405	NA	5.5844	-0.7234	NA	0.0000
polynomial	Range	SVL	1146.4349	0.0272	**0.0231**	-22.5296	15.1091	-1.6708	15.7254	6.7975	0.7305	-1.4327	2.2227	-2.2874	0.1533

simple	Range	H4C100W	1069.2508	0.0756	NA	40.5350	-3.5496	NA	16.2435	1.9886	NA	2.4955	-1.7850	NA	0.0133
polynomial	Range	H4C100W	1067.3336	0.0480	0.0496	2024.1235	-492.9853	30.1859	1004.7225	247.8837	15.2877	2.0146	-1.9888	1.9745	0.0452
simple	Range	MHA_30	1061.5766	0.0010*	NA	10.5578	0.0005	NA	0.3419	0.0002	NA	30.8773	3.3218	NA	0.0000
polynomial	Range	MHA_30	1063.3935	0.6566	0.6711	10.7860	0.0003	0.0000	0.6367	0.0006	0.0000	16.9406	0.4452	0.4251	0.0000
simple	Range	Tpref	235.7911	0.3982	NA	17.3412	-0.1397	NA	5.9037	0.1641	NA	2.9373	-0.8514	NA	0.0048
polynomial	Range	Tpref	236.9524	0.3900	0.3740	-58.0063	4.0756	-0.0588	84.1950	4.7039	0.0656	-0.6890	0.8664	-0.8962	0.4937
simple	IWL	Tb	84.13721	0.0058	NA	7.1150	-0.2163	NA	2.3781	0.0736	NA	2.9919	-2.9381	NA	0.0051
polynomial	IWL	Tb	85.38698	0.3381	0.4098	28.6829	-1.5236	0.0198	25.9545	1.5682	0.0237	1.1051	-0.9716	0.8345	0.2769
simple	IWL	H4C100W	140.3079	0.0025	NA	38.6525	-4.7323	NA	12.0850	1.4817	NA	3.1984	-3.1938	NA	0.0024
polynomial	IWL	H4C100W	142.1725	0.7101	0.7225	448.7497	-105.9722	6.2473	1148.1659	283.4333	17.4897	0.3908	-0.3739	0.3572	0.6976
simple	Tb	SVL	422.9240	0.1611	NA	37.6161	-0.7615	NA	2.5439	0.5390	NA	14.7868	-1.4128	NA	0.0000
polynomial	Tb	SVL	422.5266	0.1697	0.1273	17.1370	7.2286	-0.7521	13.5554	5.2232	0.4887	1.2642	1.3839	-1.5388	0.2093
simple	Tb	H4C100W	408.2302	0.3178	NA	8.0365	3.2020	NA	26.1007	3.1874	NA	0.3079	1.0046	NA	0.7589
polynomial	Tb	H4C100W	409.7622	0.4778	0.4722	1095.2872	-266.0649	16.6673	1507.9615	373.2069	23.0871	0.7263	-0.7129	0.7219	0.4696
simple	Tb	MHA_30	400.3056	0.0020*	NA	32.4121	0.0010	NA	0.8869	0.0003	NA	36.5437	3.1880	NA	0.0000
polynomial	Tb	MHA_30	402.2837	0.3479	0.8810	32.5350	0.0009	0.0000	1.1909	0.0009	0.0000	27.3202	0.9436	0.1502	0.0000
simple	Tpref	Tb	171.2352	0.0000*	NA	24.9201	0.3226	NA	2.2967	0.0688	NA	10.8504	4.6912	NA	0.0000
polynomial	Tpref	Tb	172.9792	0.7612	0.6258	39.3991	-0.5339	0.0126	29.5860	1.7461	0.0256	1.3317	-0.3058	0.4909	0.1895
simple	Tpref	H4C100W	210.6254	0.8702	NA	32.6527	0.3965	NA	19.7933	2.4154	NA	1.6497	0.1642	NA	0.1046
polynomial	Tpref	H4C100W	207.7731	0.0161	0.0161	-3076.5964	769.8789	-47.5970	1253.5671	310.0023	19.1618	-2.4543	2.4835	-2.4839	0.0173
simple	Tpref	R_Bio01	210.5899	0.8034	NA	35.8094	0.0091	NA	0.5664	0.0363	NA	63.2251	0.2501	NA	0.0000
polynomial	Tpref	R_Bio01	211.8652	0.4686	0.4092	36.2051	-0.0960	0.0053	0.7416	0.1315	0.0063	48.8222	-0.7298	0.8316	0.0000
simple	Tpref	R_H4C100W	209.9093	0.3487	NA	35.7140	0.0004	NA	0.5346	0.0004	NA	66.8095	0.9450	NA	0.0000
polynomial	Tpref	R_H4C100W	211.8198	0.6030	0.7716	35.6179	0.0008	0.0000	0.6296	0.0016	0.0000	56.5700	0.5231	-0.2918	0.0000

**Lacertini**															
simple	IWL	MHA_30	115.2477	0.0703	NA	0.6837	-0.0004	NA	0.2819	0.0002	NA	2.4253	-1.8583	NA	0.0198
polynomial	IWL	MHA_30	116.4569	0.1311	0.3939	0.9562	-0.0008	0.0000	0.4234	0.0005	0.0000	2.2585	-1.5415	0.8618	0.0294
simple	IWL	SVL	118.6606	0.9686	NA	0.2949	-0.0183	NA	2.0124	0.4610	NA	0.1466	-0.0396	NA	0.8842
polynomial	IWL	SVL	120.2165	0.5214	0.5221	11.1442	-4.7388	0.5095	16.9235	7.3248	0.7890	0.6585	-0.6470	0.6458	0.5140
simple	IWL	Tpref	79.4101	0.9583	NA	0.5386	-0.0068	NA	4.5183	0.1285	NA	0.1192	-0.0528	NA	0.9060
polynomial	IWL	Tpref	81.2962	0.7872	0.7867	-42.0871	2.4161	-0.0344	155.7550	8.8564	0.1258	-0.2702	0.2728	-0.2734	0.7891
simple	Root_tip	IWL	-274.4206	0.3575	NA	0.1257	-0.0011	NA	0.0050	0.0012	NA	25.3330	-0.9306	NA	0.0000
polynomial	Root_tip	IWL	-272.4710	0.4382	0.8297	0.1256	-0.0014	0.0002	0.0050	0.0018	0.0009	24.8823	-0.7830	0.2165	0.0000
simple	Root_tip	MHA_30	-715.3790	0.0035	NA	0.1189	0.0000	NA	0.0043	0.0000	NA	27.4599	2.9913	NA	0.0000
polynomial	Root_tip	MHA_30	-715.5167	0.9393	0.1508	0.1215	0.0000	0.0000	0.0047	0.0000	0.0000	26.0844	-0.0764	1.4479	0.0000

simple	Root_ tip	Tpref	-205.8109	0.0837	NA	0.0522	0.0021	NA	0.0410	0.0012	NA	1.2736	1.7873	NA	0.2123
polynomial	Root_ tip	Tpref	-204.7491	0.3702	0.3597	1.7206	-0.0929	0.0013	1.7941	0.1021	0.0015	0.9590	-0.9098	0.9301	0.3452
simple	SVL	MHA_30	-49.6962	0.1612	NA	4.4073	-0.0001	NA	0.1143	0.0000	NA	38.5619	-1.4117	NA	0.0000
polynomial	SVL	MHA_30	-47.8244	0.3689	0.7244	4.4245	-0.0001	0.0000	0.1250	0.0001	0.0000	35.3858	-0.9027	0.3536	0.0000
simple	SVL	Root_tip	-54.9166	0.0497	NA	4.9684	-5.0504	NA	0.3321	2.5436	NA	14.9601	-1.9856	NA	0.0000
polynomial	SVL	Root_tip	-53.1627	0.7885	0.6257	4.2194	6.2543	-41.8034	1.5669	23.2493	85.4452	2.6928	0.2690	-0.4892	0.0083
simple	SVL	Tpref	11.3024	0.7201	NA	4.7798	-0.0113	NA	1.1016	0.0312	NA	4.3390	-0.3616	NA	0.0001
polynomial	SVL	Tpref	7.4025	0.0214	0.0216	112.9624	-6.1699	0.0875	44.6537	2.5415	0.0361	2.5297	-2.4277	2.4234	0.0169
simple	Tpref	IWL	102.8353	0.9583	NA	35.1453	-0.0152	NA	0.2541	0.2882	NA	138.3351	-0.0528	NA	0.0000
polynomial	Tpref	IWL	104.8008	0.9393	0.8618	35.1689	0.0300	-0.0479	0.2916	0.3903	0.2725	120.5954	0.0769	-0.1758	0.0000
simple	Tpref	MHA_30	111.0943	0.0992	NA	34.5418	0.0005	NA	0.3862	0.0003	NA	89.4397	1.6999	NA	0.0000
polynomial	Tpref	MHA_30	113.0914	0.4841	0.9597	34.5195	0.0005	0.0000	0.5885	0.0008	0.0000	58.6613	0.7086	-0.0509	0.0000
simple	Range	SVL	485.8233	0.0010*		-1.7290	2.8238		3.6391	0.8352		-0.4751	3.3811		0.6357
polynomial	Range	SVL	487.7229	0.8865	0.7554	10.1423	-2.3914	0.5689	38.1776	16.7158	1.8212	0.2657	-0.1431	0.3124	0.7910
simple	Range	H4C100W	435.4312	0.0001*		111.4350	-12.3467		23.9812	2.9427		4.6468	-4.1957		0.0000
polynomial	Range	H4C100W	436.7046	0.3913	0.3765	-1536.6291	395.8969	-25.2769	1856.8747	459.7206	28.4504	-0.8275	0.8612	-0.8885	0.4100
simple	Range	MHA_30	449.4244	0.1855		11.5811	-0.0006		0.6920	0.0004		16.7361	-1.3333		0.0000
polynomial	Range	MHA_30	442.7109	0.0012	0.0036	13.5461	-0.0037	0.0000	0.9359	0.0011	0.0000	14.4738	-3.3336	2.9892	0.0000
simple	Range	Tpref	135.2300	0.3673		19.4631	-0.2073		7.9706	0.2265		2.4419	-0.9150		0.0205
polynomial	Range	Tpref	137.1977	0.8736	0.8648	-35.9933	2.9493	-0.0449	323.0513	18.3818	0.2612	-0.1114	0.1604	-0.1718	0.9120
simple	IWL	SVL	118.6606	0.9686		0.2949	-0.0183		2.0124	0.4610		0.1466	-0.0396		0.8842
polynomial	IWL	SVL	120.2165	0.5214	0.5221	11.1442	-4.7388	0.5095	16.9235	7.3248	0.7890	0.6585	-0.6470	0.6458	0.5140
simple	IWL	H4C100W	110.9785	0.0069		34.0562	-4.1548		11.8863	1.4595		2.8652	-2.8467		0.0065
polynomial	IWL	H4C100W	112.9102	0.7932	0.8022	393.7003	-93.0595	5.4935	1426.4927	352.6220	21.7887	0.2760	-0.2639	0.2521	0.7840
simple	Tb	SVL	177.3172	0.7546		33.1990	-0.2863		4.0378	0.9103		8.2221	-0.3145		0.0000
polynomial	Tb	SVL	177.4051	0.1870	0.1805	-16.1487	20.8507	-2.2455	36.4675	15.5562	1.6502	-0.4428	1.3403	-1.3607	0.6601
simple	Tb	H4C100W	171.7656	0.5712		17.8470	1.7278		24.6579	3.0279		0.7238	0.5706		0.4731
polynomial	Tb	H4C100W	173.1510	0.4375	0.4359	2327.9501	-569.2273	35.2735	2939.1834	726.1026	44.8390	0.7920	-0.7839	0.7867	0.4328
simple	Tb	MHA_30	171.9672	0.7543		31.7734	0.0001		0.5650	0.0004		56.2358	0.3150		0.0000
polynomial	Tb	MHA_30	172.8604	0.2376	0.2731	31.1549	0.0011	0.0000	0.7369	0.0009	0.0000	42.2766	1.1982	-1.1105	0.0000
simple	Tpref	Tb	92.4712	0.1868		28.4600	0.2067		4.8406	0.1523		5.8794	1.3575		0.0000
polynomial	Tpref	Tb	94.3436	0.7088	0.7389	1.5926	1.9100	-0.0269	79.8327	5.0539	0.0799	0.0199	0.3779	-0.3372	0.9842
simple	Tpref	H4C100W	114.0345	0.9781		35.6328	-0.0661		19.4504	2.3945		1.8320	-0.0276		0.0766
polynomial	Tpref	H4C100W	115.2310	0.2444	0.2445	-2255.8845	566.4178	-35.0051	1929.3458	477.0762	29.4887	-1.1692	1.1873	-1.1871	0.2515
simple	Tpref	Range_Bio01	113.3593	0.4292		35.5034	-0.0424		0.5523	0.0529		64.2876	-0.8010		0.0000
polynomial	Tpref	Range_Bio01	115.2647	0.9106	0.7712	35.2058	0.0277	-0.0035	1.1591	0.2449	0.0118	30.3743	0.1133	-0.2934	0.0000
simple	Tpref	Range_H4C10 0W	113.8346	0.6667		35.2323	-0.0002		0.3811	0.0005		92.4409	-0.4349		0.0000

polynomial	Tpref	Range_H4C10 0W	115.2903	0.5830	0.4854	34.8766	0.0010	0.0000	0.6334	0.0019	0.0000	55.0585	0.5550	-0.7064	0.0000
**Eremiadini**																
simple polynomial	IWL	MHA_30	25.5685	0.9063	NA	-0.8660	0.0001	NA	1.7778	0.0008	NA	-0.4871	0.1253	NA	0.6516
polynomial	IWL	MHA_30	17.8961	0.0353	**0.0335**	8.8290	-0.0098	0.0000	2.7827	0.0027	0.0000	3.1728	-3.6568	3.7319	0.0504
simple	IWL	SVL	25.3475	0.7042	NA	2.6546	-0.8051	NA	8.1508	1.9738	NA	0.3257	-0.4079	NA	0.7610
polynomial	IWL	SVL	27.3102	0.8941	0.8999	31.5639	-14.5483	1.6256	211.5493	100.4953	11.8841	0.1492	-0.1448	0.1368	0.8909
simple	IWL	Tpref	16.6409	0.3418	NA	9.5828	-0.2674	NA	8.0436	0.2163	NA	1.1914	-1.2364	NA	0.3557
polynomial	IWL	Tpref	15.0269	0.4307	0.4392	188.3973	-9.7662	0.1256	147.7507	7.8416	0.1037	1.2751	-1.2454	1.2117	0.4234
simple	Root_ tip	IWL	-22.2689	0.0436	NA	0.1530	-0.0267	NA	0.0135	0.0092	NA	11.3468	-2.9108	NA	0.0003
polynomial	Root_ tip	IWL	-32.9211	0.0036	**0.0182**	0.1697	-0.0553	-0.0189	0.0059	0.0066	0.0040	28.7770	-8.3566	-4.7056	0.0001
simple	Root_ tip	MHA_30	-682.8643	**0.0048**	NA	0.1541	0.0000	NA	0.0070	0.0000	NA	21.9201	2.8778	NA	0.0000
polynomial	Root_ tip	MHA_30	-682.4814	0.0608	0.2102	0.1462	0.0000	0.0000	0.0094	0.0000	0.0000	15.4910	1.8934	-1.2599	0.0000
simple	Root_ tip	Tpref	-113.4263	0.0588	NA	-0.0561	0.0061	NA	0.1117	0.0030	NA	-0.5025	2.0042	NA	0.6208
polynomial	Root_ tip	Tpref	-112.2579	0.3690	0.4029	-1.5523	0.0862	-0.0011	1.7523	0.0937	0.0013	-0.8859	0.9202	-0.8556	0.3868
simple	SVL	MHA_30	-8.0179	0.1016	NA	4.3161	0.0000	NA	0.0792	0.0000	NA	54.4729	-1.6502	NA	0.0000
polynomial	SVL	MHA_30	-7.0981	0.5318	0.3059	4.2012	0.0001	0.0000	0.1369	0.0001	0.0000	30.6876	0.6271	-1.0285	0.0000
simple	SVL	Root_tip	-9.0491	0.4304	NA	4.3762	-0.9256	NA	0.2061	1.1699	NA	21.2329	-0.7912	NA	0.0000
polynomial	SVL	Root_tip	-9.4494	0.0971	0.1188	5.5617	-14.0410	35.0524	0.7749	8.3976	22.3114	7.1775	-1.6720	1.5711	0.0000
simple	SVL	Tpref	-3.7009	0.3872	NA	3.0660	0.0311	NA	1.2932	0.0351	NA	2.3709	0.8841	NA	0.0279
polynomial	SVL	Tpref	-3.7220	0.2011	0.1914	29.8234	-1.4005	0.0191	19.8007	1.0576	0.0141	1.5062	-1.3243	1.3547	0.1485
simple	Tpref	IWL	23.8467	0.3418	NA	36.5627	-1.6200	NA	1.3189	1.3103	NA	27.7212	-1.2364	NA	0.0013
polynomial	Tpref	IWL	21.5819	0.2934	0.3992	38.4689	-3.4212	-2.4901	1.7624	1.6988	1.8044	21.8280	-2.0139	-1.3800	0.0291
simple	Tpref	MHA_30	74.8275	0.4036	NA	35.9220	0.0003	NA	0.9638	0.0004	NA	37.2703	0.8534	NA	0.0000
polynomial	Tpref	MHA_30	73.8135	0.0953	0.1113	29.8073	0.0062	0.0000	3.7756	0.0035	0.0000	7.8947	1.7553	-1.6703	0.0000
simple	Range	SVL	565.4180	0.2392		7.7415	1.0812		3.8744	0.9139		1.9981	1.1831		0.0480
polynomial	Range	SVL	565.2586	0.1328	0.1477	-50.6532	28.1356	-3.1211	40.2511	18.5850	2.1414	-1.2584	1.5139	-1.4575	0.2108
simple	Range	H4C100W	539.6891	0.0916		48.7408	-4.4409		21.3726	2.6099		2.2805	-1.7015		0.0245
polynomial	Range	H4C100W	541.5365	0.6886	0.7005	478.9781	-110.5975	6.5468	1115.8047	275.2749	16.9759	0.4293	-0.4018	0.3857	0.6686
simple	Range	MHA_30	541.3875	0.2764		11.8195	0.0002		0.5598	0.0002		21.1124	1.0937		0.0000
polynomial	Range	MHA_30	543.2807	0.5871	0.7476	11.4679	0.0005	0.0000	1.2265	0.0010	0.0000	9.3499	0.5446	-0.3225	0.0000
simple	Range	Tpref	90.4482	0.5906		18.4939	-0.1614		10.8651	0.2952		1.7021	-0.5468		0.1042
polynomial	Range	Tpref	83.0504	0.0051	**0.0049**	-429.4082	23.7928	-0.3198	140.9348	7.5269	0.1004	-3.0469	3.1610	-3.1838	0.0066
simple	IWL	H4C100W	25.5108	0.8269		-20.8421	2.4525		86.4571	10.5055		-0.2411	0.2334		0.8214

polynomial	IWL	H4C100W	26.2088	0.4569	0.4565	9671.4578	-2360.0660	143.9613	11367.5049	2770.7885	168.8381	0.8508	-0.8518	0.8527	0.4574
simple	Tb	SVL	198.8719	0.4107		28.5237	1.6742		8.6042	2.0125		3.3151	0.8319		0.0020
polynomial	Tb	SVL	200.5873	0.6250	0.6083	105.7303	-34.5472	4.2364	149.5454	70.0821	8.1949	0.7070	-0.4930	0.5170	0.4840
simple	Tb	H4C100W	197.9612	0.2011		-26.7976	7.6234		47.9932	5.8593		-0.5584	1.3011		0.5799
polynomial	Tb	H4C100W	198.9706	0.3474	0.3398	1870.6905	-462.9099	29.1602	1960.9519	486.3921	30.1545	0.9540	-0.9517	0.9670	0.3463
simple	Tb	MHA_30	192.5431	**0.0091**		32.5958	0.0014		1.2679	0.0005		25.7094	2.7506		0.0000
polynomial	Tb	MHA_30	193.7703	0.1730	0.4003	30.2365	0.0034	0.0000	3.0401	0.0024	0.0000	9.9457	1.3894	-0.8510	0.0000
simple	Tpref	Tb	67.5328	0.5033		33.8906	0.0802		4.1506	0.1172		8.1652	0.6838		0.0000
polynomial	Tpref	Tb	68.6078	0.3983	0.3849	83.5281	-2.7888	0.0412	55.7149	3.2133	0.0461	1.4992	-0.8679	0.8934	0.1533
simple	Tpref	H4C100W	65.9202	0.0034		234.6488	-24.0776		59.5131	7.2353		3.9428	-3.3278		0.0008
polynomial	Tpref	H4C100W	64.2050	0.0752	0.0769	15870.9879	-3831.6320	231.7846	8358.4065	2035.2897	123.8971	1.8988	-1.8826	1.8708	0.0729
simple	Tpref	Range_Bio01	75.4405	0.6946		36.8130	-0.0229		0.7483	0.0574		49.1928	-0.3983		0.0000
polynomial	Tpref	Range_Bio01	76.6969	0.3805	0.4290	37.2083	-0.1351	0.0055	0.8995	0.1505	0.0068	41.3661	-0.8979	0.8082	0.0000
simple	Tpref	Range_H4C100W	75.6143	0.9931		36.6029	0.0000		0.7240	0.0015		50.5570	0.0088		0.0000
polynomial	Tpref	Range_H4C100W	77.6048	0.9335	0.9288	36.5676	0.0003	0.0000	0.8384	0.0034	0.0000	43.6142	0.0846	-0.0905	0.0000

Supplementary Table 7. Results of multiple phylogenetic regressions of T_{pref}, IWL, body size, and root-to-tip paths against bioclimatic variables under the fixed-root Ornstein Uhlenbeck model, calculated for all lacertids, and for Lacertini and Eremiadini only. Significant P-values are shown in bold and red font. The table also contains for Lacertidae a multiple regression model of body size and hours >30°C on root-to-tip length which shows that the bioclimatic influence on the molecular substitution rate (root-to-tip lengths) remains significant when including the (non-significant) body size effect in the analysis. Bioclimatic variables are the medians of all occurrence points of a species. For the Lacertini and Eremiadini data sets, some regressions were not calculated due to low sample sizes (numbers of species) available. See Supplementary Methods for an explanation of variables. Statistically significant P-values are marked in boldface and red font.

Dependent variable	Independent variable/intercept	P-value	Estimate	StdErr	t.value	Number of species
Lacertidae						
IWL	(Intercept)	0.302162723	-0.261655206	0.250317147	-1.045294775	243
IWL	bio02	0.386419687	-0.314260228	0.358865988	-0.875703572	243
IWL	bio06	0.087824163	-0.804309726	0.459661759	-1.749786031	243
IWL	bio13	0.895991300	-0.089770964	0.682354057	-0.131560680	243
IWL	bio15	0.964051213	-0.016634965	0.366784579	-0.045353502	243
IWL	bio19	0.670879852	0.172162300	0.402161183	0.428092782	243
IWL	log(hours >4°C and >100W/m^2)	0.694324845	0.125838517	0.317900552	0.395842398	243
IWL	bio25	0.149640062	-0.459484319	0.312776044	-1.469052149	243
IWL	bio24	0.469097613	-0.203743995	0.278756066	-0.730904256	243
IWL	bioSR4	0.802285379	0.119539949	0.474252023	0.252059967	243
IWL	hours >30°C	0.694898722	0.344003691	0.870766746	0.395058370	243
Tpref	(Intercept)	0.005183148	0.445806048	0.152027898	2.932396305	243
Tpref	bio02	0.891730761	-0.033846509	0.247318375	-0.136853999	243
Tpref	bio06	0.054783702	0.628423241	0.319047315	1.969686661	243
Tpref	bio13	0.129360868	-0.666875742	0.431989881	-1.543730006	243
Tpref	bio15	0.617239943	0.099226008	0.197227131	0.503105264	243
Tpref	bio19	**0.006172416**	0.689102178	0.240300072	2.867673620	243
Tpref	log(hours >4°C and >100W/m^2)	**0.001153361**	-0.844576767	0.243973199	-3.461760433	243
Tpref	bio25	0.327462290	0.216777977	0.219067660	0.989548058	243
Tpref	bio24	**0.024698178**	0.510865204	0.220143595	2.320599900	243
Tpref	bioSR4	0.322291724	0.302171433	0.302083341	1.000291614	243

Tpref	hours >30°C	0.179530161	0.767387181	0.563215219	1.362511442	243
Root.to.tip.length	(Intercept)	0.163489155	-0.377041909	0.269684584	-1.398084766	243
Root.to.tip.length	bio02	0.480882211	0.026950386	0.038169040	0.706079748	243
Root.to.tip.length	bio06	0.528914251	-0.032074111	0.050857979	-0.630660347	243
Root.to.tip.length	bio13	**0.018699406**	0.067448954	0.028471950	2.368961493	243
Root.to.tip.length	bio15	0.360587752	-0.020515626	0.022393348	-0.916148242	243
Root.to.tip.length	bio19	0.245670834	-0.026986463	0.023183839	-1.164020431	243
Root.to.tip.length	log(hours >4°C and >100W/m^2)	0.816045568	-0.006950101	0.029840071	-0.232911666	243
Root.to.tip.length	bio25	0.621970081	-0.015456372	0.031303773	-0.493754269	243
Root.to.tip.length	bio24	0.508834610	-0.016816643	0.025413207	-0.661728482	243
Root.to.tip.length	bioSR4	0.163503677	0.060512826	0.043284158	1.398036341	243
Root.to.tip.length	hours >30°C	**0.030871866**	0.096954511	0.044625447	2.172628349	243
Root.to.tip.length	X.Intercept	0.147417	-0.3935	0.2707	-1.4537	243
Root.to.tip.length	Hours >30°C	**0.000042**	0.1089	0.0261	4.1791	243
Root.to.tip.length	log(SVL)	0.198932	-0.0356	0.0276	-1.2883	243

Lacertini

Tpref	(Intercept)	0.296809837	-0.29084058	0.272154892	-1.06865829	105
Tpref	bio02	0.891656204	-0.06306343	0.457662784	-0.13779454	105
Tpref	bio06	0.555059446	-0.35644855	0.594720965	-0.59935427	105
Tpref	bio13	0.453526011	-0.66981214	0.877784078	-0.76307164	105
Tpref	bio15	0.113620216	-0.68310469	0.414575852	-1.64771943	105
Tpref	bio19	**0.002712901**	1.371825883	0.406163058	3.377525002	105
Tpref	log(hours >4°C and >100W/m^2)	**0.007835627**	-1.1597604	0.396453767	-2.92533581	105
Tpref	bio25	0.319987859	0.375844144	0.369389586	1.01747358	105
Tpref	bio24	0.535476603	0.166426037	0.264359583	0.629544181	105
Tpref	bioSR4	0.543702103	0.348222248	0.564569006	0.616793066	105
Tpref	hours >30°C	**0.01943318**	1.549750353	0.614624061	2.521460599	105

Root.to.tip.length	(Intercept)	0.723821377	-0.07993426	0.225508269	-0.35446265	105
Root.to.tip.length	bio02	0.704317589	0.031714466	0.08330407	0.380707279	105
Root.to.tip.length	bio06	0.405255348	-0.09198503	0.110003219	-0.83620305	105
Root.to.tip.length	bio13	**0.015105449**	0.245805651	0.099223129	2.47730194	105
Root.to.tip.length	bio15	0.274626922	-0.06112712	0.055612235	-1.09916683	105
Root.to.tip.length	bio19	0.169285767	-0.07253408	0.052347569	-1.38562456	105
Root.to.tip.length	log(hours >4°C and >100W/m^2)	0.358334215	-0.06145059	0.066557417	-0.92327187	105
Root.to.tip.length	bio25	0.940324039	-0.00602789	0.080294714	-0.075072	105
Root.to.tip.length	bio24	0.24964792	0.056883501	0.049092953	1.158689742	105
Root.to.tip.length	bioSR4	0.13854494	0.154750095	0.103546917	1.494492542	105
Root.to.tip.length	hours >30°C	**0.068346325**	0.157212195	0.085216135	1.844864183	105

Eremiadini

Tpref	(Intercept)	0.080209	0.904769	0.469549	1.926888	124
Tpref	bio02	0.751213	0.147951	0.455108	0.325089	124
Tpref	bio06	0.138698	1.26803	0.794289	1.596435	124
Tpref	bio13	0.78225	0.283492	1.000882	0.283243	124
Tpref	bio15	0.288608	-0.32598	0.292355	-1.11503	124
Tpref	bio19	0.345421	0.454156	0.4607	0.985794	124
Tpref	log(hours >4°C and >100W/m^2)	0.103078	-1.89383	1.06534	-1.77768	124
Tpref	bio25	0.953419	0.033993	0.568831	0.05976	124
Tpref	bio24	0.222178	0.572553	0.442472	1.293986	124
Tpref	bioSR4	0.356922	0.976503	1.015519	0.96158	124
Tpref	hours >30°C	0.439475	-0.64537	0.804594	-0.80211	124

Root.to.tip.length	(Intercept)	0.001507	-0.61471	0.188804	-3.25582	124
Root.to.tip.length	bio02	0.738064	0.021139	0.063051	0.335273	124
Root.to.tip.length	bio06	0.976141	-0.00369	0.123146	-0.02998	124
Root.to.tip.length	bio13	0.358766	0.045847	0.049747	0.921612	124
Root.to.tip.length	bio15	0.323767	-0.04069	0.041047	-0.99123	124

Root.to.tip.length	bio19	0.615849	-0.02753	0.054718	-0.50319	124
Root.to.tip.length	log(hours >4°C and >100W/m^2)	0.952102	-0.00488	0.081042	-0.06021	124
Root.to.tip.length	bio25	0.51394	-0.04585	0.070008	-0.65486	124
Root.to.tip.length	bio24	**0.024406**	-0.13111	0.057443	-2.28241	124
Root.to.tip.length	bioSR4	0.37477	0.093262	0.104645	0.891228	124
Root.to.tip.length	hours >30°C	0.185683	0.139783	0.104953	1.331864	124

Supplementary Table 8. Corrected Akaike Information Criterion (AICc) values for 15 alternative models of species diversification calculated with RPANDA, tested for the subfamily Lacertinae tree, and for the Lacertini and Eremiadini subclades. Note that no analysis was done for the entire family Lacertidae (thus also including the relatively few species of Gallotiinae) because no high-resolution temperature data were available for the ancient period of the Gallotiinae-Lacertinae split. Green-highlighted models had lowest AIC values (or differed by deltaAICc < 2 from the best model).

Model description	AICc	deltaAICc
**Lacertinae**		
speciation varying linearly with time, extinction varying linearly with time	1754.33	0.00
speciation varying linearly with temperature, extinction varying linearly with temperature	1754.48	0.14
speciation varying linearly with temperature, constant extinction	1759.57	5.24
speciation varying linearly with temperature, no extinction	1760.89	6.56
speciation varying exponentially with temperature, no extinction	1769.87	15.54
speciation varying exponentially with temperature, constant extinction	1771.92	17.59
speciation varying exponentially with temperature, extinction varying linearly with temperature	1771.94	17.61
speciation varying linearly with time, no extinction	1776.39	22.05
speciation varying exponentially with time, constant extinction	1777.84	23.50
speciation varying linearly with time, constant extinction	1778.44	24.11
speciation varying exponentially with time, no extinction	1778.53	24.19
speciation varying exponentially with time, constant extinction	1780.58	26.25
constant speciation rate, no extinction	1788.54	34.21
constant speciation rate, constant extinction	1792.63	38.30
constant speciation rate, extinction varying linearly with time	1792.64	38.30
**Lacertini**		
speciation varying linearly with time, extinction varying linearly with time	724.18	0.00
speciation varying linearly with temperature, extinction varying linearly with temperature	730.15	5.97
speciation varying linearly with temperature, constant extinction	731.50	7.32
speciation varying linearly with temperature, no extinction	733.48	9.30
speciation varying exponentially with time, constant extinction	738.47	14.29
speciation varying exponentially with temperature, no extinction	739.29	15.11
speciation varying linearly with time, no extinction	740.32	16.14
speciation varying exponentially with time, no extinction	740.89	16.71

speciation varying exponentially with temperature, constant extinction	741.41	17.23
speciation varying linearly with time, constant extinction	742.44	18.26
speciation varying exponentially with temperature, extinction varying linearly with temperature	742.86	18.68
speciation varying exponentially with time, constant extinction	743.01	18.83
constant speciation rate, no extinction	744.90	20.72
constant speciation rate, constant extinction	749.10	24.92
constant speciation rate, extinction varying linearly with time	749.11	24.93
**Eremiadini**		
speciation varying linearly with temperature, constant extinction	980.90	0.00
speciation varying linearly with temperature, extinction varying linearly with temperature	982.47	1.57
speciation varying linearly with time, extinction varying linearly with time	984.44	3.54
speciation varying exponentially with temperature, no extinction	987.15	6.25
speciation varying exponentially with temperature, extinction varying linearly with temperature	989.22	8.32
speciation varying exponentially with temperature, constant extinction	989.25	8.35
speciation varying exponentially with time, constant extinction	993.07	12.17
speciation varying linearly with time, no extinction	993.49	12.59
speciation varying linearly with time, constant extinction	995.59	14.69
speciation varying exponentially with time, no extinction	997.62	16.72
speciation varying exponentially with time, constant extinction	999.72	18.82
constant speciation rate, no extinction	1012.48	31.58
constant speciation rate, constant extinction	1016.64	35.74
constant speciation rate, extinction varying linearly with time	1016.65	35.75
speciation varying linearly with temperature, no extinction	1031.90	51.00

Supplementary Table 9. Explained variance and factor loadings of the first five principal components (PC1–PC5) from a phylogenetic PCA of the seven least-correlated bioclimatic variables (accounting for 58% of the total variation). PC1 and PC2 were used for disparity through time analyses. Bioclimatic variables were newly computed (yearly hours >30°C) or taken from Worldclim (Hijmans et al. 2005). See Supplementary Methods for an explanation of variables. Statistically significant P-values are marked in boldface and red font. Variables with the highest loadings in each PC (absolute loading values >0.65) are highlighted in bold and red font.

	**PC1**	**PC2**	**PC3**	**PC4**	**PC5**
**Explained variance**	29.41%	19.89%	14.06%	11.04%	7.54%
**Factor loadings:**					
bio02	0.3915	-0.5372	-0.4442	0.3142	0.4456
bio06	**-0.8676**	0.0279	0.3153	-0.2101	-0.1391
bio13	-0.3005	0.3941	0.4246	0.5906	0.2676
bio15	-0.3323	-0.2790	-0.0401	**0.7527**	-0.3920
bio19	-0.2630	**0.7322**	0.0514	0.0651	0.3696
Hours >30°C	**-0.7199**	-0.4880	0.2523	-0.0033	0.0967
bio25	-0.4756	0.1773	**-0.7114**	0.0754	-0.0007
bio24	0.2130	-0.6000	0.5046	-0.0423	0.2951
bioSR4	**-0.6612**	-0.5449	-0.1916	-0.1323	0.0643
Hours >100W/m^2 and >4°C	**-0.7337**	0.0637	-0.2513	-0.1286	0.2710

Supplementary Table 10. Details of alignments; "legacy" refers to the alignment of four mitochondrial genes and one nuclear gene traditionally used in lacertid systematics.

	legacy	AHE	RNAseq	legacy+AHE+RNAseq
**num taxa**	247	74	22	247
**num genes**	5	324	6,269	6,598
**total num seqs present**	898	23517	126841	150.358
**total num seqs absent**	337	459	11077	1,479,348
**missing data***	27.29%	1.91%	8.03%	90.77%
**DNA chars**	527,633	3,983,4834	182665320	219,287,428
**matrix occupancy****	58.32%	96.40%	78.23%	7.94%
**total aln length**	3648	558418	10613355	11175421

* missing data refers to complete sequences that are missing (gaps not included)
** matrix occupancy = proportion of cells containing DNA characters (total cells=alignment length*num taxa); refers to only DNA characters

Supplementary Table 11. Comparison of T_{pref} measurements using two alternative methods for 14 lacertid species. IR refers to hourly measurements of cloacal temperature in a thermal gradient with infrared bulb, for a 12 h period. IC refers to per-minute measurements of body temperature with a thermocouple attached on the venter, with incandescent bulb, for a 2 h period. All measurements were taken from male specimens in the reproductive period. Note that measurements were taken by different teams and from different individual lizards, and differences therefore can only draw a tentative picture of differences between methods. Median values were calculated per individual, and from these median values calculated per species. Note that for 12 out of 14 species, the IC method gives higher $T_{pref,}$ estimates than the IR method, with a median difference of about 1°C between them.

	N (IR)	Min T_{pref} (IR)	Max T_{pref} (IR)	Median T_{pref} (IR)	N (IC)	Min T_{pref} (IC)	Max T_{pref} (IC)	Median T_{pref} (IC)	Difference between median IC-IR
Darevskia praticola	19	25.4	33.2	29.3	11	34.8	36.4	35.6	6.3
Iberolacerta monticola	28	31.245	35.15	33.395	14	31.55	36.12	34.04	0.645
Lacerta schreiberi	8	32.045	35.05	33.5	15	29.7	35.45	34.1	0.6
Podarcis bocagei	62	28.32	34.825	32.2625	2	34.26	36.895	35.5775	3.315
Podarcis guadarramae	49	27.51	35.425	32.885	15	29.5	36	34.6	1.715
Podarcis liolepis	10	30.6	35.5	32.3125	5	32.2	35.4	33.3	0.9875
Podarcis melisellensis	12	28.625	33.45	32.0625	14	34.321	36.885	36.4225	4.36
Podarcis muralis	45	24.9	34.8	32	70	27.9	37.4	34.15	2.15
Podarcis sicula	10	33.55	35.225	34.475	7	34.46	37.215	35.993	1.518
Podarcis vaucheri	5	32.675	34.07	33.225	15	30.65	35.61	34.12	0.895
Psammodromus algirus	8	34.68	36.125	35.1875	25	31.4	37.1	35.25	0.0625
Scelarcis perspicillata	20	30.025	35.55	33.175	12	31.74	36.638	33.905	0.73
Timon lepidus	6	32.6	34.8	33.45	9	28.2	37.13	32.3	-1.15
Zootoca vivipara	6	32.538	34.375	33.569	77	30.7	37.415	34.15	0.581
**Median of differences**									**0.941**

Supplementary Table 12. Average and range of T_{pref} values (calculated from medians per individual) for five lacertid species in which male data were complemented by female data. Note that there is a trend for females to have a lower T_{pref}, but in two species (*Iberolacerta galani* and *Timon lepidus*) the average values of females are higher. The overall large overlap of individual values for males and females justifies the inclusion of this limited number of female values in order to increase sample sizes and avoid an important influence of possible outliers.

Species	**N females**	**N males**	**T_{pref} females**	**T_{pref} males**
Anatololacerta oertzeni	7	1	35.9 (31.2-37.4)	37.8
Iberolacerta galani	2	2	37.1 (36.4-37.8)	36.5 (36.4-36.6)
Lacerta trilineata	1	1	34.1	36.2
Psammodromus algirus	3	25	33.3 (32.5-34.6)	34.9 (32.9-37.0)
Timon lepidus	1	9	35.9	31.7 (28.3-37.1)

Supplementary Table 13. Lacertid species included in the phylogenetic analysis, and associated analyzed variables. Morph MDS summarizes morphological characteristics, derived from a one-variable multidimensional scaling of the morphological and osteological matrix. IWL is the residual (*1000) from a regression of instantaneous evaporative water loss against body volume (see Materials and Methods for details), assessed for males only, N (IWL) giving the sample size per species. T_{pref} is the preferred body temperature, given as the median of all specimens analyzed per species, sample size given in column N ($T_{pref,}$); data all for males except female data included for 5 species, named F in sample size column. Branch length gives the root-to-tip path for each taxon in the non-ultrametric ML tree, as a proxy for molecular substitution rate. Bio01-Bio27 are the bioclimatic variables from the Worldclim database (Hijmans et al. 2005), given as the median of all localities per species (see Supplementary Methods for an explanation of variables). The two biologically informed climatic variables (yearly hours >30°C, and yearly hours >4°C and >100W/m^2 solar radiation) are also given; they represent medians over all locality records per species.

Clade	Species	MORPH 1MDS-1	IWL	N (IWL)	T_{pref} Median	N (T_{pref})	Body size (max SVL mm)	Root-to-tip length	Root-to-tip-length RNAseq tree	Mean Tb field	Distribution range (GARD)	Hours >30C (median)	Hours > 4°C and >100W (median)
Eremiadini	*Acanthodactylus aegyptius*						52.0	0.22332843		32.7	242805	1832.625	3403.313
Eremiadini	*Acanthodactylus arabicus*						77.0	0.23887304			127002	5356.813	3647.063
Eremiadini	*Acanthodactylus aureus*	-.486					70.4	0.20083288			271937	2188.563	3342.188
Eremiadini	*Acanthodactylus bedriagai*						77.0	0.22961663			192748	1545.156	3692.688
Eremiadini	*Acanthodactylus beershebensis*						90.0	0.2202365		34.1	5380	1801.625	3463.313
Eremiadini	*Acanthodactylus blanci*						95.0	0.20454975			130793	1407.438	3677.188
Eremiadini	*Acanthodactylus blanfordii*						75.0	0.22142724			921917	4329.438	3708.188
Eremiadini	*Acanthodactylus boskianus*	-.408					95.0	0.20359044		36.2	15822326	1863.625	3524.188
Eremiadini	*Acanthodactylus boueti*						63.0	0.21908991			342755	4219.938	3312.063
Eremiadini	*Acanthodactylus busacki*		-1.573272	2	37.12	2	73.6	0.21993904			211576	2055.344	3709.063
Eremiadini	*Acanthodactylus cantoris*						94.0	0.2294946			1418006	4383.000	3709.063
Eremiadini	*Acanthodactylus dumerilii*						68.0	0.21643748			1617810	3712.438	3342.188
Eremiadini	*Acanthodactylus erythrurus*				36.65	9	84.0	0.19505375		33.0	1030082	1287.000	3585.875
Eremiadini	*Acanthodactylus felicis*						65.0	0.20809795			147439	2917.375	3769.938
Eremiadini	*Acanthodactylus gongrorhynchatus*						65.0	0.2369968			132080	4634.188	3647.063
Eremiadini	*Acanthodactylus grandis*						103.0	0.22985838			951179	3304.500	3463.313
Eremiadini	*Acanthodactylus guineensis*						60.0	0.16001706			2113536	4853.188	3465.063
Eremiadini	*Acanthodactylus haasi*						65.0	0.22307389			517700	4537.438	3647.063
Eremiadini	*Acanthodactylus hardyi*						52.0	0.22542983			647411	1923.625	3524.188
Eremiadini	*Acanthodactylus harranensis*						94.8	0.23082186			4831	1956.750	3466.813
Eremiadini	*Acanthodactylus khamirensis*						48.4	0.22808905			10	3910.500	3647.063
Eremiadini	*Acanthodactylus lineomaculatus*	.331	-1.565071	10			94.0	0.19728697			169635	1588.188	3739.188
Eremiadini	*Acanthodactylus longipes*						65.0	0.22390296		34.3	7872081	1925.625	3403.313
Eremiadini	*Acanthodactylus maculatus*						62.0	0.2180956			1194717	1713.750	3646.188

Eremiadini	*Acanthodactylus margaritae*						71.0	0.20951454				1998.969	3709.063
Eremiadini	*Acanthodactylus masirae*						52.0	0.22624029			25057	3952.250	3769.938
Eremiadini	*Acanthodactylus micropholis*						65.0	0.22642793			533436	2842.250	3647.063
Eremiadini	*Acanthodactylus nilsoni*						74.5	0.20271039			778	2569.625	3463.313
Eremiadini	*Acanthodactylus opheodurus*						62.2	0.20672873		33.1	1750982	4045.688	3647.063
Eremiadini	*Acanthodactylus orientalis*						65.0	0.24028582			300436	NA	NA
Eremiadini	*Acanthodactylus pardalis*						77.0	0.21488663		35.3	179337	1695.688	3403.313
Eremiadini	*Acanthodactylus robustus*						77.2	0.26699154			193573	2568.625	3463.313
Eremiadini	*Acanthodactylus savignyi*						75.0	0.21495897			97092	2292.063	3770.188
Eremiadini	*Acanthodactylus schmidti*						105.0	0.22618569		38.4	2040473	4513.750	3647.063
Eremiadini	*Acanthodactylus schreiberi*		-0.892624	16	40.80	18	93.0	0.20413113		38.0	9058	1862.625	3403.313
Eremiadini	*Acanthodactylus scutellatus*						85.0	0.21356932		35.7	9997861	2154.125	3586.625
Eremiadini	*Acanthodactylus senegalensis*						60.0	0.21819007			1531947	3956.750	3587.063
Eremiadini	*Acanthodactylus taghitensis*						55.0	0.21207647			277059	3184.625	3403.313
Eremiadini	*Acanthodactylus tilburyi*						65.0	0.21659358			134696	1923.625	3524.188
Eremiadini	*Acanthodactylus tristrami*						95.0	0.23170984			76448	1649.750	3633.063
Eremiadini	*Adolfus africanus*	-.890					67.0	0.14143305			931945	2974.750	4010.188
Eremiadini	*Adolfus alleni*						70.0	0.13453108			18282	56.500	4010.188
Eremiadini	*Adolfus jacksoni*	-.649					90.0	0.15687793			383063	2639.625	4010.188
Lacertini	*Algyroides fitzingeri*	-.275	-0.572862	6			45.0	0.12380622			33021	1102.000	3647.313
Lacertini	*Algyroides marchi*	-.089	2.880873	5			53.0	0.12430266			3537	1086.500	3557.625
Lacertini	*Algyroides moreoticus*	-.293	0.862242	24			50.0	0.13171996		32.4	22702	1697.375	3647.313
Lacertini	*Algyroides nigropunctatus*	-.058	-1.018624	9	34.43	2	70.0	0.11815045	0.408029572	32.3	75640	1241.000	3467.938
Lacertini	*Anatololacerta anatolica*						75.0	0.1340593		32.9	75665	1194.000	3588.063
Lacertini	*Anatololacerta danfordi*	.571					75.0	0.14008881			58545	1239.500	3527.438
Lacertini	*Anatololacerta oertzeni*		0.548211	1	37.20	7 (6 F)	67.0	0.13812902		31.7	27756	1924.969	3647.313
Lacertini	*Apathya cappadocica*	-.841	0.798744	16	36.68	27	76.0	0.14026014	0.440599369	32.9	324404	1485.500	3496.719
Lacertini	*Apathya yassujica*						58.0	0.11486769			937	1528.594	3635.094
Lacertini	*Archaeolacerta bedriagae*	.439					84.0	0.10746593	0.393405887	32.4	23542	630.000	3647.313
Eremiadini	*Atlantolacerta andreanskyi*	-.132	0.848127	20	34.45	25	55.0	0.10742778		31.4	25900	1117.500	3740.281
Eremiadini	*Australolacerta australis*	-.526					70.0	0.13627401			21772	1542.625	3827.938
Eremiadini	*Congolacerta asukului*	-.007					58.3	0.15606649			1988	2579.625	4010.188
Eremiadini	*Congolacerta vauereselli*	.197					62.0	0.16409625			143329	2457.188	4010.188
Lacertini	*Dalmatolacerta oxycephala*		0.961784	12	33.72	14	70.6	0.11241796	0.400139198	33.1	44449	1348.000	3529.938
Lacertini	*Darevskia caspica*						67.4	0.12539774			14831	1897.875	3346.813
Lacertini	*Darevskia caucasica*						67.0	0.12057487			163631	523.000	2718.250
Lacertini	*Darevskia chlorogaster*						72.0	0.12456733			154653	1652.438	3434.063
Lacertini	*Darevskia clarkorum*						69.0	0.11441562			25500	1163.000	3557.625
Lacertini	*Darevskia daghestanica*	-.099					58.0	0.12073672			91982	980.000	3136.313

Lacertini	*Darevskia defilippii*						58.0	0.11605641			111064	1348.000	3315.813
Lacertini	*Darevskia derjugini*	-.028					65.0	0.12121159			149780	873.000	3032.250
Lacertini	*Darevskia kamii*						67.8	0.12695254			15899	1774.000	3435.813
Lacertini	*Darevskia kopetdaghica*						56.8	0.12158911			6461	1042.000	2955.313
Lacertini	*Darevskia lindholmi*						75.0	0.11861833			25395	982.000	3257.000
Lacertini	*Darevskia mixta*						63.0	0.11587078			43246	1256.000	3436.938
Lacertini	*Darevskia parvula*	-.099					62.3	0.11298913	0.401227291		99316	1148.000	3542.125
Lacertini	*Darevskia portschinskii*						67.0	0.11628546			32134	1225.500	3181.719
Lacertini	*Darevskia praticola*	-.025	1.298221	11	35.20	11	66.0	0.12273315		29.0	227251	1073.000	3210.781
Lacertini	*Darevskia raddei*						76.0	0.12113258			212535	1133.000	3075.438
Lacertini	*Darevskia rudis*		2.252157	10	35.80	9	88.5	0.1139313			250383	873.000	2987.125
Lacertini	*Darevskia saxicola*	-.052					88.0	0.11931438			91925	400.000	2780.250
Lacertini	*Darevskia schaekeli*						56.2	0.12247456			16167	1591.000	3436.375
Lacertini	*Darevskia steineri*						71.0	0.12183415			917	1530.000	3406.094
Lacertini	*Darevskia valentini*		0.445835	10	34.60	10	80.0	0.11541797			264683	644.000	2780.250
Lacertini	*Dinarolacerta montenegrina*						63.8	0.10906643			2501	569.000	3120.844
Lacertini	*Dinarolacerta mosorensis*	.059	0.823090	8	35.79	8	80.0	0.11058562	0.397303669	34.0	33276	279.000	2866.625
Eremiadini	*Eremias argus*	-.506					67.6	0.14962509		37.5	4283180	1380.000	3056.531
Eremiadini	*Eremias arguta*	-.490					100.0	0.13445308		30.2	4694803	1028.000	2871.125
Eremiadini	*Eremias brenchleyi*						69.0	0.15720619			2006898	1302.500	2848.469
Eremiadini	*Eremias grammica*						100.0	0.14474296			1825950	1774.000	3464.625
Eremiadini	*Eremias intermedia*						69.0	0.15671849			1882276	1651.000	3375.813
Eremiadini	*Eremias lalezharica*						71.0	0.15470677				1041.000	3498.375
Eremiadini	*Eremias montana*						59.5	0.15523347			6832	1194.000	3346.125
Eremiadini	*Eremias multiocellata*						78.0	0.13827434		36.6	4684728	583.000	2443.625
Eremiadini	*Eremias papenfussi*						62.0	0.15004904			20	1209.500	3239.563
Eremiadini	*Eremias persica*						98.0	0.14232531			1374595	1650.031	3497.813
Eremiadini	*Eremias pleskei*						60.0	0.13412002		38.8	72410	1470.000	3346.688
Eremiadini	*Eremias przewalskii*						98.0	0.13887547			2395242	891.000	2596.500
Eremiadini	*Eremias strauchi*						80.0	0.15495803		40.3	364825	1424.000	3254.250
Eremiadini	*Eremias stummeri*						67.0	0.137613			34198	829.000	2780.375
Eremiadini	*Eremias suphani*						63.0	0.15200807			20150	1011.000	2989.063
Eremiadini	*Eremias velox*						94.0	0.14408786			4555734	1347.000	3360.813
Eremiadini	*Eremias vermiculata*						71.8	0.1363013			2140780	1104.000	2835.750
Gallotiinae	*Gallotia atlantica*						108.0	0.11729295		33.4	2661	2012.875	3709.063
Gallotiinae	*Gallotia bravoana*						195.0	0.10519081		36.0	112	NA	NA
Gallotiinae	*Gallotia caesaris*	-.817					111.0	0.10816398		35.5	683	1222.750	3709.063
Gallotiinae	*Gallotia galloti*	-1.124	-0.200635	26	35.75	24	145.0	0.1069656	0.394450255	32.6	2861	1131.750	3800.063
Gallotiinae	*Gallotia goliath*						500.0	0.10035653				NA	NA

Gallotiinae	*Gallotia intermedia*						160.0	0.10373754			1251	1496.625	3709.063
Gallotiinae	*Gallotia simonyi*						502.0	0.10397561		34.4	276	NA	NA
Gallotiinae	*Gallotia stehlini*	.794					370.0	0.10050816		33.6	1769	1634.063	3709.063
Eremiadini	*Gastropholis prasina*	.903					110.0	0.14100305			5995	3596.969	4010.188
Eremiadini	*Gastropholis vittata*						109.0	0.13414412			15041	3747.000	3964.688
Eremiadini	*Heliobolus lugubris*				36.20	7	65.0	0.1677637		38.6	2092736	2879.875	3764.188
Eremiadini	*Heliobolus spekii*	-.517					60.0	0.16449881		36.8	1716702	3477.625	4010.188
Lacertini	*Hellenolacerta graeca*	-.852			34.40	16 (IR)	85.0	0.12188417	0.415502292	32.8	23759	1347.000	3588.063
Eremiadini	*Holaspis guentheri*	-.970					53.0	0.16542745	0.47984161		1823996	4580.938	3687.938
Eremiadini	*Holaspis laevis*	-.591	0.247988	6	36.10	6	47.0	0.15789256			558016	3185.906	4010.188
Lacertini	*Iberolacerta aranica*	-.224					66.9	0.11434643		29.2	60	614.000	3467.938
Lacertini	*Iberolacerta aurelioi*						65.3	0.11315053		26.9	122	62.000	3016.000
Lacertini	*Iberolacerta bonnali*	.099			34.48	10	65.4	0.11342378	0.401526777	30.9	24131	232.500	2983.688
Lacertini	*Iberolacerta cyreni*	.132			34.00	106	91.0	0.1136904		28.2	6609	736.000	3467.938
Lacertini	*Iberolacerta galani*	.147	0.624658	2	36.54	4 (2 F)	85.0	0.11380809		30.9	4890	523.000	3345.063
Lacertini	*Iberolacerta horvathi*	.114	0.232573	8			65.0	0.11363006		31.6	48428	476.500	2969.719
Lacertini	*Iberolacerta martinezricai*						69.3	0.11816443			248	950.000	3557.625
Lacertini	*Iberolacerta monticola*	.203	-0.651098	21	34.17	13	84.6	0.11397757		31.5	42969	461.000	3529.938
Eremiadini	*Ichnotropis capensis*	-.412					68.0	0.22498599		38.9	2687195	2849.625	3764.188
Lacertini	*Iranolacerta brandtii*	.190	-0.302480	7	33.90	7	76.4	0.11805877	0.408340966	30.6	99048	1133.000	3195.000
Lacertini	*Iranolacerta zagrosica*						76.0	0.10836708			1638	1225.000	3436.375
Lacertini	*Lacerta agilis*	1.025	0.575152	4	35.00	4	114.0	0.11933691	0.410164326	32.2	11228400	277.000	2950.750
Lacertini	*Lacerta bilineata*	1.040					130.0	0.12257705			815415	704.000	3345.063
Lacertini	*Lacerta media*	.863					160.0	0.12405829			1180712	1284.875	3439.125
Lacertini	*Lacerta pamphylica*						120.0	0.12287434			16078	1468.000	3647.313
Lacertini	*Lacerta schreiberi*	1.055	0.098356	8	34.05	15	135.0	0.11729655		31.1	211098	705.000	3557.625
Lacertini	*Lacerta strigata*	1.077					160.0	0.12063374			435277	1379.000	3316.375
Lacertini	*Lacerta trilineata*	1.117	0.464684	1	34.10	3 (1 F)	174.0	0.12213763		30.3	594928	1348.000	3585.313
Lacertini	*Lacerta viridis*	1.138			36.20	1	160.0	0.12166038		33.9	1371040	996.500	3209.688
Eremiadini	*Latastia longicaudata*	-.208					110.0	0.15373422		37.8	7042622	3370.625	4010.188
Eremiadini	*Meroles anchietae*				36.00	2	55.0	0.17736803		36.8	127659	2698.875	3825.063
Eremiadini	*Meroles ctenodactylus*				35.15	2	97.0	0.1872556		36.9	29449	1784.063	3827.063
Eremiadini	*Meroles cuneirostris*				37.00	10	58.0	0.17816503		35.0	107100	2820.750	3825.063
Eremiadini	*Meroles knoxii*				35.80	6	68.0	0.17973816		31.9	123541	1664.063	3827.063
Eremiadini	*Meroles micropholidotus*				37.70	3	68.0	0.17750896		31.5	32644	2213.625	3704.188
Eremiadini	*Meroles reticulatus*				36.75	4	55.0	0.17306205		34.0	96442	1937.625	3704.188
Eremiadini	*Meroles squamulosus*	-.475			37.30	1	77.0	0.20609649		37.1	3394617	3002.313	3704.188
Eremiadini	*Meroles suborbitalis*				35.15	21	71.0	0.18390412		35.4	651581	2449.125	3827.063
Eremiadini	*Mesalina adramitana*						46.0	0.20514518		37.7	669902	4154.625	3709.063

Eremiadini	*Mesalina bahaeldini*						52.0	0.2116807			16117	1770.625	3493.750
Eremiadini	*Mesalina balfouri*						58.0	0.2104648			3672	3524.938	3769.938
Eremiadini	*Mesalina bernoullii*						56.0	0.20436972				1787.188	3524.188
Eremiadini	*Mesalina brevirostris*						60.0	0.21069373		35.5	1042583	3137.125	3616.625
Eremiadini	*Mesalina guttulata*						70.0	0.20655726		31.3	11286883	1953.625	3524.188
Eremiadini	*Mesalina kuri*						57.0	0.2013925			132	NA	NA
Eremiadini	*Mesalina martini*						50.0	0.1898205			160200	2987.875	3586.188
Eremiadini	*Mesalina microlepis*						55.0	0.21087677				1589.750	3708.188
Eremiadini	*Mesalina olivieri*	-.653					52.0	0.19779189	0.527914622	30.2	2654154	1923.625	3524.188
Eremiadini	*Mesalina pasteuri*						50.0	0.19458852			5182477	4313.188	3419.688
Eremiadini	*Mesalina rubropunctata*						67.0	0.19784166			5196537	2853.250	3464.188
Eremiadini	*Mesalina saudiarabica*						55.0	0.20905751				3322.000	3678.063
Eremiadini	*Mesalina simoni*		-2.361078	10			50.0	0.19607985			61870	1925.625	3770.188
Eremiadini	*Mesalina watsonana*						60.0	0.18874806			2388396	2017.750	3526.063
Eremiadini	*Nucras boulengeri*	.001					65.0	0.17801293			626074	3278.625	4010.188
Eremiadini	*Nucras intertexta*						94.0	0.14974336		38.9	1025498	2529.594	3704.188
Eremiadini	*Nucras lalandii*						120.0	0.15617345			303662	1631.625	3765.063
Eremiadini	*Nucras livida*						85.0	0.15550748			135027	1692.750	3827.938
Eremiadini	*Nucras taeniolata*						111.0	0.15429335			60548	1633.344	3767.063
Eremiadini	*Nucras tessellata*				36.00	1	94.0	0.15144568		36.4	652056	1871.000	3827.063
Eremiadini	*Omanosaura cyanura*	-.344					60.0	0.13118755			23327	3615.469	3709.063
Eremiadini	*Omanosaura jayakari*	-.834					200.0	0.13387883			33771	3419.625	3709.063
Eremiadini	*Ophisops beddomei*						37.0	0.16960019			293246	4652.125	3769.938
Eremiadini	*Ophisops elegans*				37.00	8 (IR)	70.0	0.16816624		31.3	2424527	1558.750	3494.500
Eremiadini	*Ophisops jerdonii*						49.0	0.16469886			2903444	4168.563	3646.188
Eremiadini	*Ophisops leschenaultii*						57.0	0.18791424			1301159	3827.625	3949.313
Eremiadini	*Ophisops microlepis*	-.706					65.0	0.1896773			1022970	NA	NA
Eremiadini	*Ophisops occidentalis*						48.0	0.16888616			376209	1439.000	3739.188
Lacertini	*Parvilacerta fraasii*	-.602					60.0	0.14896476			1419	1147.438	3650.594
Lacertini	*Parvilacerta parva*		-0.403568	10	37.23	10	62.0	0.15607644			388237	950.000	3103.688
Eremiadini	*Pedioplanis burchelli*						62.9	0.17583042			374425	1541.188	3827.938
Eremiadini	*Pedioplanis gaerdesi*				37.00	6	52.0	0.17155759		38.1	66589	2120.500	3764.188
Eremiadini	*Pedioplanis inornata*				37.05	6	53.0	0.17590742		37.2	250734	2790.750	3764.188
Eremiadini	*Pedioplanis laticeps*	.234			37.35	2	63.0	0.18446429			228158	1871.000	3827.063
Eremiadini	*Pedioplanis lineoocellata*				37.55	24	67.0	0.17038097		38.0	1757909	2053.750	3827.938
Eremiadini	*Pedioplanis namaquensis*				37.75	16	56.0	0.16918055		37.4	1296977	2421.438	3827.063
Eremiadini	*Pedioplanis undata*				37.40	13	62.0	0.16882297		39.0	255654	2727.750	3764.188
Eremiadini	*Philochortus spinalis*						58.0	0.20977747			60700	NA	NA
Lacertini	*Phoenicolacerta cyanisparsa*		-0.050486	1	36.50	1	65.0	0.14339484			16067	1834.750	3588.063

Lacertini	*Phoenicolacerta kulzeri*						87.0	0.14136774			43865	1376.750	3770.188
Lacertini	*Phoenicolacerta laevis*		0.938981	16	36.85	15	87.0	0.13802378	0.438033507	30.4	118144	1621.313	3708.188
Lacertini	*Phoenicolacerta troodica*						70.0	0.1373711		33.5	9272	1559.313	3677.188
Lacertini	*Podarcis bocagei*	.443	0.130971	62	35.57	2	70.0	0.11843616		32.3	86329	674.000	3619.625
Lacertini	*Podarcis carbonelli*	.414	-0.591148	23			65.0	0.11887718		33.3	59927	1117.500	3647.313
Lacertini	*Podarcis cretensis*		0.540046	6			71.5	0.10977113		33.2	4546	1742.750	3585.313
Lacertini	*Podarcis erhardii*	.326	-0.889406	10			84.0	0.1144775		32.9	157103	1680.438	3647.313
Lacertini	*Podarcis filfolensis*						86.0	0.11192369		32.8	338	NA	NA
Lacertini	*Podarcis gaigeae*						100.0	0.11680801		32.6	237	1713.000	3647.313
Lacertini	*Podarcis guadarramae*		-0.476823	18	34.55	15	64.0	0.11745126			107594	857.000	3557.625
Lacertini	*Podarcis hispanicus*		-0.363240	5			74.0	0.12327501		31.9	561352	1164.000	3585.313
Lacertini	*Podarcis lilfordi*	.492	-0.406417	10			81.0	0.11742422		32.3	4386	1438.438	3647.313
Lacertini	*Podarcis liolepis*	.586	0.006870	6	33.50	5	80.0	0.11582243	0.409431043	34.2	192255	1102.000	3585.313
Lacertini	*Podarcis melisellensis*		0.199985	25	36.29	14	74.0	0.11713899		32.5	50980	1317.000	3467.938
Lacertini	*Podarcis milensis*						75.0	0.11916226		33.0	243	NA	NA
Lacertini	*Podarcis muralis*	.425	1.356742	16	34.15	67	78.0	0.11597096	0.405819171	32.2	1836392	460.000	2952.938
Lacertini	*Podarcis peloponnesiacus*		0.004669	6			85.0	0.11785326		33.0	21830	1834.313	3647.313
Lacertini	*Podarcis pityusensis*	.499	-0.623577	10			82.0	0.11236131		34.2	741	1529.875	3585.313
Lacertini	*Podarcis raffonei*						85.0	0.11628389			72	NA	NA
Lacertini	*Podarcis siculus*	.572	-0.434002	16	36.00	7	90.0	0.11630001		34.5	275645	1287.000	3647.313
Lacertini	*Podarcis tauricus*		-1.094684	8	36.30	3	91.0	0.12348355		32.8	557946	1073.000	3288.000
Lacertini	*Podarcis tiliguerta*	.507	-0.204186	20			87.0	0.10749194		32.5	32892	1102.000	3647.313
Lacertini	*Podarcis vaucheri*		0.518641	30	33.90	15	60.0	0.11457187		30.6	366422	1346.875	3647.313
Lacertini	*Podarcis virescens*		0.682844	14			63.0	0.12786251			170958	1226.000	3647.313
Lacertini	*Podarcis waglerianus*		-0.759555	10			76.0	0.11549935			23912	1315.938	3647.313
Eremiadini	*Poromera fordii*	-.785					65.0	0.25090509			657793	3883.688	3825.750
Gallotiinae	*Psammodromus algirus*	.303	-1.086538	8	34.90	28 (3 F)	93.0	0.116154	0.408649042	32.8	1201029	1102.000	3587.250
Gallotiinae	*Psammodromus blanci*	-.544					47.0	0.12009276			171701	1408.000	3754.688
Gallotiinae	*Psammodromus edwarsianus*						56.0	0.11823256				1164.000	3647.313
Gallotiinae	*Psammodromus hispanicus*	-.216			32.30	13	57.0	0.11715504		31.4	550490	1010.000	3557.625
Gallotiinae	*Psammodromus microdactylus*						58.0	0.11456557			17040	1619.750	3646.188
Gallotiinae	*Psammodromus occidentalis*						44.0	0.1228475			39944	858.000	3585.313
Eremiadini	*Pseuderemias smithii*						47.0	0.15180043			54007	4721.375	4010.188
Lacertini	*Scelarcis perspicillata*	.567	-0.861013	20	33.85	12	60.0	0.12407782	0.417312466		142585	1527.750	3769.625
Lacertini	*Takydromus amurensis*	-1.049					80.0	0.15810675			1086887	935.500	2893.594
Lacertini	*Takydromus dorsalis*						70.0	0.15527693			529	2443.938	3647.063
Lacertini	*Takydromus formosanus*						64.0	0.16267536			35557	2077.938	3709.063
Lacertini	*Takydromus hsuehshanensis*						72.0	0.15924175			6693	521.000	3709.063
Lacertini	*Takydromus intermedius*						62.0	0.14992491			1043969	2937.063	3586.188

Lacertini	*Takydromus luyeanus*						54.2	0.17343981			4313	1786.406	3693.563
Lacertini	*Takydromus sauteri*						76.5	0.16476557			11519	2638.563	3709.063
Lacertini	*Takydromus septentrionalis*						80.0	0.16006814		30.4	2483103	2021.750	3524.188
Lacertini	*Takydromus sexlineatus*	-1.024	-0.576516	9	36.28	9	70.0	0.17399325	0.490195103	31.5	4479411	3978.875	3826.438
Lacertini	*Takydromus smaragdinus*						65.0	0.15347455			2664	2596.813	3647.063
Lacertini	*Takydromus stejnegeri*						62.0	0.15887451			31225	2413.719	3662.563
Lacertini	*Takydromus sylvaticus*						58.0	0.15187705			15415	1563.000	3495.938
Lacertini	*Takydromus tachydromoides*	-.924					70.0	0.16435843			368570	1318.000	3374.438
Lacertini	*Takydromus toyamai*						54.9	0.16308918			193	NA	NA
Lacertini	*Takydromus viridipunctatus*						59.5	0.16346183			5132	1954.625	3647.063
Lacertini	*Takydromus wolteri*						66.0	0.1666287			1834300	1378.000	3284.688
Lacertini	*Teira dugesii*	-.239					81.0	0.10925595		33.8	3360	1040.875	3770.188
Lacertini	*Timon kurdistanicus*						137.0	0.12726518				1501.000	3436.375
Lacertini	*Timon lepidus*	.940	0.415423	6	32.83	8 (1 F)	260.0	0.12333826		30.4	619422	1195.000	3647.313
Lacertini	*Timon nevadensis*						171.0	0.12381034				1102.000	3618.500
Lacertini	*Timon pater*	1.108					170.0	0.12291479	0.415904427		187074	1437.875	3585.313
Lacertini	*Timon princeps*	.987					148.0	0.122477			230996	1498.531	3559.250
Lacertini	*Timon tangitanus*						137.0	0.12767095			145870	1133.000	3710.938
Eremiadini	*Tropidosaura cottrelli*						66.0	0.15302048			3179	937.188	3643.063
Eremiadini	*Tropidosaura essexi*						52.0	0.15778345			10708	1599.656	3781.063
Eremiadini	*Tropidosaura gularis*	.679					62.0	0.15402059			72717	1267.875	3827.938
Eremiadini	*Tropidosaura montana*						66.0	0.15629632			116401	1267.875	3827.063
Eremiadini	*Vhembelacerta rupicola*	-.169					51.5	0.1764303			15435	2605.438	3704.188
Lacertini	*Zootoca vivipara*	-.683	2.112845	8	34.10	74	77.0	0.12305164	0.415337635	30.5	17422256	0.000	2833.375

Supplementary Table 14. Lacertid species included in the phylogenetic analysis, and associated bioclimatic variables bio01-bio19 from the Worldclim database (Hijmans et al. 2005), given as the median of all localities per species (see Supplementary Methods for an explanation of variables).

Species	bio01	bio02	bio03	bio04	bio05	bio06	bio07	bio08	bio09	bio10	bio11	bio12	bio13	bio14	bio15	bio16	bio17	bio18	bio19
Acanthodactylus aegyptius	20.842	13.517	46.770	557.184	32.700	5.600	28.800	14.867	25.800	26.917	13.950	125	28	0	102.230	76	0	0	76
Acanthodactylus arabicus	30.692	9.383	46.223	422.904	40.800	20.400	20.300	35.600	28.050	36.150	25.917	60	10	2	40.825	21	9	13	14
Acanthodactylus aureus	21.058	9.342	61.667	213.268	29.300	13.600	15.700	23.417	21.433	23.750	18.883	31	9	0	77.264	18	2	11	7
Acanthodactylus bedriagai	15.027	12.004	38.109	716.355	30.100	-0.900	31.500	12.650	24.042	24.042	7.000	347.5	39.5	8	31.019	110.5	40.5	40.5	89.5
Acanthodactylus beershebensis	18.996	13.092	45.984	574.838	31.700	3.500	28.300	11.517	25.317	25.367	11.517	215	50	0	105.728	136	0	0	136
Acanthodactylus blanci	15.658	11.708	37.933	706.123	30.600	-0.200	31.300	12.950	24.750	24.750	7.667	368	43	10	29.450	120	52	52	107
Acanthodactylus blanfordii	26.663	13.942	39.998	785.479	40.650	9.400	35.500	19.058	29.500	33.342	17.267	139	31	1	91.351	83	7	9	79
Acanthodactylus boskianus	20.400	13.092	43.597	593.297	34.500	4.800	29.400	13.783	26.367	27.650	12.733	123	24	0	87.523	61	0	0	42
Acanthodactylus boueti	26.015	10.387	68.618	158.771	32.800	17.700	15.100	25.258	26.567	28.125	24.058	1137	209.5	4	77.763	541	24	183.5	533.5
Acanthodactylus busacki	20.508	9.617	54.915	249.776	28.900	12.500	16.100	18.000	22.517	23.817	17.233	75	18	0	84.480	43	1	3	43
Acanthodactylus cantoris	25.883	15.592	46.615	540.360	40.900	10.400	31.000	29.800	24.283	32.050	19.567	153	53	1	103.510	90	7	39	44
Acanthodactylus dumerilii	24.817	10.183	54.417	274.598	34.500	15.300	18.800	27.733	24.767	28.067	21.200	64	23	0	133.457	50	2	47	4
Acanthodactylus erythrurus	16.256	10.650	39.560	560.723	29.150	3.550	25.800	12.367	23.367	23.592	9.925	422	56.5	4	52.099	153.5	26	36.5	129
Acanthodactylus felicis	23.025	10.117	54.040	292.676	32.100	12.300	18.800	24.617	23.383	26.750	18.750	130	23	3	56.677	46	14	31	32
Acanthodactylus gongrorhynchatus	27.708	15.008	45.117	642.521	42.600	9.800	33.100	23.367	33.483	35.167	19.633	77	21	1	103.046	51	3	4	35
Acanthodactylus grandis	24.625	14.350	36.905	923.779	43.000	2.900	39.800	12.700	33.050	35.283	12.700	242	69	0	105.958	172	0	1	172
Acanthodactylus guineensis	28.235	12.933	64.509	235.024	37.050	16.800	20.000	26.500	25.967	31.458	25.750	804.5	219.5	0	117.455	524	1	91.5	1.5
Acanthodactylus haasi	27.825	14.908	48.412	601.866	42.300	10.700	32.600	25.400	29.033	34.817	20.650	108	21	2	84.558	54	7	11	46
Acanthodactylus hardyi	22.575	14.496	41.188	810.450	39.700	3.200	36.500	13.092	32.008	32.008	12.083	79	16	0	81.972	43.5	0	0	41.5
Acanthodactylus harranensis	17.737	15.375	39.322	862.887	36.600	-2.500	39.100	7.083	27.017	28.350	7.083	352	62	1	79.325	174	4	7	174
Acanthodactylus khamirensis	26.869	9.779	37.830	603.242	39.500	13.650	25.850	19.075	33.042	33.642	19.075	163.5	40	0	116.127	118	1	1	118
Acanthodactylus lineomaculatus	18.117	8.717	49.352	327.500	27.500	9.400	18.100	15.067	21.900	22.233	14.183	295	59	0	83.871	150	3	4	150
Acanthodactylus longipes	21.713	13.033	46.980	563.739	35.800	5.400	28.400	14.817	25.950	28.283	14.050	39	15	0	103.978	31	0	0	11
Acanthodactylus maculatus	18.225	13.108	40.516	720.226	32.800	2.050	32.800	12.992	26.092	26.133	10.675	246.5	31.5	2.5	50.637	84	16.5	16.5	72
Acanthodactylus margaritae	19.431	11.133	53.662	322.824	29.800	9.150	20.650	15.200	22.992	23.450	15.200	179	42.5	0	89.540	105	3	3	105
Acanthodactylus masirae	26.479	9.125	52.019	279.028	34.800	18.000	17.400	24.000	26.700	29.833	22.867	73	17	0	67.380	29	5	25	24
Acanthodactylus micropholis	25.021	14.183	40.689	691.964	38.850	8.050	32.650	16.292	27.492	32.225	15.050	167.5	35	0.5	88.227	94.5	6.5	12	84
Acanthodactylus nilsoni	22.104	15.292	37.388	960.448	41.800	0.900	40.900	11.450	33.800	33.800	10.333	424	82	0	95.388	234	0	0	226
Acanthodactylus opheodurus	26.894	13.608	44.126	638.030	40.750	11.200	30.750	24.050	30.100	33.575	19.717	114.5	25.5	1	79.327	61	5.5	6	49.5
Acanthodactylus orientalis	NA	NA	NA	NA	NA	NA	NA	NA	NA	NA	NA	NA	NA	NA	NA	NA	NA	NA	NA
Acanthodactylus pardalis	19.921	9.958	43.297	484.057	30.600	7.900	22.900	14.200	25.317	25.600	13.983	139	35	0	102.783	93	0	0	72
Acanthodactylus robustus	23.146	15.825	38.283	925.992	42.500	0.700	41.100	10.867	34.300	34.300	10.867	119	23	0	80.329	58	0	0	56
Acanthodactylus savignyi	19.929	7.375	34.302	527.634	30.800	9.300	21.500	15.083	25.983	26.267	13.583	316	75	0	93.890	184	1	11	175
Acanthodactylus schmidti	27.725	15.071	44.990	643.729	42.600	10.000	32.800	23.383	33.675	35.142	19.750	97	29	1	105.051	63	3	4	47
Acanthodactylus schreiberi	19.700	9.742	40.517	521.181	31.700	7.700	24.200	13.400	25.467	25.950	13.167	540	130	0	114.215	347	0	1	347
Acanthodactylus scutellatus	20.862	13.308	42.095	687.232	35.200	4.000	31.700	14.367	27.800	28.217	12.617	81	18	0	65.803	51	0	1	32
Acanthodactylus senegalensis	25.467	12.200	55.868	257.757	34.800	15.600	18.600	27.867	24.767	28.200	22.350	113	48	0	156.678	100	2	90	4
Acanthodactylus taghitensis	22.525	13.850	36.257	878.494	41.200	3.000	38.200	16.900	30.867	33.550	11.867	49	8	1	47.048	22	4	4	16
Acanthodactylus tilburyi	20.987	14.992	42.833	766.346	37.400	2.400	35.000	15.983	28.450	29.850	11.267	17	4	0	59.726	8	0	0	6
Acanthodactylus tristrami	14.831	13.512	42.654	665.825	29.100	-2.100	31.200	6.467	22.225	22.283	6.467	311	72	0	105.307	202	0	0	202
Adolfus africanus	19.606	10.025	89.260	37.913	25.450	13.500	11.850	19.500	19.583	20.083	19.225	1385.5	167.5	45	35.611	461	195.5	299	235.5
Adolfus alleni	6.158	9.854	80.578	76.368	12.100	0.200	12.300	6.450	6.542	6.800	5.208	1576.5	236	51	46.529	564	233.5	348	298.5
Adolfus jacksoni	17.729	10.396	86.169	47.809	24.200	11.650	12.300	18.275	17.433	18.700	17.283	1295	205.5	31	44.078	488	149	318	211.5
Algyroides fitzingeri	14.760	8.012	32.974	594.807	27.000	2.200	24.450	12.067	22.533	22.533	7.967	678.5	101	9.5	49.164	270	51	65	227.5

Algyroides marchi	12.202	12.058	37.624	721.470	28.400	-3.550	32.100	8.000	21.733	21.908	4.208	488.5	60	7.5	43.458	169	42	46	157
Algyroides moreoticus	13.679	8.600	33.366	643.408	26.000	0.600	25.800	8.250	21.600	21.600	6.750	729	120	13	66.088	330	48	48	296
Algyroides nigropunctatus	15.413	8.317	34.211	650.453	27.400	2.400	24.100	13.600	22.900	22.900	8.433	1042	153	14	62.333	436	60	91	312
Anatololacerta anatolica	15.979	8.758	34.491	638.459	28.400	3.400	25.300	8.900	23.700	23.700	8.900	695	142	3	87.963	364	13	13	364
Anatololacerta danfordi	12.813	9.833	33.381	696.246	27.250	-2.150	29.000	5.692	21.483	21.658	4.083	622	109.5	5.5	68.746	296	24.5	29	296
Anatololacerta oertzeni	18.750	8.200	34.433	596.177	30.500	6.600	23.900	12.067	25.717	25.800	12.067	745	153	1	91.692	422	9	10	397
Apathya cappadocica	13.388	12.337	33.503	902.577	30.600	-5.600	37.200	5.117	24.100	24.125	1.950	603	100	3	71.555	284	14	19.5	276.5
Apathya yassujica	12.846	14.171	37.294	893.992	31.300	-8.300	38.600	2.825	23.050	23.817	1.783	246	50	0	89.442	131	5	6.5	128.5
Archaeolacerta bedriagae	10.494	7.212	31.156	603.501	22.350	-1.450	23.350	7.058	18.283	18.292	3.775	881	119	16	45.443	331	78	82	290.5
Atlantolacerta andreanskyi	8.023	8.125	29.332	681.573	21.600	-5.200	26.800	3.683	16.525	16.525	0.275	524	71	6	52.080	194	36	36	176.5
Australolacerta australis	13.952	12.217	51.563	404.381	24.750	1.400	23.900	9.042	18.658	18.783	9.042	409	65	15	51.882	179	48	51	179
Congolacerta asukului	12.408	7.583	83.333	42.095	16.700	7.600	9.100	12.533	11.983	12.733	11.767	1989	267	26	49.697	696	120	633	266
Congolacerta vauereselli	15.529	8.742	89.167	28.640	20.500	10.600	9.800	15.550	15.233	15.767	15.150	1584	218	29	43.832	608	146	402	240
Dalmatolacerta oxycephala	16.404	7.342	30.222	629.727	27.400	4.200	24.450	13.567	24.125	24.125	9.342	838	109.5	27.5	36.491	303	117	117	254
Darevskia caspica	15.692	8.850	31.851	707.284	29.300	2.400	27.600	12.933	21.783	24.233	7.183	536	83	15	48.629	218	58	58	175
Darevskia caucasica	3.912	9.308	29.271	823.926	18.600	-13.000	31.800	11.000	-6.300	13.550	-6.400	771	113	31	46.979	319	95	241	95
Darevskia chlorogaster	15.133	8.833	30.961	732.918	29.050	0.200	28.800	9.842	22.592	24.000	5.425	460	66	11.5	46.404	174	47	47	146
Darevskia clarkorum	12.454	6.525	29.392	594.904	23.400	1.200	22.200	10.250	10.867	19.517	5.267	1714	201	89	28.359	589	288	416	490
Darevskia daghestanica	10.217	9.950	28.841	891.639	26.600	-7.900	34.500	19.100	-0.867	21.083	-0.867	477	65	16	44.679	184	56	172	56
Darevskia defilippii	9.617	10.067	29.389	862.774	25.600	-7.900	33.500	5.183	19.283	19.633	-1.150	322	56	6	51.312	147	21	22	112
Darevskia derjugini	8.123	9.404	31.300	768.050	23.000	-8.700	30.050	9.717	8.408	17.825	-1.717	1143.5	128	73.5	21.830	351	236	251	293
Darevskia kamii	15.321	8.483	30.128	778.781	30.100	1.500	29.500	9.417	24.583	24.583	6.367	376	57	8	50.185	149	31	31	118
Darevskia kopetdaghica	6.213	10.225	28.482	939.762	23.900	-12.000	35.800	1.433	16.733	17.467	-5.617	399	64	13	48.988	163	42	47	122
Darevskia lindholmi	12.050	7.150	26.580	741.631	26.100	-0.500	26.900	5.567	10.167	21.450	3.500	441	56	27	22.343	143	87	107	107
Darevskia mixta	11.417	8.367	32.555	646.530	23.300	-2.400	25.700	5.383	10.133	19.200	3.450	1622	198	98	21.931	521	312	367	490
Darevskia parvula	11.496	6.433	27.850	617.191	22.400	-1.000	23.100	8.733	10.450	18.583	3.567	1474	177	83	26.862	500	260	322	434
Darevskia portschinskii	10.602	10.654	33.670	769.152	26.050	-5.600	31.650	13.825	1.192	19.975	1.192	516	83.5	18.5	48.133	214.5	65.5	157.5	65.5
Darevskia praticola	10.958	9.550	30.629	812.123	26.400	-5.900	31.000	15.367	2.600	21.483	0.683	694	95	35	27.526	241	113	215	131
Darevskia raddei	8.646	10.650	31.553	828.867	24.900	-9.100	33.600	11.933	1.083	19.033	-1.550	483	77	17	46.512	202	62	104	77
Darevskia rudis	6.837	8.967	31.645	777.348	20.100	-7.700	30.100	11.917	0.050	15.117	-0.733	772	105	43	26.093	274	130	186	188
Darevskia saxicola	5.938	9.625	31.682	743.047	20.200	-9.500	29.700	12.750	-2.767	14.983	-3.083	896	104	42	23.169	280	151	277	166
Darevskia schaekeli	10.108	7.917	27.681	764.048	25.800	-5.200	28.600	4.900	20.183	20.467	-0.650	212	38	4	62.736	93	16	21	68
Darevskia steineri	15.135	8.775	29.394	776.771	29.900	0.300	29.850	9.517	24.500	24.500	5.442	318	56.5	4	67.015	148	17	17	117.5
Darevskia valentini	3.700	10.675	29.741	889.829	19.500	-15.500	35.400	7.200	-6.767	14.083	-8.383	610	99	25	42.619	246	85	130	98
Dinarolacerta montenegrina	5.706	9.746	35.184	661.086	19.100	-8.600	27.700	2.542	14.025	14.025	-2.175	1102	130.5	61	19.969	347	209	209	292
Dinarolacerta mosorensis	4.565	8.337	33.165	618.163	17.000	-8.400	25.000	1.733	4.817	12.467	-2.867	1159	125	66.5	18.505	336	220	226	278.5
Eremias argus	11.017	10.817	27.029	1000.776	28.650	-9.350	38.650	22.767	-2.275	22.925	-2.275	771	224	12	95.432	460	39	460	39.5
Eremias arguta	9.229	12.092	27.643	1027.825	27.300	-11.250	39.750	17.275	-0.117	20.983	-3.008	284.5	41	10.5	40.863	112	38	56	63
Eremias brenchleyi	9.754	12.325	28.965	1118.338	29.700	-12.900	42.600	21.950	-4.700	22.875	-4.700	466	143.5	2	116.990	320.5	9.5	316	9.5
Eremias grammica	16.767	15.029	35.148	1080.451	39.100	-6.500	45.250	8.850	29.633	30.133	3.583	200	37.5	0	83.614	99	1	2	69.5
Eremias intermedia	16.113	13.917	34.759	1002.946	35.800	-5.400	41.100	8.283	27.417	28.883	3.900	204	49	1	80.238	115	3	4	93
Eremias lalezharica	9.306	12.829	37.977	742.161	26.000	-7.600	33.600	0.992	9.725	18.533	0.233	153	31.5	2	78.213	83	8	14	70.5
Eremias montana	10.279	14.808	35.091	986.722	29.500	-12.000	42.200	1.283	21.967	22.283	-1.950	458	88	1	80.270	219	3	5	199
Eremias multiocellata	1.021	11.950	24.582	1395.077	24.200	-24.500	49.900	17.350	-14.800	17.350	-15.950	114	28	1	107.104	69	3	69	3
Eremias papenfussi	12.504	11.800	31.505	937.039	30.200	-7.250	37.450	5.767	23.500	23.758	0.833	223	43.5	1	71.259	104.5	5.5	9.5	85.5
Eremias persica	15.419	15.138	37.203	899.205	34.200	-5.200	40.100	6.700	25.283	26.092	4.183	191.5	40	0	83.074	103.5	3	5	88
Eremias pleskei	12.438	12.150	30.128	1010.418	30.900	-9.900	40.500	12.783	24.000	24.567	-0.617	256	43	6	54.645	110	22	39	54
Eremias przewalskii	4.579	13.275	25.142	1405.878	28.800	-24.500	52.800	20.550	-13.217	21.617	-14.600	100	29	1	99.000	67	4	67	4
Eremias strauchi	12.221	11.979	30.694	957.706	30.250	-9.350	38.600	11.708	23.583	24.008	-0.542	308	55.5	9	47.463	138.5	32.5	47.5	63.5
Eremias stummeri	4.587	13.025	31.086	1032.904	24.600	-17.300	41.900	14.817	-8.833	16.750	-8.833	557	76	20	43.690	224	67	207	67
Eremias suphani	8.733	11.833	31.556	919.262	25.900	-11.600	37.500	7.583	19.633	19.700	-2.883	381	60	10	48.996	158	35	57	89
Eremias velox	13.213	14.058	33.175	984.713	32.350	-7.000	41.400	7.342	23.750	24.733	1.717	258	54	1.5	83.661	136.5	7.5	12	99

Eremias vermiculata	8.829	14.717	31.701	1155.833	31.000	-16.900	47.900	22.083	-2.950	22.733	-6.567	66	17	1	79.174	39	3	38	4
Gallotia atlantica	20.079	7.917	52.896	238.650	27.500	13.000	14.800	17.533	21.967	23.317	17.250	133	31	0	85.860	74	2	5	74
Gallotia bravoana	NA	NA	NA	NA	NA	NA	NA	NA	NA	NA	NA	NA	NA	NA	NA	NA	NA	NA	NA
Gallotia caesaris	18.700	6.667	49.691	255.196	25.600	12.200	13.700	16.500	21.083	21.933	15.933	284	58	0	83.958	150	2	11	123
Gallotia galloti	18.215	6.787	51.075	252.038	25.100	11.900	13.900	16.650	20.858	21.558	15.367	353.5	67.5	0	80.815	181.5	3	15	146
Gallotia goliath	NA	NA	NA	NA	NA	NA	NA	NA	NA	NA	NA	NA	NA	NA	NA	NA	NA	NA	NA
Gallotia intermedia	20.527	6.612	51.251	235.547	27.200	14.300	12.900	18.033	22.783	23.550	17.850	233.5	49	0	85.293	125.5	2	7.5	103
Gallotia simonyi	16.004	6.842	45.611	302.155	23.500	8.500	15.000	12.933	19.350	20.067	12.717	344	65	1	77.446	172	5	18	146
Gallotia stehlini	19.727	7.454	50.762	254.497	27.500	12.950	14.550	17.025	22.608	23.358	16.933	221	40.5	1	74.896	108.5	4	12	94
Gastropholis prasina	22.856	7.654	63.024	159.059	28.700	16.650	12.050	23.433	22.417	24.750	20.842	1259	280	30.5	74.759	613	138	225	250
Gastropholis vittata	24.854	10.775	70.235	160.207	32.150	16.800	15.350	25.558	22.658	26.542	22.658	953.5	186	8	86.075	498	26	361.5	26
Heliobolus lugubris	21.079	16.708	59.354	370.025	34.000	6.600	27.300	24.867	16.700	25.317	16.700	371	86	0	105.400	234	2	153	3
Heliobolus spekii	24.058	10.817	73.019	128.808	31.200	16.800	14.400	24.500	23.117	25.467	22.767	637	140	4	83.011	327	18	183	32
Hellenolacerta graeca	13.500	10.258	36.203	677.594	27.100	-1.500	28.300	6.850	22.283	22.283	5.467	759	123	18	58.650	341	59	59	322
Holaspis guentheri	25.504	8.917	75.565	90.550	30.800	19.400	11.100	25.267	26.367	26.533	24.683	1803	296	32	60.169	647	152	431	442
Holaspis laevis	22.842	10.496	67.886	174.795	30.050	14.100	15.100	23.967	20.567	24.792	20.208	1485	266	39	63.268	656.5	121	411.5	121
Iberolacerta aranica	6.100	6.133	30.976	511.779	16.800	-3.000	19.800	5.950	13.200	13.200	1.383	1335	148	78	16.775	383	271	271	308
Iberolacerta aurelioi	3.021	5.058	26.623	497.409	13.100	-5.900	19.000	1.183	8.650	10.033	-1.733	1461	159	74	21.824	450	263	263	344
Iberolacerta bonnali	4.142	5.117	27.124	498.280	14.650	-4.400	18.800	1.892	11.383	11.383	-0.550	1359	146.5	73	19.976	402	252.5	252.5	325
Iberolacerta cyreni	10.125	10.017	36.318	637.027	24.200	-3.900	27.300	6.483	18.400	18.417	3.033	528	65	19	36.706	177	72	72	144
Iberolacerta galani	6.396	7.125	33.053	535.531	17.600	-3.800	21.900	1.650	13.767	13.767	0.933	1355	176	40	40.692	502	156	156	482
Iberolacerta horvathi	6.542	8.567	32.595	672.025	19.100	-6.700	26.800	6.500	0.117	14.750	-1.183	1599	180	89	20.791	513.5	295.5	406.5	330
Iberolacerta martinezricai	8.087	7.208	31.615	581.277	19.800	-3.000	22.800	2.900	15.983	15.983	1.900	1407	211	20	53.924	570	99	99	560
Iberolacerta monticola	10.750	7.342	38.369	475.438	20.600	0.000	19.700	6.850	16.450	16.550	5.583	1311	164	39	38.491	468	143	166	443
Ichnotropis capensis	20.129	15.675	57.166	382.934	33.100	5.500	27.700	23.467	14.567	24.017	14.567	603	121	2	87.336	328	10	285	10
Iranolacerta brandtii	8.775	12.233	33.905	899.395	26.000	-11.400	36.600	6.267	19.417	19.767	-2.350	308	54	5	59.511	138	17	30	80
Iranolacerta zagrosica	8.123	12.554	33.472	911.232	25.450	-12.200	37.500	-1.817	18.775	19.108	-3.058	277.5	55.5	1	79.641	139	6.5	9	118
Lacerta agilis	10.071	7.892	32.792	604.813	20.500	-3.200	23.700	7.733	6.033	16.417	2.467	786	88	42	22.125	254	143	178	209
Lacerta bilineata	11.458	9.117	33.898	626.739	23.800	-3.100	25.800	12.633	9.983	18.967	3.000	804	99	40	24.703	260	145	185	184
Lacerta media	11.473	11.363	34.507	768.227	27.450	-6.200	32.600	6.300	21.067	21.450	1.658	594	94	6	63.603	250.5	28	35	239.5
Lacerta pamphylica	18.071	10.458	36.458	647.402	31.000	3.500	28.100	10.233	25.233	25.417	10.233	744	146	5	85.959	392	25	28	392
Lacerta schreiberi	11.492	8.575	37.783	526.189	22.500	-0.100	22.600	6.667	18.233	18.433	5.483	1114	146	19	46.801	407	97	98	391
Lacerta strigata	13.706	9.729	31.308	805.696	28.400	-1.200	32.200	13.008	20.850	23.317	5.108	495.5	79.5	18	48.781	210	62.5	102.5	124
Lacerta trilineata	14.029	10.267	35.493	695.186	28.000	-1.000	28.800	8.133	22.517	22.733	5.817	615	105	13	51.296	280	50	62	244
Lacerta viridis	10.800	9.142	31.316	734.892	25.150	-5.700	29.850	10.700	16.508	20.500	1.300	589.5	75	29	27.992	204.5	97	138.5	137.5
Latastia longicaudata	23.521	10.975	73.135	118.561	30.800	16.500	14.500	23.967	22.917	24.817	22.567	600	127	3	81.513	271	16	176	33
Meroles anchietae	20.421	17.317	74.706	211.873	31.400	9.200	23.400	22.900	18.367	22.967	17.683	26	9	0	89.149	19	0	17	0
Meroles ctenodactylus	17.438	12.417	63.547	255.416	27.550	7.750	19.950	15.567	19.417	20.608	14.367	67	10	2	42.333	25	8.5	10	22
Meroles cuneirostris	21.429	17.533	74.716	212.544	32.800	9.300	23.500	23.617	18.833	24.083	18.833	26	9	0	89.149	19	0	17	0
Meroles knoxii	17.160	12.846	62.068	309.738	27.400	7.350	20.700	14.925	19.725	21.033	13.992	141	22	3	52.685	62	10	14.5	61
Meroles micropholidotus	19.475	12.833	72.097	184.629	27.600	9.700	17.800	21.567	17.175	21.567	17.150	9	4	0	69.282	6	0	6	0
Meroles reticulatus	19.367	12.058	68.920	195.922	28.000	10.200	17.500	21.733	17.200	21.733	16.883	26	10	0	97.039	19	0	19	0
Meroles squamulosus	21.783	14.546	56.197	359.619	33.450	7.200	26.200	25.033	16.267	25.300	16.267	583.5	112	4	83.584	312	15	275	15
Meroles suborbitalis	20.213	16.729	66.020	288.595	32.400	8.000	23.500	23.317	17.942	23.817	15.142	55	10	1	87.767	24	5	21	7
Mesalina adramitana	27.696	14.521	49.093	550.230	41.750	12.350	30.350	25.442	31.708	33.758	20.758	103.5	19.5	1	63.791	49	7	13	36
Mesalina bahaeldini	18.938	13.117	44.671	601.233	31.800	2.300	29.050	11.217	25.517	25.542	10.758	150	36.5	0	102.987	85.5	0	0	85.5
Mesalina balfouri	25.783	6.367	60.949	148.770	31.500	20.700	10.800	24.450	26.750	27.600	24.250	93	23	1	67.546	47	9	14	27
Mesalina bernoullii	19.200	14.242	38.993	840.477	35.900	-0.750	37.050	9.483	28.800	28.800	8.642	124	24	0	88.674	61	0	0	56
Mesalina brevirostris	26.337	13.342	39.265	699.062	40.400	7.700	33.250	18.417	33.442	34.025	17.450	106.5	24.5	0	90.318	60.5	0.5	1	51.5
Mesalina guttulata	19.727	13.258	41.442	696.002	34.100	3.300	32.300	12.783	27.433	27.692	11.283	131	25	0	75.859	66	2	2.5	42.5
Mesalina kuri	25.660	6.679	65.479	120.075	31.050	20.850	10.200	24.800	26.658	27.417	24.508	49.5	20	0	110.539	34	1	5.5	15
Mesalina martini	23.858	9.583	60.273	248.183	31.600	15.700	15.900	20.767	25.450	26.500	20.767	242	48	1	72.573	123	20	25	123

Mesalina microlepis	16.796	12.883	38.249	744.528	31.600	-1.400	32.500	7.550	25.067	25.067	7.550	230	45	0	83.373	115	1	2	115
Mesalina olivieri	19.912	12.500	40.343	676.808	33.600	3.400	31.500	14.150	27.083	27.350	11.967	146	23	0	60.570	60	4	11	49
Mesalina pasteuri	26.744	13.233	49.542	476.748	40.200	13.200	27.050	31.233	25.683	33.217	20.800	40.5	16.5	0	116.934	33	2	26	4.5
Mesalina rubropunctata	24.317	14.412	46.455	602.137	38.900	7.700	29.150	23.133	26.342	31.533	15.267	24.5	4	0	64.729	10	0	0	4
Mesalina saudiarabica	27.321	15.825	46.921	620.465	42.400	8.700	34.000	27.700	34.317	34.317	18.517	109	23	1	69.282	50	6	7	26
Mesalina simoni	19.133	13.700	45.667	578.389	33.900	3.900	30.000	13.567	26.350	26.583	12.517	251	39	1	62.967	114	8	10	114
Mesalina watsonana	16.996	14.883	37.069	941.612	35.600	-1.500	40.600	7.183	27.467	28.367	5.683	232	38	1	95.128	100	3	3	84
Nucras boulengeri	23.079	11.908	74.408	127.181	30.400	15.000	15.900	23.033	21.950	24.517	21.783	903	181	0	94.465	430	3	260	30
Nucras intertexta	19.604	14.983	55.957	352.320	32.750	6.600	25.850	23.575	14.383	23.983	14.383	568.5	104.5	5	81.475	289	18	283	18
Nucras lalandii	14.787	13.492	54.577	352.426	26.900	2.600	24.600	18.583	10.200	18.817	10.150	838	144	10	73.848	394	41	386	43
Nucras livida	16.942	14.208	53.697	438.874	29.250	2.950	26.650	19.558	12.200	21.633	11.467	296.5	39.5	13	26.484	103.5	47	67	51.5
Nucras taeniolata	17.454	13.575	60.874	303.146	28.000	6.400	22.000	18.017	14.167	21.217	13.717	620	72	26	35.005	207	83	191	93
Nucras tessellata	18.058	14.433	55.012	418.685	30.000	4.000	25.300	20.117	18.133	22.700	12.550	188	30	3	52.282	84	13	36	33
Omanosaura cyanura	25.852	10.483	40.807	568.129	37.900	12.000	25.400	19.725	26.958	32.217	18.433	223.5	50	3	80.255	120	16	45	94
Omanosaura jayakari	25.608	11.483	43.301	565.398	37.600	10.700	25.700	21.083	26.350	31.867	18.167	216	48	3	76.951	115	16	44	77
Ophisops beddomei	26.796	10.900	59.549	273.411	33.800	14.700	19.100	26.083	24.650	29.033	22.567	2809	1070	1	151.507	2290	3	235	7
Ophisops elegans	15.458	12.950	37.528	886.712	31.500	-4.000	38.300	8.067	24.600	24.817	5.133	386	75	1	85.423	199	4	5	178
Ophisops jerdonii	24.208	14.333	43.259	788.903	41.000	5.000	36.100	29.450	21.317	32.500	13.633	375	106	3	105.601	234	18	48	45
Ophisops leschenaultii	26.308	8.450	65.000	127.401	32.300	19.300	13.000	24.717	27.383	27.433	24.500	1784	368	10	73.867	912	73	220	308
Ophisops microlepis	NA	NA	NA	NA	NA	NA	NA	NA	NA	NA	NA	NA	NA	NA	NA	NA	NA	NA	NA
Ophisops occidentalis	17.085	9.788	38.198	589.303	29.600	4.000	25.600	13.242	24.258	24.350	10.058	279.5	49	4.5	69.753	128.5	23.5	24	120.5
Parvilacerta fraasii	8.756	11.271	38.447	672.846	22.300	-7.000	29.300	0.592	16.783	16.783	0.467	778	165.5	0	93.321	441	3	3	422.5
Parvilacerta parva	7.246	11.975	35.454	794.741	25.600	-9.100	34.000	9.383	18.817	18.817	-1.700	432	66	16	40.466	167	60	69	141
Pedioplanis burchelli	13.325	12.583	52.504	405.622	24.900	0.300	23.900	15.983	9.783	18.033	7.767	438	57	14	35.391	160	48	120	65
Pedioplanis gaerdesi	21.865	15.867	70.834	231.206	32.100	9.700	22.500	24.167	18.942	24.167	18.467	113.5	38	0	123.015	89	0	78.5	0
Pedioplanis inornata	21.000	16.675	67.645	269.134	32.700	8.700	23.300	23.583	17.967	24.050	17.000	112	30	0	95.977	77	2	66	2
Pedioplanis laticeps	17.517	15.800	52.389	501.935	31.400	1.400	29.800	20.333	13.367	23.850	10.800	203	29	8	47.542	80	26	50	31
Pedioplanis lineoocellata	17.985	15.558	55.094	437.879	30.500	3.500	28.050	21.092	13.575	22.633	12.483	206.5	43	2	67.769	117	7	86	12
Pedioplanis namaquensis	19.838	16.583	55.537	457.903	32.600	4.000	28.600	23.767	14.033	24.217	13.067	182	42	1	90.531	107	6	83	6
Pedioplanis undata	20.804	16.342	68.310	294.075	32.150	7.400	24.900	23.042	16.700	23.617	16.700	285	77	0	116.533	206	1	100	1
Philochortus spinalis	28.137	10.692	53.998	316.430	38.300	18.500	19.800	24.517	30.267	32.433	24.517	287	50	2	61.997	135	26	39	135
Phoenicolacerta cyanisparsa	14.171	12.392	34.499	921.753	31.900	-5.000	37.200	3.067	25.167	25.250	3.067	593	105	2	78.904	292	9	12	292
Phoenicolacerta kulzeri	11.513	11.267	38.173	674.097	26.250	-5.100	29.300	2.567	20.133	20.167	2.567	814	189	0	101.630	504	1	2	504
Phoenicolacerta laevis	16.771	9.958	36.811	653.300	29.900	3.000	27.700	9.017	24.317	24.617	9.017	804	174	1	91.420	459	10	14	459
Phoenicolacerta troodica	18.288	10.000	37.713	616.439	31.300	5.300	26.500	11.133	25.567	25.767	11.133	470	110	1	92.062	275	10	10	244
Podarcis bocagei	13.077	8.079	41.692	462.566	22.350	1.600	20.850	8.325	17.992	18.417	7.650	1295	176	30.5	48.195	510	121	146	510
Podarcis carbonelli	14.896	8.575	41.867	488.179	24.000	5.450	22.350	11.400	19.375	19.517	10.592	857.5	125	11	57.128	353.5	58	62.5	335.5
Podarcis cretensis	17.863	7.367	31.890	551.422	27.800	6.100	22.100	11.067	24.517	24.517	11.067	576	115	1	84.486	301	6	6	301
Podarcis erhardii	17.142	6.583	30.948	567.332	27.400	6.300	21.800	11.233	24.133	24.133	10.650	492	81	2	81.783	229	10	10	208
Podarcis filfolensis	18.792	6.725	33.292	502.084	28.600	8.700	20.200	15.100	24.633	25.183	13.150	516	99	0	83.247	262	7	44	177
Podarcis gaigeae	16.865	5.750	26.867	609.472	27.350	6.250	21.650	10.575	24.350	24.350	10.167	406.5	68	7	66.505	190.5	24.5	24.5	170.5
Podarcis guadarramae	11.858	9.350	39.222	559.305	23.800	-0.900	24.000	7.317	18.967	19.067	5.600	1006	143	19	47.046	402	91	93	380
Podarcis hispanicus	13.212	11.742	39.325	688.600	29.000	-3.200	30.500	9.267	22.383	22.617	5.167	453	60	9	42.684	159	47	56	140
Podarcis lilfordi	17.133	8.783	39.211	520.258	28.000	5.800	22.400	15.333	23.533	23.817	11.533	502	79	4	51.138	213	39	76	140
Podarcis liolepis	14.023	9.979	38.365	580.554	27.300	0.850	25.700	15.675	17.875	21.808	7.000	680	84	29	26.293	220	126.5	159	138
Podarcis melisellensis	14.308	7.146	29.595	654.195	26.500	2.100	24.800	13.350	22.592	22.825	6.375	886.5	114	36	30.612	315	143.5	143.5	240
Podarcis milensis	18.158	6.367	30.609	553.706	28.200	7.400	20.800	13.350	25.083	25.083	11.783	419	75	1	82.979	215	7	7	183
Podarcis muralis	9.771	8.250	32.288	623.441	21.800	-3.700	25.000	10.917	5.350	17.650	2.133	788	85	47	16.388	233	157	194	195
Podarcis peloponnesiacus	14.779	10.517	39.095	642.024	28.800	1.200	26.900	8.600	23.250	23.250	7.167	746	134	11	69.411	374	39	39	330
Podarcis pityusensis	17.738	8.992	38.924	525.199	29.900	6.400	23.300	15.717	24.317	24.700	12.050	414	65	5	50.725	169	35	66	130
Podarcis raffonei	17.692	6.467	31.856	513.552	28.300	8.000	20.300	16.267	23.933	24.383	11.950	618	89	8	57.284	254	33	78	203
Podarcis siculus	15.431	8.533	34.022	612.217	28.000	2.800	25.100	13.700	23.125	23.325	8.517	689.5	93	18.5	41.200	251	79.5	88.5	191.5

Podarcis tauricus	12.419	8.779	30.770	751.425	26.550	-2.800	28.600	12.050	20.967	21.850	3.592	529	65.5	25	26.153	187	84	110.5	136.5
Podarcis tiliguerta	14.988	8.138	34.088	594.492	27.000	2.950	24.050	12.258	22.400	22.525	8.308	752.5	107	11	48.518	293.5	59.5	75.5	229.5
Podarcis vaucheri	13.727	10.513	38.947	661.686	27.350	-1.650	29.000	8.742	21.917	22.250	5.908	532.5	78	4	53.531	215	29	30	195.5
Podarcis virescens	14.567	11.637	38.882	668.390	28.600	-1.000	29.900	9.725	22.517	22.750	6.767	509	66.5	8	46.367	182.5	42	47	162.5
Podarcis waglerianus	17.250	8.379	35.157	546.793	28.850	5.800	23.550	15.458	23.842	24.233	11.192	495.5	75.5	3.5	60.974	216.5	22	49	179.5
Poromera fordii	23.754	8.000	71.413	104.747	28.700	17.150	11.250	23.408	23.642	24.642	22.308	1947	340.5	8	68.236	839	52.5	584	186
Psammodromus algirus	14.279	10.258	38.474	590.427	27.400	0.900	26.300	13.450	21.650	22.017	7.400	610	82	18	32.261	215	97	115	141
Psammodromus blanci	14.200	12.358	38.617	721.743	29.750	-2.250	32.000	12.383	23.583	23.583	5.808	369.5	42.5	11.5	28.947	116	52.5	52.5	98.5
Psammodromus edwarsianus	15.329	11.254	39.702	639.172	29.400	0.400	29.050	12.042	23.308	23.558	7.925	445.5	64	6	48.447	162	34	43	136
Psammodromus hispanicus	13.571	12.092	38.218	711.641	29.100	-2.300	31.800	9.583	21.883	22.067	5.817	441	58	9	42.390	161	47	47	134
Psammodromus microdactylus	16.158	11.317	41.002	633.815	30.700	0.900	29.800	9.750	24.150	24.367	8.767	440	61	2	57.826	164	20	20	159
Psammodromus occidentalis	13.800	10.667	40.906	528.446	28.900	0.400	25.000	9.183	22.967	23.083	7.567	539	79	13	52.659	225	54	58	203
Pseuderemias smithii	28.844	11.013	80.867	76.411	35.850	22.200	13.650	29.342	28.458	29.783	28.100	245.5	75.5	1	109.951	131.5	6	63	47.5
Scelarcis perspicillata	13.369	10.667	38.145	660.241	27.700	-1.600	28.450	6.892	21.492	21.667	5.958	585	78	4	58.025	213.5	27	27	194
Takydromus amurensis	8.548	10.871	25.251	994.126	27.150	-10.500	38.800	19.933	-4.183	20.500	-4.183	1256	282.5	27	83.159	691.5	91.5	686.5	91.5
Takydromus dorsalis	22.658	3.817	26.878	386.904	29.300	15.100	14.200	25.967	19.433	27.017	17.750	2366	255	153	18.654	740	476	637	484
Takydromus formosanus	22.246	7.333	40.221	412.497	30.200	12.400	18.300	26.267	18.567	26.583	16.883	2432	467	25	85.045	1279	104	1279	155
Takydromus hsuehshanensis	9.129	9.358	44.777	452.310	19.500	-1.400	20.900	13.200	4.633	13.933	3.150	3494	478	78	52.609	1391	291	1128	410
Takydromus intermedius	17.725	6.550	25.586	699.401	29.500	3.900	25.600	25.983	8.633	25.983	8.633	1152	219	18	79.165	610	59	610	59
Takydromus luyeanus	22.854	6.608	38.960	348.741	31.600	14.000	16.200	26.467	18.083	27.700	18.083	2145	368	48	72.811	1035	153	984	153
Takydromus sauteri	23.844	6.125	40.735	328.607	30.000	16.400	15.050	26.383	20.533	26.725	19.808	2680	478	23	92.769	1271	92	1271	116.5
Takydromus septentrionalis	16.750	7.725	24.833	859.460	31.700	1.000	31.450	24.650	7.867	26.992	5.950	1397	215.5	37	51.208	536.5	136.5	513	165
Takydromus sexlineatus	24.375	8.167	57.564	178.664	31.600	16.350	13.550	26.058	22.592	27.108	21.967	1943.5	349.5	15.5	78.866	949	69	520	87.5
Takydromus smaragdinus	22.719	4.754	27.874	456.740	30.800	14.100	16.950	23.925	17.125	28.100	17.125	2132.5	246.5	110	26.488	668.5	362	610	368
Takydromus stejnegeri	22.160	6.771	36.693	458.943	31.750	12.400	19.300	26.742	19.233	27.917	16.092	2319	382.5	20.5	74.357	977	93.5	859	178.5
Takydromus sylvaticus	16.096	7.825	25.161	849.589	32.000	0.900	31.100	19.983	7.400	26.267	5.367	1696	295	43	56.629	766	163	575	211
Takydromus tachydromoides	15.192	7.933	26.907	805.274	30.900	0.700	30.600	22.300	5.783	25.067	5.383	1626	235	50	48.678	603	182	567	188
Takydromus toyamai	23.596	4.500	29.003	405.181	31.000	15.500	15.500	27.108	18.625	28.275	18.525	2177.5	240	134.5	18.595	666.5	439	611	440.5
Takydromus viridipunctatus	21.696	5.208	29.593	449.821	30.000	13.000	17.600	25.667	16.750	27.283	16.150	2831	387	149	34.040	1051	472	921	472
Takydromus wolteri	11.910	9.662	26.693	967.143	30.100	-6.200	36.400	23.250	-0.192	23.517	-0.242	1247.5	270	29	78.569	669.5	96.5	659	96.5
Teira dugesii	17.479	5.492	43.061	269.536	24.100	11.100	12.700	15.783	20.183	21.058	14.350	709	110	6	61.209	304.5	31	70.5	246.5
Timon kurdistanicus	13.167	12.517	31.000	1007.103	33.400	-8.100	40.300	4.083	25.267	25.533	0.800	761	123	1	79.084	360	5	6	338
Timon lepidus	14.313	11.783	39.478	648.372	28.300	-0.500	30.200	9.550	22.733	22.817	7.200	574	79	6	49.688	225	36	41	210
Timon nevadensis	12.742	12.517	38.701	722.158	28.950	-3.600	32.550	5.792	22.017	22.450	4.850	459	58	6	47.680	167	34	37	159.5
Timon pater	16.804	10.725	38.009	635.831	30.000	2.600	28.300	10.333	24.217	24.600	9.267	687	104	7	63.498	299	41	52	265
Timon princeps	13.981	15.017	38.752	881.927	32.550	-6.900	39.350	4.425	24.675	24.675	3.258	245.5	50.5	0	87.203	132.5	5	6	127
Timon tangitanus	11.542	10.675	37.128	665.823	26.100	-3.200	29.300	8.650	20.217	20.250	3.717	421	53	5	49.144	149	31	32	135
Tropidosaura cottrelli	6.779	11.358	48.540	401.047	18.100	-5.300	23.400	10.650	1.783	11.433	1.517	896	135	11	65.791	381	41	381	48
Tropidosaura essexi	11.873	11.625	50.684	373.898	22.300	0.500	23.450	15.625	7.342	15.625	7.300	895.5	153.5	9.5	72.164	427	41.5	427	46
Tropidosaura gularis	15.352	10.567	52.692	352.594	25.000	5.450	20.450	15.708	13.300	18.958	11.242	494	56	26	18.845	153	88	119	102
Tropidosaura montana	13.804	11.108	52.424	325.656	24.800	3.700	20.500	16.867	14.983	18.250	9.567	646	98	21	49.119	259	76	233	109
Vhembelacerta rupicola	17.354	11.992	54.205	352.068	27.400	5.200	22.200	20.817	12.467	20.817	12.467	832	169	9	83.124	444	32	444	32
Zootoca vivipara	8.721	7.325	36.716	467.325	19.000	-1.100	20.100	6.600	8.267	15.283	3.567	802	91	46	23.769	264	153	198	224

Supplementary Table 15. Lacertid species included in the phylogenetic analysis, and associated bioclimatic variables related to solar radiation, given as the median of all localities per species (see Supplementary Methods for an explanation of variables).

Species	bio20	bio27	bio25	bio26	bio24	bio23	bioSR1	bioSR2	bioSR3	bioSR4
Acanthodactylus aegyptius	235555	36499	26809.334	74377.000	12027.667	31.610	76	2	13.950	26.917
Acanthodactylus arabicus	250630	56076	20402.000	61458.000	20593.334	8.792	14	18	25.917	36.150
Acanthodactylus aureus	246042	50317	24075.334	61721.000	21498.334	15.971	7	2	18.883	23.750
Acanthodactylus bedriagai	213635.5	31322	25268.833	75806.500	17735.334	36.548	95.5	47	7.000	24.042
Acanthodactylus beershebensis	233475	34867	26915.334	74748.000	11622.333	33.399	121	3	11.517	25.367
Acanthodactylus blanci	214649	31926	25677.334	77032.000	20093.000	35.691	106	52	7.667	24.750
Acanthodactylus blanfordii	236344	43205.5	23499.833	71976.500	15698.667	21.040	56	9	17.267	33.342
Acanthodactylus boskianus	237101	38221	26201.334	77071.000	12957.333	30.207	41	1	12.717	27.650
Acanthodactylus boueti	204749.5	40771	17639.333	57926.000	15885.334	13.501	533.5	183.5	24.058	28.125
Acanthodactylus busacki	222079	40999	23077.334	64293.000	13499.667	20.881	43	5	17.233	23.817
Acanthodactylus cantoris	234351	46139	17246.000	70365.000	19567.666	18.206	28	21	19.567	32.050
Acanthodactylus dumerilii	243818	52036	23487.666	60608.000	20800.666	13.150	4	2	21.200	28.067
Acanthodactylus erythrurus	204478	28371	24761.334	70812.000	9114.333	40.795	141	34	9.950	23.617
Acanthodactylus felicis	252221	56271	21083.334	71536.000	20263.000	11.004	28	36	18.750	26.750
Acanthodactylus gongrorhynchatus	253608	50495	24333.000	72236.000	20951.666	16.519	15	10	19.633	35.167
Acanthodactylus grandis	222956	34496	25090.666	75272.000	11590.667	32.582	160	0	12.700	35.283
Acanthodactylus guineensis	238726.5	56762.5	19296.167	63592.500	18509.666	7.315	289.5	105.5	25.750	31.458
Acanthodactylus haasi	250616	50953	20915.334	70237.000	21038.666	14.011	24	24	20.650	34.817
Acanthodactylus hardyi	233702	38623	25780.334	77341.000	12481.833	28.325	43	0	12.083	32.008
Acanthodactylus harranensis	215942	27315	25184.666	82410.000	9105.000	42.312	152	7	7.083	28.350
Acanthodactylus khamirensis	243151.5	45303.5	24513.333	72355.000	15101.167	20.373	88	1.5	19.075	33.642
Acanthodactylus lineomaculatus	220344	36961	24035.000	67383.000	11531.000	28.589	150	13	14.183	22.233
Acanthodactylus longipes	244636	42250	26022.666	74611.000	14083.333	25.672	12	1	14.050	28.250
Acanthodactylus maculatus	222133.5	33893.5	25875.333	77539.000	15939.833	33.968	70.5	16.5	10.675	26.133
Acanthodactylus margaritae	223126.5	39738.5	23020.833	65035.000	13246.167	24.332	96.5	6	15.200	23.450
Acanthodactylus masirae	251479	52518	21175.000	72491.000	19300.334	13.104	17	26	22.867	29.833
Acanthodactylus micropholis	236987.5	43695.5	21278.834	72025.500	16074.667	21.697	56.5	12	15.050	32.167
Acanthodactylus nilsoni	217103	31156	26746.000	80238.000	12430.000	37.719	192	0	10.333	33.800
Acanthodactylus opheodurus	253746	51811.5	22877.667	73308.000	21559.167	16.418	26	14.5	19.717	33.575
Acanthodactylus orientalis	NA	NA	NA	NA	NA	NA	NA	NA	NA	NA
Acanthodactylus pardalis	234065	35687	26481.666	74550.000	11800.000	32.555	87	0	13.950	25.583
Acanthodactylus robustus	226146	33709	26903.666	80711.000	11236.333	35.086	58	0	10.867	34.300
Acanthodactylus savignyi	219322	34114	25599.334	71324.000	10697.667	33.415	184	1	13.583	26.267
Acanthodactylus schmidti	258536	51491	23830.666	73971.000	20992.000	16.606	21	11	19.750	35.133
Acanthodactylus schreiberi	227197.5	34305	26753.000	73874.000	10843.500	34.477	338	3	13.150	25.950
Acanthodactylus scutellatus	236511	37842	26073.000	78219.000	14138.333	29.904	34	2	12.617	28.217
Acanthodactylus senegalensis	242658	52954	23742.000	59373.000	20719.000	12.957	5	3	22.350	28.150
Acanthodactylus taghitensis	245653	41702	26883.334	79766.000	14492.000	27.836	19	4	11.867	33.550
Acanthodactylus tilburyi	230960	38431	25020.666	75798.000	18564.334	27.859	7	0	11.267	29.850
Acanthodactylus tristrami	231445.5	34954.5	27118.500	75777.000	11651.500	33.682	162	0	6.550	22.283
Adolfus africanus	184572	46311.5	16015.834	45977.000	15053.667	5.058	430	272	19.225	20.083
Adolfus alleni	193257.5	42865.5	18023.667	53517.500	16090.667	11.451	299.5	246	5.208	6.800
Adolfus jacksoni	192266	44575	15576.834	49433.000	15692.500	5.338	401	237.5	17.275	18.658
Algyroides fitzingeri	184795.5	21942.5	24237.000	66046.500	8235.333	47.636	252.5	57.5	7.900	22.533
Algyroides marchi	201134.5	27086	23199.000	77351.500	9477.000	42.454	166	46	4.208	21.908
Algyroides moreoticus	185706	22356	24005.334	72016.000	6967.333	45.098	330	48	6.750	21.600

Algyroides nigropunctatus	179067	20464	23761.000	68626.000	8355.333	48.640	403	60	8.433	22.900
Anatololacerta anatolica	205876	24667	26811.334	80537.000	8222.333	45.129	356	13	8.900	23.700
Anatololacerta danfordi	210774.5	27609.5	24672.500	79423.500	9286.167	41.031	280	34	4.083	21.658
Anatololacerta oertzeni	213859	27341	26809.000	79947.000	9090.333	41.738	397	9	12.067	25.800
Apathya cappadocica	209805.5	25892.5	25408.667	79319.000	8824.667	44.227	257.5	19.5	1.950	24.125
Apathya yassujica	228222.5	36904	24116.166	76298.000	14445.667	29.133	110	6.5	1.783	23.817
Archaeolacerta bedriagae	183393.5	22460.5	23975.334	71243.500	8239.000	46.467	322.5	78	3.758	18.158
Atlantolacerta andreanskyi	227108.5	38380	24411.666	73235.000	18961.167	27.596	189	67.5	0.275	16.525
Australolacerta australis	231596	34165.5	27654.000	75516.000	11388.500	36.338	173	53	9.042	18.783
Congolacerta asukului	162112	38397	13559.333	40457.000	13752.333	5.786	507	366	11.767	12.733
Congolacerta vauereselli	171045	43395	14708.667	42719.000	13925.333	5.260	494	232	15.150	15.767
Dalmatolacerta oxycephala	172905.5	19215	23652.500	70957.500	7349.167	51.170	293.5	117	9.342	24.125
Darevskia caspica	179687	28225	21181.666	63609.000	10226.667	34.419	196	58	7.183	24.233
Darevskia caucasica	165537	21130	7043.333	57951.000	21170.666	44.433	105	288	-6.400	13.550
Darevskia chlorogaster	180037.5	27668	21481.333	64550.000	10844.500	35.010	144	52	5.425	24.000
Darevskia clarkorum	164308	22106	16313.333	52917.000	8443.667	39.560	550	329	5.267	19.517
Darevskia daghestanica	160481	19288	6429.333	63145.000	21071.000	47.218	63	184	-0.867	21.083
Darevskia defilippii	200073	28884	23070.000	73577.000	14434.333	37.074	97	22	-1.150	19.633
Darevskia derjugini	163706.5	21703	15038.667	60604.500	13528.334	44.924	327.5	274.5	-1.717	17.825
Darevskia kamii	181723	27690	21545.334	64636.000	13415.667	34.722	110	31	6.367	24.583
Darevskia kopetdaghica	201764	27335	23563.334	76904.000	14178.333	41.066	108	47	-5.617	17.467
Darevskia lindholmi	158928	22903	15100.667	66889.000	4867.000	53.815	141	106	3.500	21.433
Darevskia mixta	164355	21330	16368.000	59853.000	6522.000	41.395	521	347	3.450	19.200
Darevskia parvula	170149	22536	16791.000	61489.000	8359.667	40.254	434	288	3.567	18.583
Darevskia portschinskii	175015	22310.5	7436.833	64599.500	20122.333	43.987	70	187.5	1.192	19.975
Darevskia praticola	158508	17274	8313.000	64140.000	19596.000	49.692	143	222	0.683	21.483
Darevskia raddei	181651.5	23894.5	8074.000	67275.000	19706.667	42.723	77.5	128	-1.950	18.417
Darevskia rudis	182853	23854	10494.667	63522.000	20000.666	42.096	207	212	-0.733	15.117
Darevskia saxicola	163049	20400	9122.333	62474.000	20786.000	44.626	203	280	-3.083	14.983
Darevskia schaekeli	195516	29299	21781.666	70126.000	14863.667	35.199	58	21	-0.650	20.467
Darevskia steineri	190941.5	27763.5	23300.333	69901.000	13818.667	37.298	96	17	5.442	24.500
Darevskia valentini	184616	23606	7998.000	66109.000	20550.000	42.880	103	183	-8.383	14.083
Dinarolacerta montenegrina	167482.5	20196.5	22110.833	66332.500	7607.500	46.424	335.5	209.5	-2.258	13.742
Dinarolacerta mosorensis	161897.5	22904	15513.500	63937.500	7289.167	45.888	319	226	-2.867	12.467
Eremias argus	174885.5	28340	9447.000	58236.000	17707.167	29.841	53	287	-2.275	22.925
Eremias arguta	182672	23135	10639.834	66975.500	20867.667	43.680	71.5	51.5	-3.008	20.983
Eremias brenchleyi	188463.5	29560.5	9853.500	62412.000	19732.667	32.634	15	233	-4.700	22.875
Eremias grammica	205131	25876	24396.667	78614.500	12804.667	44.226	66	2	3.583	30.133
Eremias intermedia	202340	24795	24233.000	78691.000	12807.333	42.806	74	4	3.900	29.000
Eremias lalezharica	226864	39604	17950.333	71203.500	15161.834	23.884	48	21	0.233	18.533
Eremias montana	208025	28605	23880.666	77532.000	11555.667	39.274	187	5	-1.950	22.283
Eremias multiocellata	181351	23011	7782.333	64028.000	21342.666	43.122	5	54	-15.950	17.350
Eremias papenfussi	204244	29182	23116.833	75513.000	14597.667	37.733	73	9.5	0.833	23.758
Eremias persica	218343.5	33619	23993.667	76297.000	13695.167	32.980	70.5	5	4.183	26.117
Eremias pleskei	192243	24902	22867.666	73784.000	17343.666	42.653	59	39	-0.617	24.567
Eremias przewalskii	191371	26410	8894.000	65655.000	21552.000	38.557	6	49	-14.600	21.617
Eremias strauchi	190659.5	24455	22605.666	73187.500	17193.834	42.496	67	54	-0.542	24.008
Eremias stummeri	182160	23883	7961.000	67774.000	22738.666	41.988	83	224	-8.833	16.750
Eremias suphani	194246	25315	23168.666	74488.000	17462.666	42.045	91	57	-2.883	19.700
Eremias velox	200706.5	26064.5	23105.834	74538.000	14301.834	40.988	81	12	1.717	24.733
Eremias vermiculata	201380	29214	9714.667	69476.000	23082.666	34.948	4	36	-6.567	22.733
Gallotia atlantica	228207	39352	24676.000	69054.000	12917.667	26.953	73	3	17.250	23.317

Gallotia bravoana	NA	NA	NA	NA	NA	NA	NA	NA	NA	NA
Gallotia caesaris	235130	47857	25910.000	72266.000	12570.000	28.664	149	4	15.933	21.933
Gallotia galloti	229517.5	46468	25291.834	70838.500	12169.834	28.582	179.5	6	15.367	21.558
Gallotia goliath	NA	NA	NA	NA	NA	NA	NA	NA	NA	NA
Gallotia intermedia	232555.5	47599.5	25598.500	71686.500	13190.334	28.209	124	2	17.850	23.550
Gallotia simonyi	233197	48064	25318.000	71185.000	13346.000	27.316	171	13	12.717	20.067
Gallotia stehlini	236605.5	46942	25971.000	72590.500	13578.667	27.320	103.5	6	16.933	23.358
Gastropholis prasina	202711.5	44313	16616.667	56706.500	15760.334	13.018	459.5	161.5	20.842	24.750
Gastropholis vittata	199412	45011.5	15003.834	54037.000	15883.000	11.654	136.5	171	22.642	26.517
Heliobolus lugubris	248164	49876	16625.334	70718.000	22746.334	14.811	6	128	16.683	25.300
Heliobolus spekii	217164	51344	17920.334	57613.000	18134.334	8.049	101	83	22.767	25.467
Hellenolacerta graeca	182868	21841	23717.666	71153.000	6988.333	45.238	342	59	5.467	22.283
Holaspis guentheri	173370	36961	15569.333	48451.000	13402.667	13.456	442	431	24.683	26.533
Holaspis laevis	189546.5	39228.5	13626.833	54002.500	15750.500	16.434	225	288	20.533	24.792
Iberolacerta aranica	170461	29828	19634.666	58904.000	19367.334	44.253	356	288	1.383	13.200
Iberolacerta aurelioi	179379	42494	20813.334	62440.000	8311.000	43.544	435	277	-1.733	10.033
Iberolacerta bonnali	173561.5	35401.5	20403.000	61209.000	7707.500	45.061	395.5	270	-0.550	11.383
Iberolacerta cyreni	188650	22769	22773.334	68432.000	8285.333	47.298	166	80	3.033	18.333
Iberolacerta galani	174104	19782	21140.666	63293.000	5904.000	48.994	502	160	0.933	13.767
Iberolacerta horvathi	143173.5	15307	7408.500	58346.500	8812.833	52.232	416	402.5	-1.475	14.675
Iberolacerta martinezricai	192253	22924	23282.334	69847.000	6862.000	47.765	570	121	1.900	15.983
Iberolacerta monticola	160996	18215	21021.000	57279.000	5434.000	48.208	468	175	5.583	16.633
Ichnotropis capensis	242069	48715	16355.667	70506.000	23049.000	16.123	19	238	14.733	24.050
Iranolacerta brandtii	192294	25492	23117.000	74406.000	16669.334	42.460	81	30	-2.350	19.767
Iranolacerta zagrosica	217224.5	33237	23886.666	76578.500	14431.167	33.501	99.5	9	-3.058	19.108
Lacerta agilis	124604.5	10100	9803.333	52947.500	4320.000	61.190	233	176	2.467	16.417
Lacerta bilineata	166427	18766	16536.334	63688.000	11081.667	50.070	203	183	2.983	18.967
Lacerta media	207249	25609	24787.667	75417.500	10634.167	43.356	225	36	1.658	21.450
Lacerta pamphylica	216767	27841	25382.334	75835.000	9260.333	41.428	378	27	9.133	25.217
Lacerta schreiberi	185277	22006	23095.334	67587.000	6926.000	47.337	407	99	5.483	18.433
Lacerta strigata	173144.5	26406.5	20287.167	64351.000	11481.000	40.760	111	83	5.108	23.317
Lacerta trilineata	187893	22793	24106.500	74807.000	7563.000	47.291	268	60.5	5.842	22.742
Lacerta viridis	168409	18974	19746.833	66789.000	8704.167	49.866	165.5	142	1.333	20.592
Latastia longicaudata	219599	53625	18122.334	58128.000	18097.666	6.977	97	79	22.567	24.817
Meroles anchietae	236378	50873	17708.334	61465.000	22152.000	17.374	2	7	17.683	22.967
Meroles ctenodactylus	238796	40184.5	26345.333	74403.000	12792.000	29.028	23	9	14.367	20.608
Meroles cuneirostris	237807	53062	17694.833	61465.000	22192.000	17.436	2	7	18.833	24.083
Meroles knoxii	230884	38680.5	26376.000	73919.000	12231.000	31.334	60.5	13	13.983	21.033
Meroles micropholidotus	228355	49690	16742.834	65046.000	21668.500	18.460	1	1	17.150	21.567
Meroles reticulatus	217924.5	45960	15260.000	61437.500	20467.834	17.138	1	7	16.875	21.725
Meroles squamulosus	236335	47380	15901.667	67120.500	22225.166	17.344	22	256.5	16.267	25.325
Meroles suborbitalis	239637.5	45985	17736.000	71740.000	22261.000	23.694	8	9	15.142	23.817
Mesalina adramitana	250616.5	51402.5	21004.667	69456.000	21486.500	14.503	30	27	20.758	33.758
Mesalina bahaeldini	233702.5	36458.5	26868.167	77843.500	12152.833	32.085	77	0	10.758	25.542
Mesalina balfouri	264076	59191	22845.000	72025.000	19580.000	9.814	44	20	24.217	27.600
Mesalina bernoullii	228876.5	34377	26730.000	80145.500	11505.333	33.609	58	0	8.642	28.808
Mesalina brevirostris	233499	42765	24844.833	73509.000	15991.667	21.489	47	1	17.450	34.025
Mesalina guttulata	233898	38905.5	25844.000	77190.000	14595.667	28.952	48	4	11.283	27.692
Mesalina kuri	268204	59315	23862.666	72969.000	19568.334	8.787	35	6	24.517	27.450
Mesalina martini	253759	53946	24218.334	65076.000	17982.000	11.613	123	20	20.767	26.500
Mesalina microlepis	215725	28993	25806.000	77418.000	9742.667	38.552	107	2	7.550	25.067
Mesalina olivieri	229339	36947	25924.000	75438.000	13656.000	31.264	55	4	11.967	27.350

Mesalina pasteuri	254193	52370	24154.666	69535.500	22686.000	15.189	4	2	20.800	33.217
Mesalina rubropunctata	254271.5	50154	24144.334	79142.000	22328.500	20.628	3.5	1	15.267	31.533
Mesalina saudiarabica	238702	45456	24461.666	69353.000	21452.000	19.792	38	6	18.517	34.317
Mesalina simoni	226964	36982	25424.000	71115.000	14880.000	30.195	109	8	12.517	26.583
Mesalina watsonana	222327	34724	24183.666	75092.000	14032.667	31.336	62	3	5.683	28.367
Nucras boulengeri	213604	55092	18364.000	55921.000	16947.666	10.166	229	32	21.767	24.517
Nucras intertexta	236243	45988	15430.334	67985.000	22577.000	17.709	25	268	14.450	23.983
Nucras lalandii	225424	42773	13696.000	68192.000	22727.000	21.473	42	387	10.150	18.850
Nucras livida	228277	36279.5	12093.167	77451.500	20767.000	33.393	51.5	64	11.467	21.633
Nucras taeniolata	213203	36102	11234.000	64656.000	21552.000	29.091	83	195	13.717	21.217
Nucras tessellata	245826	40845	23394.000	77301.000	20297.666	29.543	34	29	12.533	22.700
Omanosaura cyanura	257133	50216.5	20628.333	75871.500	18788.833	18.097	51.5	36	18.458	32.183
Omanosaura jayakari	255332	50369	20455.334	74829.000	21154.000	16.554	33	41	18.150	31.867
Ophisops beddomei	235554	55230	21520.334	68544.000	16311.333	16.585	2138	28	22.567	29.033
Ophisops elegans	215076	30878	24232.334	76167.000	12017.667	36.647	168	6	5.133	24.800
Ophisops jerdonii	217141	35980	16239.333	72251.000	18713.666	26.290	28	62	13.633	32.500
Ophisops leschenaultii	234257	55737	21073.000	63580.000	17074.334	9.266	912	137	24.500	27.433
Ophisops microlepis	NA	NA	NA	NA	NA	NA	NA	NA	NA	NA
Ophisops occidentalis	219642	32527	26058.500	75586.000	15866.500	35.646	120	23.5	10.058	24.350
Parvilacerta fraasii	213201	32347.5	25580.000	76740.000	9708.333	39.205	399	4	0.467	16.783
Parvilacerta parva	196490	24037	23592.666	76561.000	18395.666	45.669	136	89	-1.700	18.817
Pedioplanis burchelli	228767	37679	14124.000	72961.000	20105.666	32.646	68	108	7.767	17.967
Pedioplanis gaerdesi	234089.5	48646.5	16213.000	65059.000	21395.500	14.174	1	36	18.467	24.167
Pedioplanis inornata	247695	50833	17626.666	68184.000	22645.666	17.417	2	34	17.000	24.050
Pedioplanis laticeps	243178	39638	16400.334	82596.000	22082.666	31.244	34	39	10.800	23.850
Pedioplanis lineoocellata	243666.5	43926	16598.666	76914.500	23506.666	25.763	13	60.5	12.483	22.642
Pedioplanis namaquensis	249129	46513	16656.334	77783.000	23531.334	23.548	8	52	13.067	24.217
Pedioplanis undata	239824	50960	16986.666	68468.000	21539.666	13.330	3	108	16.692	23.617
Philochortus spinalis	251995	54606	24070.334	62998.000	18202.000	11.293	135	26	24.517	32.433
Phoenicolacerta cyanisparsa	214621	25752	25602.334	83710.000	8584.000	44.480	275	12	3.067	25.250
Phoenicolacerta kulzeri	215359	29573	25057.833	79899.000	9857.667	38.450	434	2	2.567	20.167
Phoenicolacerta laevis	216356	28623	26090.666	74404.000	9541.000	39.465	429	10	9.017	24.617
Phoenicolacerta troodica	221902	29732	27251.334	75567.000	9767.333	39.140	258	10	11.133	25.767
Podarcis bocagei	170810.5	19511	22288.334	60390.500	6027.000	48.266	501.5	126.5	7.650	18.417
Podarcis carbonelli	194897	23222	24305.334	70040.000	7189.000	45.409	332	72	10.633	19.300
Podarcis cretensis	193461	25072	25072.666	75218.000	8130.333	43.822	289	6	11.067	24.517
Podarcis erhardii	194390	29993	24966.000	74898.000	7791.667	44.968	223	10	10.650	24.133
Podarcis filfolensis	213256	36994	26226.666	71195.000	9093.667	39.900	262	7	13.150	25.183
Podarcis gaigeae	187475.5	28166	24911.167	74733.500	6936.833	49.083	186.5	24.5	10.167	24.350
Podarcis guadarramae	182074	20968	22798.334	67619.000	6933.333	48.202	399	93	5.600	19.067
Podarcis hispanicus	200931	27240	23259.666	76174.000	9729.000	41.554	149	53	5.167	22.617
Podarcis lilfordi	191193	32314	24794.000	65963.000	8947.667	44.344	189	39	11.533	23.817
Podarcis liolepis	179183	22817	21981.167	61878.000	11818.334	44.504	160	137	7.000	21.817
Podarcis melisellensis	166006.5	18144.5	22654.500	67963.500	7305.167	53.242	288.5	143.5	6.375	22.825
Podarcis milensis	192825	30415	24952.000	74856.000	7353.667	45.271	215	7	11.783	25.083
Podarcis muralis	138020	12343	9720.333	55616.500	9269.667	57.631	210	201	2.133	17.650
Podarcis peloponnesiacus	187453	22491	24031.666	72095.000	7119.667	45.782	374	39	7.167	23.250
Podarcis pityusensis	195060	26000	24709.334	66775.000	9379.667	42.595	145	35	12.050	24.700
Podarcis raffonei	191171	31475	24321.000	66329.000	9324.000	43.521	254	33	11.950	24.383
Podarcis siculus	176718	20877	23425.666	68696.000	8433.000	48.013	227	82	8.500	23.317
Podarcis tauricus	171707.5	19216	21007.167	67667.000	7908.167	50.098	156.5	110.5	3.592	21.850
Podarcis tiliguerta	184105	21906	24174.166	65422.000	8211.333	47.398	268	64.5	8.308	22.525

Podarcis vaucheri	211980	30446	25262.334	72911.500	9502.167	38.523	207	31.5	5.908	22.142
Podarcis virescens	197421	24813	23465.833	74999.000	8238.667	45.228	176	46	6.767	22.750
Podarcis waglerianus	197786.5	26634.5	24859.000	68253.500	9911.167	41.741	204.5	22	11.192	24.233
Poromera fordii	158575.5	32746	12891.333	45065.000	12609.667	14.108	186	496.5	22.308	24.642
Psammodromus algirus	183431.5	23410	23271.333	67297.500	11616.000	44.511	165	107	7.400	22.008
Psammodromus blanci	213373	31847.5	25239.833	75719.500	20271.500	35.480	105	52.5	5.808	23.583
Psammodromus edwarsianus	204794	27963.5	23896.000	72383.000	9297.667	41.569	155	35	7.925	23.558
Psammodromus hispanicus	189946	23126	23072.666	75475.000	8979.000	46.722	148	47	5.817	22.067
Psammodromus microdactylus	214320	31813	25028.334	70483.000	9870.667	35.476	164	20	8.767	24.367
Psammodromus occidentalis	191052	23075	23370.666	70199.000	7448.333	47.044	225	58	7.567	23.083
Pseuderemias smithii	238109.5	55562	18892.166	62532.500	19948.667	6.805	48	37.5	28.100	29.783
Scelarcis perspicillata	219721	33738	24435.167	72574.000	15528.667	33.651	206.5	36	5.958	21.667
Takydromus amurensis	156392.5	25896.5	8632.167	47256.500	15188.667	29.503	102.5	316.5	-4.183	20.500
Takydromus dorsalis	177903	27357	12827.333	62094.000	17466.000	32.050	484	637	17.750	27.017
Takydromus formosanus	191083	35873	12433.333	58714.000	19345.000	19.286	105	1188	16.883	26.583
Takydromus hsuehshanensis	176222	31091	10421.000	56108.000	18278.666	24.154	410	1304	3.150	13.933
Takydromus intermedius	139590	21246	7082.000	48211.000	16070.333	33.730	78	610	8.633	25.983
Takydromus luyeanus	189402	35700	11900.000	58380.000	18526.000	20.408	172	817	18.083	27.700
Takydromus sauteri	194691.5	37615.5	13201.167	58066.000	19319.667	18.666	99.5	1115	19.808	26.725
Takydromus septentrionalis	175888	28946.5	9762.167	59324.500	18405.167	28.890	165	527.5	5.950	26.992
Takydromus sexlineatus	214176.5	50745.5	18255.333	59769.000	16577.834	10.859	399	311	22.192	27.108
Takydromus smaragdinus	179465.5	28436	9484.500	59348.000	17773.000	29.699	363.5	656.5	17.125	28.100
Takydromus stejnegeri	189795.5	35905.5	12939.000	58844.500	19124.000	19.959	102	859	16.092	27.917
Takydromus sylvaticus	176735	29469	9971.333	60314.000	16561.334	29.013	211	575	5.367	26.267
Takydromus tachydromoides	160790	28089	9395.667	49455.000	17242.334	28.901	202	560	5.383	25.067
Takydromus toyamai	178956.5	27959.5	10695.834	59785.500	17448.334	30.593	440.5	603	18.508	28.258
Takydromus viridipunctatus	178950	29671	9945.333	60029.000	17384.334	28.887	472	807	16.150	27.283
Takydromus wolteri	171786.5	27711.5	9237.167	52791.000	16429.500	29.807	117.5	314	-0.242	23.517
Teira dugesii	190292.5	37507.5	21206.833	59532.000	9310.667	31.919	304.5	49	14.350	21.058
Timon kurdistanicus	208482	26874	24912.334	80813.000	11368.333	43.090	303	6	0.800	25.533
Timon lepidus	200885	26477	23402.167	75538.500	8313.000	43.317	219	41	7.192	22.817
Timon nevadensis	205547.5	28598	23330.334	76954.500	8713.333	40.328	167	37	4.850	22.450
Timon pater	205171	29303	25558.000	70779.000	9326.333	39.559	286	41	9.267	24.600
Timon princeps	225741	35988	24027.666	76139.500	14107.000	29.875	108	6	3.258	24.675
Timon tangitanus	220395	34121	25230.666	73633.000	17823.000	32.891	143	39	3.717	20.250
Tropidosaura cottrelli	224439	40142	12633.667	70953.000	24516.666	25.772	41	381	1.517	11.433
Tropidosaura essexi	217091	40616	12977.000	67321.000	22440.333	22.409	41.5	400.5	7.300	15.625
Tropidosaura gularis	218957	34112	10876.667	70711.500	21341.834	34.071	97	129	11.242	18.958
Tropidosaura montana	220246	36486	12376.667	71968.000	21910.334	29.334	102	199	9.567	18.250
Vhembelacerta rupicola	235198	47272	15757.333	68877.000	22959.000	16.908	45	402	12.467	20.817
Zootoca vivipara	116304	8682	14832.667	50281.000	3130.667	64.823	253	175	3.567	15.283

Supplementary Table 16. Pairs of lacertid sister species of sufficiently reliable taxonomy and distribution information to assess allopatric vs. sympatric occurrence. Total area and area overlap (in km^2) calculated from the GARD distribution ranges [64]. Species pairs with <10% range overlap are marked as non-allopatric in parentheses.

Species 1	Species 2	Clade	Allopatric	Total area	Overlap	Overlap/Total
Algyroides fitzingeri	*Algyroides marchi*	Lacertini	Yes	3.66E+10	0	0
Algyroides moreoticus	*Algyroides nigropunctatus*	Lacertini	Yes	9.72E+10	1.17E+09	1.20774
Apathya cappadocica	*Apathya yassujica*	Lacertini	Yes	3.25E+11	0	0
Darevskia caspica	*Darevskia chlorogaster*	Lacertini	(No)	1.55E+11	1.48E+10	9.54837
Darevskia caucasica	*Darevskia daghestanica*	Lacertini	No	1.77E+11	7.82E+10	44.11655
Darevskia clarkorum	*Darevskia mixta*	Lacertini	No	5.81E+10	1.06E+10	18.26396
Darevskia lindholmi	*Darevskia saxicola*	Lacertini	(No)	1.08E+11	9.52E+09	8.834881
Darevskia rudis	*Darevskia valentini*	Lacertini	(No)	4.68E+11	4.66E+10	9.952606
Darevskia schaekeli	*Darevskia steineri*	Lacertini	Yes	1.71E+10	0	0
Dinarolacerta montenegrina	*Dinarolacerta mosorensis*	Lacertini	(No)	3.33E+10	2.5E+09	7.515544
Iberolacerta aranica	*Iberolacerta bonnali*	Lacertini	Yes	2.41E+10	59947256	0.248425
Iberolacerta galani	*Iberolacerta martinezricai*	Lacertini	Yes	5.14E+09	0	0
Iranolacerta brandtii	*Iranolacerta zagrosica*	Lacertini	Yes	9.96E+10	1.07E+09	1.075225
Lacerta bilineata	*Lacerta viridis*	Lacertini	Yes	2.18E+12	6.14E+09	0.281576
Lacerta pamphylica	*Lacerta trilineata*	Lacertini	Yes	6.1E+11	9.75E+08	0.159907
Parvilacerta fraasii	*Parvilacerta parva*	Lacertini	Yes	3.9E+11	0	0
Phoenicolacerta cyanisparsa	*Phoenicolacerta laevis*	Lacertini	(No)	1.26E+11	8.63E+09	6.870341
Podarcis raffonei	*Podarcis waglerianus*	Lacertini	No	NA	NA	NA
Podarcis bocagei	*Podarcis guadarramae*	Lacertini	No	1.46E+11	4.8E+10	32.92972
Podarcis carbonelli	*Podarcis virescens*	Lacertini	(No)	2.12E+11	1.91E+10	9.027807
Podarcis erhardii	*Podarcis peloponnesiacus*	Lacertini	(No)	1.65E+11	1.38E+10	8.343689
Podarcis hispanicus	*Podarcis vaucheri*	Lacertini	Yes	9.27E+11	4.34E+08	0.046812
Podarcis lilfordi	*Podarcis pityusensis*	Lacertini	Yes	5.09E+09	39933697	0.784966
Podarcis melisellensis	*Podarcis milensis*	Lacertini	Yes	5.12E+10	0	0
Scelarcis perspicillata	*Teira dugesii*	Lacertini	Yes	NA	NA	NA

Takydromus dorsalis	*Takydromus sylvaticus*	Lacertini	Yes	1.59E+10	0	0
Takydromus septentrionalis	*Takydromus toyamai*	Lacertini	Yes	2.48E+12	0	0
Timon pater	*Timon tangitanus*	Lacertini	Yes	3.33E+11	39677660	0.011919
Acanthodactylus aegyptius	*Acanthodactylus longipes*	Eremiadini	Yes	8.11E+12	1.01E+09	0.012387
Acanthodactylus blanfordii	*Acanthodactylus schmidti*	Eremiadini	Yes	2.94E+12	1.87E+10	0.633847
Acanthodactylus boskianus	*Acanthodactylus schreiberi*	Eremiadini	Yes	1.58E+13	0	0
Acanthodactylus cantoris	*Acanthodactylus masirae*	Eremiadini	Yes	1.44E+12	0	0
Acanthodactylus dumerilii	*Acanthodactylus senegalensis*	Eremiadini	No	2.61E+12	5.38E+11	20.6183
Acanthodactylus felicis	*Acanthodactylus opheodurus*	Eremiadini	Yes	1.84E+12	6.11E+10	3.327132
Acanthodactylus gongrorhynchatus	*Acanthodactylus tilburyi*	Eremiadini	Yes	2.59E+11	7.34E+09	2.830742
Acanthodactylus khamirensis	*Acanthodactylus micropholis*	Eremiadini	Yes	5.33E+11	9996281	0.001874
Acanthodactylus orientalis	*Acanthodactylus robustus*	Eremiadini	No	4.07E+11	8.71E+10	21.413
Adolfus alleni	*Adolfus jacksoni*	Eremiadini	Yes	3.91E+11	9.96E+09	2.544559
Congolacerta asukului	*Congolacerta vauereselli*	Eremiadini	Yes	1.43E+11	1.99E+09	1.387175
Eremias argus	*Eremias brenchleyi*	Eremiadini	No	4.29E+12	2E+12	46.45429
Eremias arguta	*Eremias intermedia*	Eremiadini	No	5.49E+12	1.09E+12	19.83387
Eremias grammica	*Eremias pleskei*	Eremiadini	Yes	1.9E+12	0	0
Eremias montana	*Eremias strauchi*	Eremiadini	Yes	3.72E+11	0	0
Eremias multiocellata	*Eremias przewalskii*	Eremiadini	No	4.78E+12	2.3E+12	48.10011
Eremias papenfussi	*Eremias suphani*	Eremiadini	Yes	2.02E+10	0	0
Gastropholis prasina	*Gastropholis vittata*	Eremiadini	(No)	1.91E+10	1.93E+09	10.12368
Heliobolus lugubris	*Heliobolus spekii*	Eremiadini	Yes	3.81E+12	0	0
Holaspis guentheri	*Holaspis laevis*	Eremiadini	Yes	2.38E+12	0	0
Meroles anchietae	*Meroles ctenodactylus*	Eremiadini	Yes	1.57E+11	0	0
Meroles cuneirostris	*Meroles micropholidotus*	Eremiadini	No	1.07E+11	3.24E+10	30.21211
Meroles knoxii	*Meroles suborbitalis*	Eremiadini	(No)	7.05E+11	7.03E+10	9.968187
Mesalina brevirostris	*Mesalina saudiarabica*	Eremiadini	Yes	NA	NA	NA
Mesalina adramitana	*Mesalina balfouri*	Eremiadini	Yes	6.74E+11	0	0
Mesalina bahaeldini	*Mesalina guttulata*	Eremiadini	Yes	1.13E+13	1.61E+10	0.142792
Mesalina olivieri	*Mesalina simoni*	Eremiadini	Yes	2.72E+12	1.8E+08	0.006632
Nucras intertexta	*Nucras taeniolata*	Eremiadini	Yes	1.09E+12	0	0

Nucras livida	*Nucras tessellata*	Eremiadini	(No)	7.38E+11	4.91E+10	6.651568
Omanosaura cyanura	*Omanosaura jayakari*	Eremiadini	No	3.69E+10	2.02E+10	54.7875
Ophisops beddomei	*Ophisops jerdonii*	Eremiadini	(No)	2.9E+12	2.93E+11	10.09995
Ophisops elegans	*Ophisops occidentalis*	Eremiadini	Yes	2.72E+12	7.92E+10	2.908367
Pedioplanis gaerdesi	*Pedioplanis inornata*	Eremiadini	Yes	3.17E+11	0	0
Tropidosaura cottrelli	*Tropidosaura essexi*	Eremiadini	No	1.15E+10	2.39E+09	20.74445
Tropidosaura gularis	*Tropidosaura montana*	Eremiadini	No	1.28E+11	6.09E+10	47.54509
Gallotia bravoana	*Gallotia simonyi*	Gallotiinae	Yes	3.87E+08	0	0
Gallotia caesaris	*Gallotia galloti*	Gallotiinae	Yes	3.53E+09	10567185	0.299114
Psammodromus edwarsianus	*Psammodromus hispanicus*	Gallotiinae	(No)	NA	NA	NA
Psammodromus blanci	*Psammodromus microdactylus*	Gallotiinae	Yes	1.89E+11	0	0

Supplementary Table 17. Node numbers of tree in Supplementary Fig. 16, corresponding to the tree displaying results of ancestral character state reconstructions for yearly hours >30°C (as visualized in Fig. 2). The table shows for each node in the tree the node number (as in Supplementary Fig. 16), the reconstructed ancestral state for yearly hours >30°C, as well as the lower and upper confidence interval for this reconstructed ancestral bioclimatic variable.

node number	hours >30°C	CI_lower	CI_upper
233	2019.58	1732.52	2306.63
234	2218.65	2059.68	2377.63
235	2393.46	2264.82	2522.11
236	2468.04	2350.34	2585.73
237	2594.27	2480.72	2707.82
238	2596.35	2481.88	2710.81
239	2596.35	2481.88	2710.81
240	2579.3	2462.33	2696.26
241	2677.9	2541.56	2814.24
242	2580.29	2423.19	2737.38
243	2693.58	2570.03	2817.13
244	2693.31	2585.39	2801.23
245	2684.31	2585.99	2782.63
246	2661.72	2570.4	2753.03
247	2751.74	2659.91	2843.57
248	2466.02	2387.32	2544.71
249	2497.89	2422.65	2573.14
250	2722.92	2648.01	2797.83
251	3289.62	3189.41	3389.83
252	2356.47	2252.92	2460.02
253	2724.38	2630.49	2818.28
254	2686.88	2593.45	2780.32
255	2866.56	2707.76	3025.36
256	2686.51	2513.15	2859.86
257	1960.9	1805.34	2116.46
258	4036.45	3878.62	4194.27
259	1687.91	1530.94	1844.89
260	1436.92	1322.01	1551.84
261	1410.82	1303.62	1518.02
262	1365.21	1268.57	1461.85
263	1372.56	1275.3	1469.82
264	1342.32	1239.98	1444.65
265	1280.89	1184.25	1377.53
266	1227.38	1127.98	1326.78
267	1202.41	1098.24	1306.58
268	1286.57	1181.5	1391.65
269	1461.75	1339.31	1584.2
270	1236.23	1129.81	1342.65
271	1002.23	890.52	1113.94
272	871.61	766.2	977.03
273	1260.85	1144.45	1377.25
274	1385.47	1254.16	1516.79
275	2666.36	2530.49	2802.24
276	2780.15	2642.34	2917.96
277	3166.33	3070.14	3262.53
278	3243.35	3151.99	3334.71
279	3397.92	3314.31	3481.54
280	3384.61	3299.11	3470.12
281	3177.83	3070.88	3284.79
282	2957.38	2855.76	3059.01
283	3249.7	3169.4	3330
284	3394.72	3290.38	3499.06
285	3922.61	3811.21	4034.01
286	3638.16	3550.76	3725.56
287	3723.48	3633.29	3813.67
288	3766.81	3672.52	3861.1
289	3721.2	3621.99	3820.4
290	3890.26	3777.87	4002.65
291	3312.32	3196.57	3428.06
292	2628.89	2515.41	2742.38
293	2428.79	2313.35	2544.22
294	2699.65	2566.23	2833.06
295	2747.31	2620.63	2873.98
296	2712.41	2608.39	2816.43
297	2450.75	2353.44	2548.06
298	2116.81	2030.57	2203.04
299	3650.92	3578.73	3723.11
300	2511.33	2382	2640.66
301	2418.04	2291.84	2544.25
302	2360.27	2233.54	2486.99
303	1961.97	1855.5	2068.44
304	1921.79	1823.96	2019.63
305	1833.1	1743.58	1922.62
306	1774.49	1688.3	1860.67
307	1680.72	1606.33	1755.1
308	2315.8	2178.92	2452.68
309	2189.94	2053.27	2326.61

310	1624.67	1501.51	1747.84
311	1474.8	1397.64	1551.96
312	2794.84	2660.07	2929.62
313	2918.66	2777.23	3060.08
314	2887.68	2743.42	3031.94
315	2790.57	2639.99	2941.16
316	2488.89	2320.14	2657.64
317	1670.51	1529.17	1811.85
318	2594.56	2436.86	2752.26
319	3433.18	3266.52	3599.83
320	3409.19	3227.06	3591.31
321	3275.83	3092.13	3459.54
322	2506.49	2384.78	2628.2
323	2364.35	2238.55	2490.15
324	2287.27	2148.96	2425.57
325	2408.46	2262.51	2554.42
326	2385.31	2230.97	2539.64
327	2446.27	2297.34	2595.2
328	2541.49	2428.64	2654.34
329	2473.61	2401.35	2545.87
330	2440.26	2287.97	2592.55
331	1925.83	1776.04	2075.63
332	1606.35	1449.13	1763.57
333	1400.57	1257.32	1543.82
334	1457.19	1304.9	1609.48
335	2362.64	2234.36	2490.91
336	2375.54	2242.71	2508.37
337	2405.52	2255.68	2555.36
338	2340.67	2199.33	2482.02
339	2360.82	2232.63	2489.01
340	2367.05	2238.99	2495.1

341	2338.55	2204.81	2472.28
342	2438.47	2305.01	2571.93
343	2370.41	2238.22	2502.61
344	2297.73	2156.04	2439.42
345	2696.71	2551.35	2842.07
346	2506.84	2339.48	2674.2
347	2191.71	2040.81	2342.62
348	2020.7	1872.41	2168.98
349	1868.81	1739.01	1998.6
350	2128.67	1981.97	2275.37
351	3155.4	2989.77	3321.03
352	3237.37	3068.63	3406.11
353	3198.34	3034.98	3361.7
354	1373.79	1245.41	1502.17
355	1288.22	1184.7	1391.73
356	1303.04	1211.05	1395.02
357	1329.58	1243.49	1415.66
358	1337.01	1253.54	1420.47
359	1354.4	1270.45	1438.36
360	1376.27	1289.31	1463.24
361	1293.26	1170.63	1415.88
362	1160.41	1053.68	1267.13
363	1140.15	1046.43	1233.86
364	1144.85	1064.21	1225.48
365	1184.03	1106.97	1261.09
366	1209.92	1137.02	1282.82
367	1071.39	998.23	1144.55
368	1032.36	961.08	1103.65
369	923.57	850.94	996.2
370	1177.22	1122.01	1232.42
371	1292.3	1216.11	1368.5

372	1574.64	1501.49	1647.78
373	1643.28	1572.52	1714.03
374	1272.96	1187.9	1358.02
375	1331.86	1259.2	1404.51
376	1409.96	1341.11	1478.81
377	1032.25	939.34	1125.17
378	759.26	688.98	829.54
379	1002.43	920.98	1083.89
380	802.91	754.45	851.38
381	1207.8	1074.92	1340.69
382	1924.26	1811.35	2037.17
383	1934.04	1834.2	2033.87
384	1935.06	1835.98	2034.14
385	1777.43	1667.92	1886.94
386	1736.11	1630.9	1841.33
387	1475.57	1381.08	1570.06
388	1538.38	1448.36	1628.39
389	1828.68	1766.95	1890.41
390	1828.68	1766.95	1890.41
391	1987.74	1881.31	2094.17
392	2001.85	1895.02	2108.69
393	2247.36	2138.59	2356.13
394	2166.78	2063.05	2270.51
395	1936.84	1822.57	2051.11
396	1341.68	1242.43	1440.93
397	1293.79	1176.4	1411.18
398	1070.25	957.7	1182.8
399	996.64	894.06	1099.22
400	956.85	864.55	1049.15
401	951.6	860.12	1043.09
402	1235.15	1158.01	1312.3

403	1293.01	1220.23	1365.78
404	875.39	795.95	954.84
405	1327.27	1202.79	1451.76
406	1234.78	1133.35	1336.22
407	1238.63	1145.27	1331.99
408	1270.97	1196.81	1345.13
409	1417.69	1297.07	1538.3
410	1422.25	1274.59	1569.91
411	1284.69	1189.13	1380.24
412	1272.48	1174.36	1370.6
413	673	555.65	790.36
414	612.05	506.9	717.2
415	642.3	539.8	744.81
416	641.64	581.95	701.32
417	652.52	594.23	710.81
418	370.64	287.08	454.2
419	388.45	310.37	466.53
420	1261.76	1134.62	1388.91
421	1431.89	1350.47	1513.3
422	1461.06	1383.38	1538.74
423	1092.12	969.51	1214.72
424	1498.04	1370.71	1625.37
425	1623.53	1538.83	1708.23
426	1664.22	1587.85	1740.59
427	1260.5	1145.87	1375.12
428	1149.48	1028.67	1270.29
429	1174.62	1055.1	1294.14
430	1142.97	1008.21	1277.72
431	1207.99	1081.92	1334.06
432	540.3	436.13	644.47
433	1042.78	908.23	1177.32
434	997.17	857.2	1137.15
435	1187.6	1047.97	1327.23
436	1260.68	1156.65	1364.7
437	1320.81	1222.84	1418.77
438	1329.05	1224.28	1433.81
439	1349.31	1252.61	1446
440	1358.5	1263.35	1453.65
441	1381.73	1279.9	1483.55
442	1355.29	1249.8	1460.78
443	1443.73	1354.71	1532.75
444	1697.45	1621.75	1773.15
445	1707.58	1634.37	1780.8
446	1221.98	1115.71	1328.26
447	1067.71	956.56	1178.86
448	1086.62	1012.89	1160.35
449	1087	1023.15	1150.85
450	1065.33	1002.79	1127.87
451	929.46	860.88	998.04
452	1082.12	1016.04	1148.21
453	1127.27	1057.84	1196.71
454	1499.56	1311.84	1687.29
455	1590.52	1443.22	1737.82
456	1602.55	1469.49	1735.6
457	1358.3	1274.29	1442.3
458	1261.09	1191.58	1330.61
459	1345.04	1189.36	1500.73
460	1313.11	1177.8	1448.41
461	1083.83	973.95	1193.7
462	1084.75	988.05	1181.45
463	1388.48	1254.37	1522.6

Supplementary References

1. Müller, J., Hipsley, C. A., Head, J. J., Kardjilov, N., Hilger, A., Wuttke, M. & Reisz, R. R. Eocene lizard from Germany reveals amphisbaenian origins. *Nature* **473**, 364–367 (2011).
2. Lemmon, A. R., Emme, S. & Lemmon, E.M.. Anchored hybrid enrichment for massively high-throughput phylogenetics. *Syst. Biol.* **61**, 721–744 (2012).
3. Prum, R. O., Berv, J. S., Dornburg, A., Field, D. J., Townsend, J. P., Lemmon, E. C. & Lemmon, A. R. A fully resolved, comprehensive phylogeny of birds (Aves) using targeted next generation DNA sequencing. *Nature* **526**, 569–573 (2015).
4. Meyer, M. & Kircher, M. Illumina sequencing library preparation for highly multiplexed target capture and sequencing. *Cold Spring Harb Protoc.* **6**, pdb.prot5448 (2011).
5. Ruane, S., Raxworthy, C. J., Lemmon, A. R., Lemmon, E. C. & Burbrink, F. T. Comparing large anchored phylogenomic and small molecular datasets for species tree estimation: an empirical example using Malagasy pseudoxyrhophiine snakes. *BMC Evol. Biol.* **15**, 221 (2015).
6. Tucker, D. B., Colli, G. R., Giugliano, L. G., Hedges, S. B., Hendry, C. R., Moriarty Lemmon, E., Lemmon, A. R., Sites, J. W. Jr & Pyron, R. A. Phylogenomic analysis of tegus and whiptails (Teiidae: Squamata), with a revised taxonomy and a new genus from the West Indies. *Mol Phylogenet Evol.* **103**, 75–84 (2016).
7. Rokyta, D. R., Lemmon, A. R., Margres, M. J. & Aronow, K. The venom-gland transcriptome of the eastern diamondback rattlesnake (*Crotalus adamanteus*). *BMC Genomics* **13**, 312 (2012).
8. Hamilton, C. A., Lemmon, A.R., Moriarty Lemmon, E., Bond, J.E. Expanding Anchored Hybrid Enrichment to resolve both deep and shallow relationships within the spider Tree of Life. *BMC Evol. Biol.* **16**, 212 (2016).
9. Katoh, K. & Standley, D. M. MAFFT Multiple Sequence Alignment Software Version 7: Improvements in performance and usability. *Mol. Biol. Evol*. **30**, 772–780 (2013).
10. Kearse, M., Moir, R., Wilson, A., Stones-Havas, S., Cheung, M., Sturrock, S., Buxton, S., Cooper, A., Markowitz, S., Duran, C., et al. Geneious Basic: an integrated and extendable desktop software platform for the organization and analysis of sequence data. *Bioinformatics* **28**, 1647–1649 (2012).
11. Bolger, A.M., Lohse, M., Usadel B. Trimmomatic: a flexible trimmer for Illumina sequence data. *Bioinformatics*. **30**, 2114–21120 (2014).
12. Kopylova, E., Noé, L., Touzet, H.. SortMeRNA: fast and accurate filtering of ribosomal RNAs in metatranscriptomic data. *Bioinformatics* **28**, 3211–3217 (2012).
13. Grabherr, M.G., Haas, B.J., Yassour, M., Levin, J.Z., Thompson, D.A., Amit, I., Adiconis, X., Fan, L., Raychowdhury, R., Zeng, Q., Chen, Z., Mauceli, E., Hacohen, N., Gnirke, A., Rhind, N., di Palma, F., Birren, B.W., Nusbaum, C., Lindblad-Toh, K., Friedman, N., Regev, A.. Full-length transcriptome assembly from RNA-Seq data without a reference genome. *Nat Biotechnol.* **29**, 644–652 (2011).
14. Haas, B. J., Papanicolaou, A., Yassour, M., Grabherr, M., Blood, P. D., Bowden, J., Couger, M. B., Eccles, D., Li, B., Lieber, M., MacManes, M. D., Ott, M., Orvis, J., Pochet, N., Strozzi, F., Weeks, N., Westerman, R., William, T., Dewey, C.N., Henschel, R., LeDuc, R. D., Friedman, N. & Regev, A. De novo transcript sequence reconstruction from RNA-Seq: reference generation and analysis with Trinity. *Nat. Protoc*. **8**, 1494–1512 (2013).
15. Irisarri, I. *et al.* Phylotranscriptomic consolidation of the jawed vertebrate timetree. *Nat Ecol Evol* **1**, 1370–1378 (2017).
16. Stamatakis, A. (2014): RAxML version 8: a tool for phylogenetic analysis and post-analysis of large phylogenies. *Bioinformatics* **30**, 1312–1313 (2010).
17. Nguyen, L.-T., Schmidt, H. A., von Haeseler, A. & Minh, B. Q. IQ-TREE: a fast and effective stochastic algorithm for estimating maximum-likelihood phylogenies. *Mol. Biol. Evol.* **32**, 268–274 (2015).
18. Hoang, D. T., Chernomor, O., Haeseler, A.v., Minh, B. Q. & Vinh, L. S. UFBoot2: Improving the ultrafast bootstrap approximation. *Mol. Biol. Evol.* **35,** 518–522 (2017).
19. Kubatko, L. S. & Degnan, J. H. Inconsistency of phylogenetic estimates from concatenated data under coalescence. *Syst. Biol.* **56**, 17–24 (2007).

20. Philippe, H., Brinkmann, H., Lavrov, D.V., Littlewood, D.T.J., Manuel, M., Wörheide, G. & Baurain, D. Resolving difficult phylogenetic questions: why more sequences are not enough. *PLoS Biol*. **9**, e1000602 (2011).
21. Mirarab, S. & Warnow, T. ASTRAL-II: coalescent-based species tree estimation with many hundreds of taxa and thousands of genes. *Bioinformatics* **31**, i44–52 (2015).
22. Sayyari, E., Mirarab, S. Fast coalescent-based computation of local branch support from quartet frequencies. *Mol. Biol. Evol.* **33**, 1654–1668 (2016).
23. Gervais, P. Zoologie et Paléontologie françaises (Animaux vertébrés). 3 volumes. Paris (1848).
24. Čerňanský, A., Bolet, A., Müller, J., Rage, J.-C., Augé, M. & Herrel, A. A new exceptionally preserved specimen of *Dracaenosaurus* (Squamata, Lacertidae) from the Oligocene of France as revealed by micro-computed tomography. *J. Vert. Paleontol.* **37**, e1384738. (2017).
25. Boulenger, G. A. A revision of the lizards of the genus *Nucras*, Gray. *Annals of the South African Museum* **13**, 95–215 (1917).
26. Borsuk-Białynicka, M., Lubka, M. & Böhme, W. A lizard from Baltic amber (Eocene) and the ancestry of the crown group lacertids. *Acta Palaeontologica Polonica* **44**, 349–382 (1999).
27. Gerhardt, K. *Ophisaurus ulmensis* n. sp. aus dem Untermiozän von Ulm a. *Jahreshefte des Vereins für vaterländische Naturkunde in Württemberg* **59**, 67–71 (1903).
28. Čerňanský, A. & Auge, M. L. New species of the genus *Plesiolacerta* (Squamata: Lacertidae) from the Upper Oligocene (MP28) of southern Germany and a revision of the type species *Plesiolacerta lydekkeri*. *Palaeontology* **56**, 79–94 (2013).
29. Hoffstetter, R. Sur les restes de Sauria du Nummulitique européen rapportés à la famille Iguanidae. *Bull. Mus. nat. Hist. Nat. Paris* **14**, 233–240 (1942).
30. Čerňanský, A. Earliest world record of green lizards (Lacertilia, Lacertidae) from the Lower Miocene of Central Europe. *Biologia* **65**, 737–741 (2010).
31. Filhol, H. (1877). *Recherches sur les phosphorites du Quercy: étude des fossiles qu'on y rencontre et spécialement des mammifères* (Vol. 2). G. Masson.
32. Augé, M. L. & Hervet S. Fossil lizards from the locality of Gannat (late Oligocene–early Miocene, France) and a revision of the genus *Pseudeumeces* (Squamata, Lacertidae). *Palaeobiodiv. Palaeoenviron.* **89**, 191 (2009).
33. R Core Team. R: A language and environment for statistical computing. R Foundation for Statistical Computing, Vienna, Austria. URL https://www.R-project.org/ (2017).
34. Revell, L. J. phytools: An R package for phylogenetic comparative biology (and other things). *Methods Ecol. Evol.* **3**, 217–223 (2012).
35. Paradis E., Claude J. & Strimmer K. APE: analyses of phylogenetics and evolution in R language. Bioinformatics **20**, 289–290 (2004).
36. Goloboff, P.A., Catalano, S.A. TNT version 1.5, including a full implementation of phylogenetic morphometrics. *Cladistics* **32**, 221–238 (2016).
37. Jones, M. E. H., Anderson C. L., Hipsley C. A., Müller J., Evans S. & Schoch R. Integration of molecules and new fossils supports a Triassic origin for Lepidosauria (lizards, snakes, and tuatara). *BMC Evol. Biol.* **13**, 208 (2013).
38. Kaasalainen, U., Schmidt, A.R. & Rikkinen, J. Diversity and ecological adaptations in Palaeogene lichens. *Nat. Plants* **3**, 17049 (2017).
39. Augé, M. L. Évolution des lézards du Paléogène en Europe. Mémoires du Museum national d'Histoire naturelle Tome 192, 369 pp (2005).
40. Čerňanský A. & Joniak P. Nové nálezy jašteríc (Sauria, Lacertidae) z neogénnych sedimentov Slovenska a Českej republiky. *Acta Geologica Slovaca* **1**, 57–64 (2009).
41. Hipsley C. A., Miles, D. B. & Müller, J. Morphological disparity opposes latitudinal diversity gradient in lacertid lizards. *Biol. Lett.* **10**, 20140101 (2014).
42. Agustí, J., Blain, H. A., Furió, M., De Marfá, R. & Santos-Cubedo, A. The early Pleistocene small vertebrate succession from the Orce region (Guadix-Baza Basin, SE Spain) and its bearing on the first human occupation of Europe. *Quaternary International* **223–224**,162–169 (2010).
43. Smith, S. A. & O'Meara, B. C. treePL: divergence time estimation using penalized likelihood for large phylogenies. *Bioinformatics* **28**, 2689–2690 (2012).

44. Hipsley, C. A., Himmelmann L., Metzler D. & Müller, J. Integration of Bayesian molecular clock methods and fossil-based soft bounds reveals early Cenozoic colonization of African lacertid lizards. *BMC Evol. Biol.* **9**, 151 (2009).
45. Mulcahy, D. G., Noonan, B. P., Moss, T., Townsend, T. M., Reeder, T. W., Sites Jr, J. W. & Wiens, J. J. Estimating divergence dates and evaluating dating methods using phylogenomic and mitochondrial data in squamate reptiles. *Mol. Phylogenet. Evol.* **65**, 974–991 (2012).
46. Zheng, Y. & Wiens, J. J. Combining phylogenomic and supermatrix approaches, and a time-calibrated phylogeny for squamate reptiles (lizards and snakes) based on 52 genes and 4162 species. *Mol. Phylogenet. Evol.* **94**, 537–547 (2016).
47. Pyron, R. A. & Burbrink, F. T. Early origin of viviparity and multiple reversions to oviparity in squamate reptiles. *Ecol. Lett.* **17**, 13–21 (2014).
48. García-Muñoz, E., Carretero. M. A. Comparative ecophysiology of two sympatric lizards. Laying the groundwork for mechanistic distribution models. *Acta Herpetol.* **8**, 123–128 (2013).
49. Grigg, G. C., Drane, C. R. & Courtice, G. P. Time constants of heating and cooling in the Eastern Water Dragon *Physignathus lesueurii* and some generalisations about heating and cooling in reptiles. J. Therm. Biol. **4**, 95–103 (1979).
50. Kirchhof, S. *et al.* Thermoregulatory behavior and high thermal preference buffer impact of climate change in a Namib Desert lizard. *Ecosphere* **8**, e02033 (2017).
51. Carretero, M. A. Measuring body temperatures in small lacertids: Infrared vs. contact thermometers. *Basic Appl. Herpetol.* **26**, 99-105 (2012).
52. Barroso, F. M., Carretero, M.A., Silva, F. & Sannolo, M. Assessing the reliability of thermography to infer internal body temperatures of lizards. *J. Thermal Biol.* **62**, 90–96 (2016).
53. Carneiro, D., García-Muñoz, E., Žagar, A., Pafilis, P. & Carretero, M. A. Is ecophysiology congruent with the present-day relictual distribution of a lizard group? Evidence from preferred temperatures and water loss rates. *Herp. J.* **27**, 47–56 (2017).
54. Žagar, A., Carretero, M. A., Marguč, D., Simčič, T., Vrezec, A. A metabolic syndrome in terrestrial ectotherms with different elevational and distribution patterns. *Ecography*, https://doi.org/10.1111/ecog.03411 (2018).
55. Sillero, N., Campos, J., Bonardi, A., Corti, C., Creemers, R., Crochet, P.-A., Crnobrnja Isailovic, J., Denoël, M., Ficetola, G. F., Gonçalves, J., Kuzmin, S., Lymberakis, P., de Pous, P., Rodríguez, A., Sindaco, R., Speybroeck, J., Toxopeus, B., Vieites, D. R. & Vences, M. Updated distribution and biogeography of amphibians and reptiles of Europe. *Amphibia-Reptilia* **35**, 1–31 (2014).
56. Kottek, M., Grieser, J., Beck, C., Rudolf, B., & Rubel, F. World Map of the Köppen-Geiger climate classification updated. *Meteorol. Z.* **15**, 259–263 (2006).
57. Fick, S. E. & Hijmans, R. J. WorldClim 2: new 1-km spatial resolution climate surfaces for global land areas. *Int. J. Climatol.* **37**, 4302–4315 (2017).
58. Kriticos, D.J., Jarošik, V., Ota, N. Extending the suite of Bioclim variables: a proposed registry system and case study using principal components analysis. *Methods in Ecology and Evolution* **5**, 956–960 (2014).
59. Hijmans, R. J. raster: Geographic Data Analysis and Modeling. R package version 2.5-8. https://CRAN.R-project.org/package=raster (2016).
60. Kearney, M. R., Isaac, A. P. & Porter, W. P. Microclim: Global estimates of hourly microclimate based on long-term monthly climate averages. *Sci Data* **1,** 140006 (2014).
61. Milto, K.D. *Zootoca vivipara* (Comm,on Lizard). Abnormal Activity. *Herp. Rev.* **45**, 511 (2014)
62. Arribas OJ. Activity, microhabitat selection and thermal behavior of the Pyrenean Rock Lizards *Iberolacerta aranica* (Arribas, 1993), *I. aurelioi* (Arribas, 1994) and *I. bonnali* (Lantz, 1927) (Squamata: Sauria: Lacertidae). *Herpetozoa* **23**, 3–23 (2010).
63. Rangel, T. F. L. V. B, Diniz-Filho, J. A. F & Bini, L. M. SAM: a comprehensive application for Spatial Analysis in Macroecology. *Ecography* **33**, 46–50 (2010).
64. Roll, U. *et al.* The global distribution of tetrapods reveals a need for targeted reptile conservation. *Nature Ecologyand Evolution* **1**, 1677–1682 (2017).
65. Brown, J. L., Cameron, A., Yoder, A. D. & Vences, M. A necessarily complex model to explain the biogeography of the amphibians and reptiles of Madagascar. *Nat. Comm.* **10**, 5 (2014).

66. Blomberg, S. P., Garland, T., Jr & Ives, A. R. Testing for phylogenetic signal in comparative data: behavioral traits are more labile. *Evolution* **57**, 717–745 (2003).
67. Revell, L.J., Harmon, L.J. & Collar, D.C. Phylogenetic signal, evolutionary process, and rate. Syst. Biol. 57, 591-601 (2008).
68. Cooper, N., Jetz, W. & Freckleton, R. P. Phylogenetic comparative approaches for studying niche conservatism. *J. Evol. Biol.*, **23**, 2529–2539 (2010).
69. Kuhn, M. Building predictive models in R using the caret package. *J Stat Software* **28**, 1–26 (2008)
70. Harmon, L.J., Schulte, J. A., Losos, J. B. & Larson, A. Tempo and mode of evolutionary radiation in iguanian lizards. *Science* **301**, 961–964 (2003).
71. Foote, M. The evolution of morphological diversity. *Annu. Rev. Ecol. Evol. Syst*. **28**, 129–152 (1997).
72. Slater, G. J., Price, S. A., Santini, F. & Alfaro, M. A. Diversity vs disparity and the evolution of modern cetaceans. *Proc. Roy. Soc.* B **277**, 3097–3104 (2010).
73. Harmon, L., Weir, J., Brock, C., Glor, R. & Challenger, W. Geiger: investigating evolutionary radiations. *Bioinformatics* **24**, 129–131 (2008).
74. Felsenstein, J. Phylogenies and the comparative method. *Am. Nat.* **126**, 1–25 (1985).
75. McPeek M. A. Testing hypotheses about evolutionary change on single branches of a phylogeny using evolutionary contrasts. *Am. Nat.* **45**, 686–703 (1995).
76. Freckleton, R. P. & Harvey, P. H. Detecting non-Brownian trait evolution in adaptive radiations. PLoS Biol. **4**, e373 (2006).
77. Ho, L, Ané, C. A linear-time algorithm for Gaussian and non-Gaussian trait evolution models. *Syst Biol.* **63**, 397–408 (2014).
78. Morlon, H., Lewitus, E., Condamine, F. L., Manceau, M., Clavel, J. & Drury, J. RPANDA: an R package for macroevolutionary analyses on phylogenetic trees. *Methods Ecol. Evol.* **7,** 589–597 (2016).
79. Morlon, H., Parsons, T. L. & Plotkin, J. B. Reconciling molecular phylogenies with the fossil record. *Proc. Natl. Acad. Sci. U.S.A.* **108**, 16327–16332 (2011).
80. Epstein, S., Buchsbaum, R., Lowenstam, H.A. & Urey, H.C. Revised carbonate-water isotopic temperature scale. *Geol. Soc. Am. Bull.* **64**, 1315–1326 (1953).
81. Zachos, J. C., Dickens, G. R. & Zeebe, R. E. An early Cenozoic perspective on greenhouse warming and carbon-cycle dynamics Nature **451**, 279–283 (2008).
82. Condamine, F.L., Rolland, J. & Morlon, H. Macroevolutionary perspectives to environmental change. *Ecol. Lett.* **16**, 72–85 (2013).
83. Rabosky, D. L. Automatic detection of key innovations, rate shifts, and diversity-dependence on phylogenetic trees. PloS ONE **9**, e89543 (2014).
84. Tong, K. J., Duchêne, D. A., Duchêne, S., Geoghegan, J. L. & Ho, S. Y. W. A comparison of methods for estimating substitution rates from ancient DNA sequence data. *BMC Evol. Biol.* **18**, 70 (2018).
85. Blueweiss, L., Fox, H., Kudzma, V., Nakashima, D., Peters, R. & Sams, S. Relationships between body size and some life history parameters. *Oecologia* **37**, 257–272 (1978).
86. Bromham, L. Molecular clocks in reptiles: life history influences rate of molecular evolution. *Mol. Biol. Evol.* **19**, 302–309 (2002).
87. Kumar, S., Stecher, G. & Tamura, K. MEGA7: Molecular Evolutionary Genetics Analysis Version 7.0 for bigger datasets. *Mol. Biol. Evol.* **33**, 1870–1874 (2016).
88. Arnold, E. N. Towards a phylogeny and biogeography of the Lacertidae: relationships within an Old-World family of lizards derived from morphology. *Bull. British Mus. nat. Hist. (Zool.), London* **55**, 209–257 (1989).
89. Arnold, E. N., Arribas, Ó. & Carranza, S. Systematics of the Palaearctic and Oriental lizard tribe Lacertini (Squamata: Lacertidae: Lacertinae), with descriptions of eight new genera. *Zootaxa* **1430**, 1–86 (2007).
90. Gauthier, J. A., Kearney, M., Maisano, J. A., Rieppel, O. & Behlke, A. D. Assembling the squamate tree of life: perspectives from the phenotype and the fossil record. *Bulletin of the Peabody Museum of Natural History* **53**, 3–308 (2012).
91. Estes, R., Gauthier, J. & De Queiroz, K. Phylogenetic relationships within Squamata; pp. 119–281 in R. Estes and G. Pregill (eds.), Phylogenetic Relationships of the Lizard Families. Stanford (1988).

92. Feldman, A., Sabath, N., Pyron. R. A., Mayrose, I. & Meiri, S. Body-sizes and diversification rates of lizards, snakes, amphisbaenians and the tuatara. *Global Ecol. Biogeogr.* **25**, 187–197 (2016).
93. Sinervo, B. *et al.* Erosion of lizard diversity by climate change and altered thermal niches. *Science* **328**, 894–899 (2010).
