# Supplementary material for: Environmental temperatures shape thermal physiology as well as diversification and genome-wide substitution rates in lizards
Source: Nat Commun. 2019 Sep 9;10:4077. doi: 10.1038/s41467-019-11943-x (PMC6733905; doi:10.1038/s41467-019-11943-x)
Supplement: Supplementary file 3 — Reporting Summary [file 41467_2019_11943_MOESM3_ESM.pdf]

nature research

Corresponding author(s): Miguel Vences
Last updated by author(s): Jul 25, 2019

Reporting Summary

Nature Research wishes to improve the reproducibility of the work that we publish. This form provides structure for consistency and transparency in reporting. For further information on Nature Research policies, see Authors & Referees and the Editorial Policy Checklist .

Statistics

For all statistical analyses, confirm that the following items are present in the figure legend, table legend, main text, or Methods section.

n/a	Confirmed	
☐	☒	The exact sample size (n) for each experimental group/condition, given as a discrete number and unit of measurement
☐	☒	A statement on whether measurements were taken from distinct samples or whether the same sample was measured repeatedly
☐	☒	The statistical test(s) used AND whether they are one- or two-sided<br>*Only common tests should be described solely by name; describe more complex techniques in the Methods section.*
☐	☒	A description of all covariates tested
☐	☒	A description of any assumptions or corrections, such as tests of normality and adjustment for multiple comparisons
☐	☒	A full description of the statistical parameters including central tendency (e.g. means) or other basic estimates (e.g. regression coefficient) AND variation (e.g. standard deviation) or associated estimates of uncertainty (e.g. confidence intervals)
☐	☒	For null hypothesis testing, the test statistic (e.g. F, t, r) with confidence intervals, effect sizes, degrees of freedom and P value noted<br>*Give P values as exact values whenever suitable.*
☐	☒	For Bayesian analysis, information on the choice of priors and Markov chain Monte Carlo settings
☐	☒	For hierarchical and complex designs, identification of the appropriate level for tests and full reporting of outcomes
☐	☒	Estimates of effect sizes (e.g. Cohen's d, Pearson's r), indicating how they were calculated

Our web collection on statistics for biologists contains articles on many of the points above.

Software and code

Policy information about availability of computer code

Data collection	No specific code or software (beyond commercially available software linked to sequencer machines) was used for data collection.
Data analysis	We used different R packages (specified in the Methods), as well as standard statistical packages (JMP, Statistica).Phylogenetic analyses were made with standard freely software packages.

For manuscripts utilizing custom algorithms or software that are central to the research but not yet described in published literature, software must be made available to editors/reviewers. We strongly encourage code deposition in a community repository (e.g. GitHub). See the Nature Research guidelines for submitting code & software for further information.

Data

Policy information about availability of data

All manuscripts must include a data availability statement. This statement should provide the following information, where applicable:

- Accession codes, unique identifiers, or web links for publicly available datasets
- A list of figures that have associated raw data
- A description of any restrictions on data availability

A full description of data analyses is provided in the electronic supplementary material. A data availability statement is included in the main manuscript file. All raw data are available either in the Supplementary Materials, or in the NCBI Sequence Read archive (Bioproject number provided), Genbank (accession numbers provided), or Figshare (DOIs provided). There is no restriction on data availability.

Field-specific reporting

Please select the one below that is the best fit for your research. If you are not sure, read the appropriate sections before making your selection.

- [x] Life sciences
- [ ] Behavioural & social sciences
- [ ] Ecological, evolutionary & environmental sciences

For a reference copy of the document with all sections, see nature.com/documents/nr-reporting-summary-flat.pdf

Life sciences study design

All studies must disclose on these points even when the disclosure is negative.

Sample size	Sample size in our study was 1 per species, and for phylogenomics we selected species a priori to be representative of lacertid diversity; the rationale behind this choice is given in detail. For the main phylogenetic tree, all species for which data were available were included.
Data exclusions	We did no exclude any samples that met our sample/ sequence criteria. Bacterial sequences identified as potential contaminants were excluded from the RNAseq data, and details of these exclusions are reported in the manuscript (all sequences are available in the raw data that have been made available through SRA - Bioproject number is provided).
Replication	For physiological experiments, experiments were replicated with multiple individuals, and precise sample sizes are given in Supplementary Materials. Detailed raw data with values for each replicate have been made available in Figshare.
Randomization	NA
Blinding	NA

Reporting for specific materials, systems and methods

We require information from authors about some types of materials, experimental systems and methods used in many studies. Here, indicate whether each material, system or method listed is relevant to your study. If you are not sure if a list item applies to your research, read the appropriate section before selecting a response.

Materials & experimental systems

n/a	Involved in the study	
x		Antibodies
x		Eukaryotic cell lines
x		Palaeontology
	x	Animals and other organisms
x		Human research participants
x		Clinical data

Methods

n/a	Involved in the study	
x		ChIP-seq
x		Flow cytometry
x		MRI-based neuroimaging

Animals and other organisms

Policy information about studies involving animals; ARRIVE guidelines recommended for reporting animal research

Laboratory animals	The study did not involve laboratory animals.
Wild animals	A large number of liazards of many different species were used for physiological experiments in this study (792 and 626 individuals for preferred temperature and evaporative water loss experiemnts, respectively). These experiments were non-invasive and not harmful for the animals which were subsequently again released to the wild. A small number (<10) specimens were euthanized using lidocaine for tissue sampling.
Field-collected samples	Tissue samples for DNA extraction and molecular analysis were collected from multiple individuals, typically by preserving a part of the tail which was autotomized by the lizards after capture.
Ethics oversight	Experiments were carried out in many countries and over many years, under changing legislations. Ethics permits were partly included in the collection permits or automatically granted for such non-invasive experiments by universities once researchers were accredited for animal experimentation. Ethics permits for experiments carried out under German law were issued by the LAVES (state of Lower Saxony), and for those under Turkish law by the Ege University Animal Experiments Ethics Committee. A full list of permit numbers is given in the Methods.

Note that full information on the approval of the study protocol must also be provided in the manuscript.
